# 8-weeks aerobic exercise ameliorates cognitive deficit and mitigates ferroptosis triggered by iron overload in the prefrontal cortex of *APP*_Swe_/*PSEN*_1dE9_ mice through Xc^−^/GPx4 pathway

**DOI:** 10.3389/fnins.2024.1453582

**Published:** 2024-09-09

**Authors:** Chaoyang Li, Kaiyin Cui, Xinyuan Zhu, Shufan Wang, Qing Yang, Guoliang Fang

**Affiliations:** ^1^Exercise Biology Research Center, China Institute of Sport Science, Beijing, China; ^2^Sport Science School, Beijing Sport University, Beijing, China; ^3^Department of Medical Supervision, China National Institute of Sports Medicine, Beijing, China; ^4^National Fitness and Scientific Exercise Research Center, China Institute of Sport Science, Beijing, China

**Keywords:** Alzheimer’s disease, aerobic exercise, prefrontal cortex, iron metabolism, ferroptosis, lipid antioxidant, Xc^−^/GPx4 pathway

## Abstract

**Background:**

Alzheimer’s disease (AD) is a degenerative disorder of the central nervous system characterized by notable pathological features such as neurofibrillary tangles and amyloid beta deposition. Additionally, the significant iron accumulation in the brain is another important pathological hallmark of AD. Exercise can play a positive role in ameliorating AD, but the mechanism is unclear. The purpose of the study is to explore the effect of regular aerobic exercise iron homeostasis and lipid antioxidant pathway regarding ferroptosis in the prefrontal cortex (PFC) of *APP*_Swe_/*PSEN*_1dE9_ (APP/PS1) mice.

**Methods:**

Eighty 6-month-old C57BL/6 J and APP/PS1 mice were divided equally into 8-weeks aerobic exercise groups and sedentary groups. Subsequently, Y-maze, Morris water maze test, iron ion detection by probe, Western Blot, ELISA, RT-qPCR, HE, Nissle, Prussian Blue, IHC, IF, and FJ-C staining experiments were conducted to quantitatively assess the behavioral performance, iron levels, iron-metabolism-related proteins, lipid antioxidant-related proteins and morphology in each group of mice.

**Results:**

In APP/PS1 mice, the increase in heme input proteins and heme oxygenase lead to the elevated levels of free iron in the PFC. The decrease in ferritin content by ferritin autophagy fails to meet the storage needs for excess free iron within the nerve cells. Ultimately, the increase of free ferrous iron triggers the Fenton reaction, may lead to ferroptosis and resulting in cognitive impairment in APP/PS1 mice. However, 8-weeks aerobic exercise induce upregulation of the Xc^−^/GPx4 pathway, which can reverse the lipid peroxidation process, thereby inhibiting ferroptosis in APP/PS1 mice.

**Conclusion:**

8 weeks aerobic exercise can improve learning and memory abilities in AD, upregulate GPx4/Xc^−^ pathway in PFC to reduce ferroptosis induced by AD.

## 1 Introduction

Alzheimer’s disease (AD) is a chronic syndrome characterized by cognitive impairment stemming from brain dysfunction, with a higher susceptibility among the elderly, females, and individuals with lower socioeconomic status. The primary clinical manifestations of AD encompass cognitive dysfunction and memory decline (Nelson et al., 2009). Pathologically, AD is predominantly characterized by the neurofibrillary tangles (NFTs) and the accumulation of amyloid beta (Aβ) (Sebastian-Serrano et al., 2018; Hardy and Selkoe, 2002). Despite the fact that the deposition of Aβ and NFTs triggers a cascade of downstream reactions culminating in AD, research suggests the phenomenon of excessive iron deposition in brain cells precedes the onset of Aβ deposition and NFTs (Cheignon et al., 2018; Wan et al., 2019). Moreover, iron overload is implicated in the processes of Aβ deposition and the abnormal phosphorylation of the Tau protein (Cheignon et al., 2018; Wan et al., 2019; Schubert and Chevion, 1995; Jiang et al., 2009). Thus, the imbalance in brain iron metabolism, leading to iron deposition, is likely a pivotal factor initiating the onset of AD.

Iron is a widely distributed essential trace mineral in nature, crucial for the human body. In normal adults, the iron content is approximately 3–5 grams, making it the most abundant trace mineral element in the body. Within living organisms, Fe^2+^ is susceptible to oxidation by physiological oxygen concentrations (O_2_) and hydrogen peroxide (H_2_O_2_) produced during cellular energy metabolism. Facilitated by the Fenton and Haber-Weiss reactions, this oxidation process results in the formation of Fe^3+^, superoxide anions, hydroxyl radicals, and other reactive oxygen species (ROS) (Walling et al., 1975; Singh et al., 2014). ROS have the capability to strip electrons from molecules like proteins, lipids, and DNA within the cell, resulting in damage to their normal structure and function. This damage disrupts regular cellular activities and, in severe cases, can trigger a phenomenon known as ferroptosis in cells. Furthermore, the oxidative stress reaction induced by iron-mediated ROS production stimulates the additional release of iron from iron proteins, heme iron, and iron–sulfur clusters. This establishes a cytotoxic positive feedback loop between iron release and oxidative stress, worsening damage to cellular and tissue structures (Sahlstedt et al., 2001; Pootrakul et al., 2004; MacKenzie et al., 2008). Disruption of iron metabolism, resulting in excessive iron accumulation in the brain, can contribute to various neurodegenerative diseases, including AD, Parkinson’s syndrome, and so on (Ward et al., 2014).

At present, clinically utilized drugs for AD treatment can be classified into two categories: acetylcholinesterase inhibitors and N-methyl-D-aspartate receptor antagonists (Huang and Mucke, 2012; Gauthier et al., 2016). These medications can only partially retard the progression of AD and do not attain a curative effect (Fisher, 2008). Hence, the effective prevention and treatment of AD necessitate not only the ongoing development of new targeted drugs but also the proactive exploration of non-pharmacological intervention methods. In recent years, a wealth of research results has indicated that engaging in scientifically regulated physical exercise can effectively enhance cognitive function and boost learning and memory capabilities (Arrieta et al., 2018; Song and Yu, 2019). The physiological mechanisms by which aerobic exercise prevents and delayed AD occurrence include enhancing mitochondrial function (Bernardo et al., 2016), mitigating cerebral oxidative stress response (Koo and Kang, 2019), reducing abnormal phosphorylation of Tau proteins (Fang et al., 2018), and suppressing neuroinflammatory reactions (Ryan and Kelly, 2016) and so on. On the other hand, the deposition of iron in the hippocampal and neuronal ferroptosis have been identified as the major factors inducing the onset of AD (Hambright et al., 2017; Yan and Zhang, 2020). Iron accumulation in the brain typically also occurs in cortical regions such as the cingulate cortex, frontal cortex, and temporal cortex. High levels of iron in these cortices can accelerate cognitive decline in AD patients (Ayton et al., 2021). Additionally, quantitative susceptibility mapping has shown that patients with cognitive decline and type II diabetes often exhibit elevated iron levels in the frontal cortex (Li et al., 2020). Therefore, iron metabolism imbalance and iron deposition in the prefrontal cortex are likely key factors in the onset of AD.

However, a previous study demonstrated that 6 months of voluntary wheel running can suppress the expression of heme oxygenase (HO1), the key heme metabolism enzyme, in AD model, thereby increasing the formation of Fe^2+^ (Belaya et al., 2021). Thus, heme transporters such as the Feline Leukemia Virus Subgroup C Receptor 2 (FLVCR2) may facilitate the degradation of heme into Fe^2+^, which can enhance the occurrence of the Fenton reaction. Meanwhile, ferritin, a protein responsible for iron storage, can protect neurons against the biotoxicity caused by iron ions. But we found a decrease in ferritin levels in the prefrontal cortex (PFC) of AD model mice, which results in an enhanced Fenton reaction as well. So far, there is no comprehensive report on whether regular aerobic exercise affects heme-metabolism and ferroptosis pathway in the cerebral cortex of APP/PS1 mice. And, previous research on the effects of exercise on brain iron metabolism in AD models has yielded inconsistent results (Belaya et al., 2021; Choi et al., 2021). Specifically, Belaya’s study found that 6 months of voluntary wheel running had no significant effect on iron levels in the cerebral cortex, while Choi’s study found that 8 weeks of treadmill exercise significantly reduced iron levels in the cerebral cortex. Therefore, further research is necessary to explore the impact of exercise on iron metabolism and ferroptosis-related molecules. In this study, we used the *APP*_Swe_/*PSEN*_1dE9_ mice model of AD, aiming to elucidate the molecular mechanisms of aerobic exercise in ameliorating heme metabolism and the lipid antioxidant pathway of ferroptosis in the PFC of AD model. It provides a theoretical foundation and introduces novel perspectives for the prevention and delay of AD through physical exercise in the future.

## 2 Materials and methods

### 2.1 Experimental animals

In this study, we utilized a heterozygous male mouse of B6C3-Tg (*APP_Swe_/PSEN_1dE9_*) 85Dbo/Mmjax strain (APP/PS1), a double transgenic model for AD based on the C57BL/6 J mice background. These mice express chimeric mouse/human amyloid precursor protein (*Mo/HuAPP695Swe*) and mutant human presenilin-1 (*PS_1dE9_*) genes in the neurons of the central nervous system. Mutations in both of these genes are associated with early-onset AD (Zhong et al., 2014). APP/PS1 mice begin to develop Aβ deposits in the brain at 6 to 7 months of age, leading to the manifestation of AD-related symptoms.

Forty 6-month-old male APP/PS1 transgenic mice and forty 6-month-old male C57BL/6 J wild type mice were procured from Zhishan (Beijing) Institute of Health Medicine Co., Ltd. After one week of adaptive breeding, the mice were divided into four groups based on body weight: C57BL/6 J for sedentary (WT-S, *n* = 20, weight = 27.95 ± 1.63 grams), C57BL/6 J for 8-weeks aerobic exercise (WT-E, *n* = 20, weight = 27.30 ± 1.75 grams), APP/PS1 for sedentary (AD-S, *n* = 20, weight = 30.63 ± 3.06 grams), and APP/PS1 for 8-weeks aerobic exercise (AD-E, *n* = 20, weight = 30.67 ± 3.31 grams). All mice had *ad libitum* access to standard rodent chow and water, with a 12: 12 h light–dark cycle, and were kept at 20(±2) °C and 40–60% humidity. This study adhered to the guidelines for the care and use of animals in China and institutional ethical guidelines. All animal experiments were reviewed and approved by the Ethics Committee of the China Institute of Sport Science (Approval Number: CISS-20231229).

### 2.2 Exercise protocol

**Figure 1:**
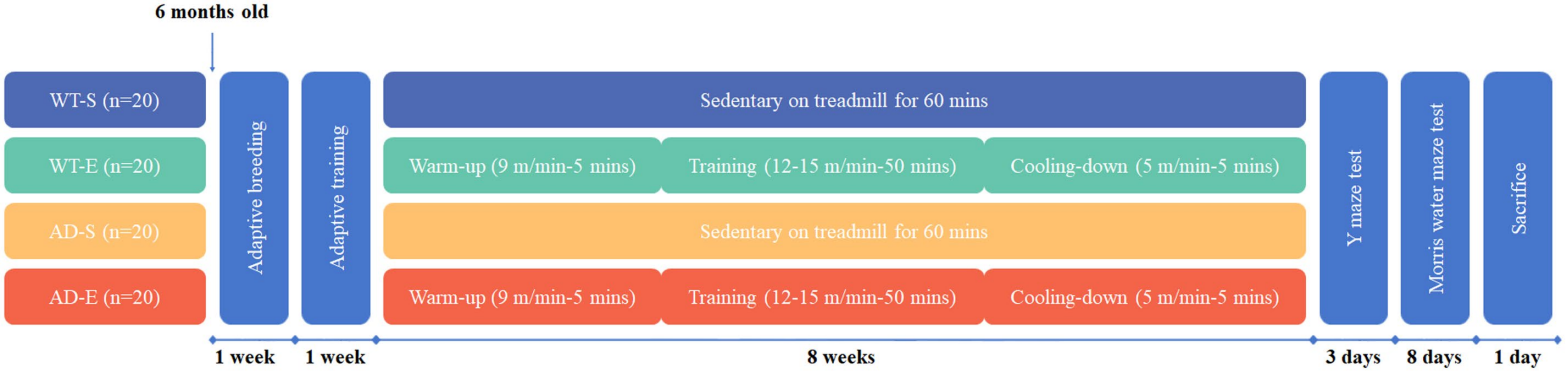
The design of aerobic exercise for animal modeling.

An 8-week aerobic exercise intervention was implemented for the WT-E and AD-E groups, consisting of 60 min of exercise per day at 12–15 meters/min (75–80% VO_2max_) (Fernando et al., 1993). After a 5-day adaptation period with 10 min of treadmill training at 9 meters/min and 0° incline, each mouse in the WT-E and AD-E groups underwent formal training. The exercise sessions were conducted Monday to Friday at 6:00 PM. under semi-dark conditions, with weekends off. Each session included a 5 min warm-up at 9 meters/min, 50 min of main training at 12–15 meters/min, and 5 min cooling-down at 5 meters/min. The speed during the main training phase increased by 1 meter/min every 2 weeks until reaching 15 meters/min by the 6th week, then remained constant (Figure 1). After each exercise session was completed, alcohol was used to wipe down the treadmill to remove any residues, such as feces and urine.

### 2.3 Y-maze novel arm test

Y-maze novel arm test was employed to assess learning and spatial working memory abilities of each group of mice. The Y-maze novel arm experiment, with dimensions of 300 × 50 × 120 mm, was conducted over a period of 3 days under semi-dark conditions. A high-definition behavioral recording camera was placed directly above the center of the Y-maze. The metrics were quantified using Labmaze animal behavior analysis software V 3.0 (Zhongshi Technology Development Co., Ltd., Beijing, China). Each time the Y-maze test was completed, alcohol was used to wipe down the maze to avoid interference from the scent of the previous mouse and to remove any residues, such as feces and urine. After cleaning, we allowed the alcohol to fully evaporate for 5 min before testing the next animal. The complete Y-maze novel arm experiment consisted of the following two parts:

Spontaneous alternation task: On the first day, mice were individually placed at the end of arm A of Y-maze, and allowed to freely explore arms A, B, and C for 10 min. The video recording system captured the number of spontaneous alternations and total entries into each arm within the 10 min period. The spontaneous alternation rate was calculated using the formula: Spontaneous alternation rate = [(number of spontaneous alternations) /(total number of arm entries − 2)] × 100% (Kraeuter et al., 2019).

Spatial cognitive experiment: On the second day in the morning, a training session was conducted where the novel arm was blocked off. The mice were individually placed at the end of start arm of the Y-maze allowing the animals to freely explore the start and open arms for 5 min. Two hours later, the novel arm was open, allowing mouse access to all three arms for 5 min. The activity time, distance traveled, exploration distance, latency, and the number of entries into each arm during the exploration period were recorded by high-definition camera. The formula for the discrimination index is the exploration time or distance in the novel arm divided by the total exploration time or distance in all three arms.

### 2.4 Morris water maze test

The Morris Water Maze (MWM) test was used to assess spatial learning and memory abilities. The MWM test was conducted under light conditions in a water pool with a diameter of 1,200 mm, a height of 50 mm, and a water temperature of 21°C. The complete MWM test consisted of two parts:

Spatial learning test: Mice were placed in the water maze facing the pool wall, entering from one of four quadrants. Escape latency was recorded, and if a mouse failed to find the platform within 60 s, it was guided to stand on the platform for 10 s and then the latency was recorded as 60 s. The MWM test for every mouse was conducted four times a day at 15 min intervals over 7 days.

Spatial memory test: On the 8th day, the platform was removed. Mice were placed in the water facing the pool wall, and the number of times they crossed the original platform location within 1 min, the time spent in the platform quadrant, and the escape latency were recorded by high-definition camera. The metrics were quantified using Labmaze animal behavior analysis software V 3.0 (Zhongshi Technology Development Co., Ltd., Beijing, China).

### 2.5 Sacrificed and tissue collection

After all behavioral tests were completed, all mice were fasted for 12 h. Subsequently, they were anesthetized with isoflurane inhalation and then decapitated to obtain the brain. The bilateral PFC of the brain was collected, and all procedures were performed on ice. The samples were temporarily stored in liquid nitrogen and then transferred to a − 80°C ultra-low temperature freezer for long-time preservation.

### 2.6 Western blot

Take 50 mg of PFC tissue samples from each group of mice and place them into glass homogenizers. Quickly add 1 mL lysis buffer along with 10 μL of 10 mM PMSF proteinase inhibitor. Grind and lyse the tissue thoroughly, then transfer it to 1.5 mL EP tubes. Centrifuge the EP tubes in a centrifuge at 10,000 × g, 4°C for 10 min. Transfer the supernatant to new EP tubes and take 10 μL of the supernatant for protein concentration determination using the BCA method. Add the remaining supernatant to an appropriate amount of 5 × protein loading buffer at a ratio of 4:1, and heat it in a 100°C constant temperature metal bath for 5 min for protein denaturation. Subsequently, perform SDS-PAGE electrophoresis, transfer the proteins to a polyvinylidene fluoride (PVDF) membrane, block the membrane with 5% skim milk (dissolved in TBST solution) at room temperature for 1 h. Incubate the membrane with appropriately diluted primary antibodies (diluted in TBST solution containing 1% skim milk) at 4°C overnight. The primary antibodies and dilution ratios used include BACE1 (ABclonal, A11533, 1:1000), FtL (ABclonal, A11241, 1:1000), FtH (ABclonal, A19544, 1:1000), FLVCR2 (ABclonal, A7814, 1:1000), ABCG2 (ABclonal, A5661, 1:1000), NCOA4 (ABclonal, A5895, 1:500), APP (Elabscience, E-AB-15509, 1:1000), HO1 (Beyotime, AG2181, 1:1000), Nrf_2_ (Beyotime, AF7623, 1:1000), GPx4 (Beyotime, AF7020, 1:1000), β-actin (Beyotime, AA128, 1:1000), and Xc^−^ (Thermo Fisher Scientific, PA1-16893, 1:1000). The next day, wash the membrane 3 times with TBST for 5 min each to remove residual primary antibodies. Then, incubate the membrane with the secondary antibodies of diluted HRP-labeled goat anti-mouse IgG (Beyotime, A0216, 1:2000) or anti-rabbit IgG (Beyotime, A0208, 1:2000) at room temperature for 1 h (secondary antibody diluted in TBST solution containing 1% skim milk). Wash the membrane 3 times with TBST for 5 min each to remove residual secondary antibodies. Finally, use ECL chemiluminescent reagent and a Bio-Rad gel imaging system to detect the protein signals. Analyze the grayscale intensity of target proteins using Image Lab professional software. The relative content of the target protein was calculated by dividing the expression level of the target protein by the expression level of β-actin.

### 2.7 Real-time quantitative polymerase chain reaction

Total RNA from the PFC tissue samples was extracted using the TRIzol-chloroform-isopropanol method. Take 50 mg of PFC tissue samples from each group of mice and place them into glass homogenizers. Add 1 mL of TRIzol for lysis and sample homogenization thoroughly, and incubate for 5 min to allow the protein-RNA complex to fully dissolve. Add 0.2 mL of chloroform, cover, and shake vigorously for 10 s and incubate for 2–3 min. Centrifuge the sample for 15 min at 12000 × g, 4°C. After centrifugation, tilt the EP tube at a 45° angle to transfer the top aqueous layer to a new EP tube for RNA extraction. Add 0.5 mL of isopropanol to the aqueous layer to gently mix and incubate at 4°C for 10 min. Centrifuge the sample for 10 min at 12000 × g, 4°C. Then completely discard the supernatant, retaining the RNA pellet. Remove any residual liquid with a pipette. Add 1 mL of 75% ethanol to resuspend the RNA pellet. Briefly vortex the EP tube and centrifuge at 7500 × g, 4°C, for 5 min. Completely discard the supernatant, retaining the RNA pellet. Remove any residual liquid with a pipette. Open the cap and air-dry the RNA pellet at room temperature for 5–10 min. Add 20–50 μL of RNase-free water to resuspend the RNA pellet. Incubate in a heat block at 55°C for 10 min to fully dissolve the RNA. Subsequently, the total RNA concentration of each sample was determined using an ultraviolet spectrophotometer and normalized. All of the aforementioned procedures were performed on ice. Then, 20 μL reverse transcription system was constructed to obtain cDNA. The nucleotide sequences of the target genes were obtained from the NCBI and Primer Bank databases, and forward and reverse primers were designed (Table 1). After obtaining the primers, 5 μL of SYBR Green fluorescent dye, 0.2 μL of each forward and reverse primer (concentration 10 μM), 1 μL of cDNA, and 3.6 μL of Nase/RNase-free water were added to each reaction well to construct a 10 μL system for real-time quantitative polymerase chain reaction (RT-qPCR). The following 40 cycling conditions were set: pre-denaturation at 95°C for 30 s, denaturation at 95°C for 15 s, annealing at 60°C for 30 s, extension at 72°C for 30 s. Fluorescence signals were collected immediately after the extension step. Finally, the relative expression levels of the target genes were calculated using the formula 2^^(-ΔΔCt)^ based on the Ct values of each reaction well.

**Table 1:** The nucleotide sequences of primers in RT-qPCR.

Primer names	Nucleotide sequences (5′-3′)
*FtL* Forward	CCTGTGGCTGATGGTGGAGTTG
*FtL* Reverse	GGAGATTGATGGCGATGGCTGAG
*HO1* Forward	AAGCCGAGAATGCTGAGTTCA
*HO1* Reverse	GCCGTGTAGATATGGTACAAGGA
*β-actin* Forward	CATGTACGTTGCTATCCAGGC
*β-actin* Reverse	CTCCTTAATGTCCGCACGAT

### 2.8 Enzyme linked immunosorbent assay

The Aβ_40_ expression in the PFC tissue of mice was determined using mouse Aβ_1–40_ sandwich enzyme linked immunosorbent assay (ELISA) kit (Elabscience, E-EL-M3009). Adequate amounts of PFC tissue samples from each group of mice were placed into 1.5 mL EP tubes. PBS was added to each EP tube at a ratio of 9 mL per gram of tissue, followed by the addition of PMSF proteinase inhibitor at a volume ratio of 1:100, and the mixture was ground by electric homogenizer. After thorough grinding and lysis, the mixture was transferred to 1.5 mL EP tubes and centrifuged at 5000 × g, 4°C for 5 min, and the supernatant was extracted. The reagents were equilibrated to room temperature (25–28°C) for 20 min, and washing solution, dilution solution, and standards at different concentrations were prepared. Standards and tissue samples (100 μL each) were added to a 96-well ELISA plate and incubated at 37°C for 90 min. The liquid in the wells was then removed, and 100 μL of biotinylated detection Ab working solution was added, followed by incubation at 37°C for 60 min. After washing three times, 100 μL of HRP-conjugated working solution was added, and the plate was incubated at 37°C in the dark for 30 min. After washing five times, 90 μL of substrate reagent was added, and the plate was incubated at 37°C in the dark for 15 min. Finally, stop solution was added, and the OD value was immediately read at a wavelength of 450 nm using a microplate reader. A standard curve was constructed to calculate the concentration of the target protein in the samples.

### 2.9 Tissue perfusion and paraffin section

The mouse was continuously anesthetized with isoflurane. The heart was exposed and perfused with 50 mL of 0.9% saline for 5 min, followed by 50 mL of 4% paraformaldehyde for 5 min. Fresh brains were dissected and immersed in 4% paraformaldehyde for 24 h. The tissue was dehydrated in a series of graded alcohols: 75% ethanol for 4 h, 85% ethanol for 2 h, 90% ethanol for 2 h, 95% ethanol for 1 h, absolute ethanol I and II for 30 min each, ethanol-benzene for 5 min, xylene I and II for 5 min each, and melted paraffin I, II, and III for 1 h each. The tissue was then embedded in melted wax and placed in an embedding mold. After cooling on a − 20°C freezing platform, the wax block was trimmed and sectioned on a paraffin microtome at 4 μm thickness. Sections were floated on a 40°C water bath, mounted on glass slides, and baked in a 60°C oven. Sections were stored at room temperature for later use.

### 2.10 HE staining

The paraffin sections were sequentially immersed in Environmentally Friendly Dewaxing Transparent Liquids I and II for 20 min each, anhydrous ethanol I and II for 5 min each, 75% ethanol for 5 min, and then rinsed with tap water. The sections were treated with HD constant staining pretreatment solution for 1 min, followed by Hematoxylin solution for 5 min, and rinsed with tap water. They were then treated with Hematoxylin Differentiation and Bluing solutions, each followed by a rinse with tap water. The sections were placed in 95% ethanol for 1 min, Eosin dye for 15 s, and then in absolute ethanol I, II, and III for 2 min each. Finally, they were immersed in normal butanol I and II for 2 min each, xylene I and III for 2 min each, and sealed with neutral balsam.

### 2.11 Nissl staining

The paraffin sections were immersed in sequence in Environmentally Friendly Dewaxing Transparent Liquid I for 20 min, Environmentally Friendly Dewaxing Transparent Liquid II for 20 min, Anhydrous ethanol I for 5 min, Anhydrous ethanol II for 5 min, and 75% Ethyl alcohol for 5 min, and then rinsed with tap water. Treat the tissue slices with dye solution for 5 min, rinse with tap water. 0.1% glacial acetic acid slightly differentiated, the reaction was terminated by running water washing, and the differentiation degree was controlled under the microscope. Wash it with tap water, dry it in the oven. Xylene transparent for 10 min, and seal with neutral balsam.

### 2.12 Prussian blue staining

Place the paraffin sections in sequence in Xylene I for 20 min, Xylene II for 20 min, Anhydrous ethanol I for 5 min, Anhydrous ethanol II for 5 min, and 75% Ethyl alcohol for 5 min, washing with tap water and distilled water 3 times. Mix Prussian blue staining solution A and Prussian blue staining solution B in equal proportions to get Prussian blue staining solution, put the slides into staining solution staining for 1 h, and wash twice with distilled water. Prussian blue staining solution C staining for 3 min, running water washing. Put the sections into absolute ethanol I 5 min, absolute ethanol II 5 min, absolute ethanol III 5 min, xylene I 5 min, xylene II 5 min for transparency, sealing with neutral balsam.

### 2.13 Immunohistochemistry

Aβ_42_ was detected using immunohistochemistry (IHC) for pathological morphology assessment. The sections were sequentially placed in Environmentally Friendly Dewaxing Solutions I and II for 10 min each, anhydrous ethanol I, II, and III for 5 min each, and rinsed with distilled water. Antigen retrieval was performed in citrate buffer (pH 6.0) using microwave heating (8 min medium power, 8 min cooling, 7 min medium-low power). The sections were incubated in 3% hydrogen peroxide at room temperature in the dark for 25 min, followed by washing in PBS (pH 7.4) 3 times for 5 min each. The tissue was then covered with 3% BSA solution and incubated at room temperature for 30 min. After removing the blocking solution, the sections were incubated overnight at 4°C with a 1:1000 dilution of the primary antibody (beta Amyloid_1–42_, ab201060, Abcam) in a humidified chamber. Following this, the sections were washed in PBS 3 times for 5 min each, air-dried, and covered with an HRP-conjugated goat anti-rabbit IgG secondary antibody, then incubated at room temperature for 50 min. After secondary antibody incubation, the sections were washed in PBS 3 times for 5 min each. The sections were then incubated with DAB staining solution until positive staining appeared brownish-yellow under a microscope, followed by rinsing with tap water. Nuclei were counterstained with hematoxylin for 3 min, washed with tap water, differentiated in hematoxylin differentiation solution, rinsed, and blued in hematoxylin bluing reagent. Finally, the sections were dehydrated and made transparent by placing them sequentially in 75% ethanol for 5 min, 85% ethanol for 5 min, anhydrous ethanol I and II for 5 min each, n-butanol for 5 min, and xylene I for 5 min. After dehydration and transparency, the sections were air-dried and mounted with neutral resin.

### 2.14 Immunofluorescence

4-Hydroxynonenal (4-HNE) and HO1 were subjected to immunofluorescence double staining for pathological localization. The sections were sequentially immersed in Environmentally Friendly Dewaxing Solutions I, II, and III for 10 min each, anhydrous ethanol I, II, and III for 5 min each, and then rinsed with distilled water. Citrate buffer (pH 6.0) was boiled and used to heat the sections for 2 min, followed by cooling with tap water. The sections were incubated in 3% hydrogen peroxide at room temperature for 25 min, washed in PBS (pH 7.4) 3 times for 5 min each, and blocked with 3% BSA solution at room temperature for 30 min. After removing the blocking solution, the sections were incubated overnight at 4°C with diluted 4-HNE (Abcam, ab48506, 1:100) or GPx4 (Servicebio, GB154327, 1:2000) primary antibody. The sections were then washed in PBS 3 times for 5 min each, air-dried, and incubated with HRP-conjugated secondary antibody at room temperature for 50 min. After washing in PBS 3 times for 5 min each, TSA dye (488-Tyramide, Servicebio, G1231) was applied and incubated at room temperature for 10 min in the dark. The sections were washed in TBST 3 times for 5 min each. The same steps were repeated for HO1 (Servicebio, GB15014, 1:2000) or NeuN (Servicebio, GB15138, 1:5000) primary antibody, followed by the corresponding secondary antibody and TSA (555-Tyramide, Servicebio, G1233). The sections were air-dried, counterstained with DAPI, incubated at room temperature for 10 min in the dark, and washed in PBS 3 times for 5 min each. After slightly drying, spontaneous fluorescence quenching reagent B solution was added, incubated for 5 min, and rinsed with flowing water for 10 min. Finally, the sections were mounted with anti-fluorescence quenching mounting solution and cover-slipped. The corresponding excitation wavelength was used to excite the TSA dye.

### 2.15 Fluoro-Jade C staining

In this study, neuronal degeneration was assessed using immunofluorescent staining with NeuN and FJ-C to evaluate pathological changes. The primary antibody, the secondary antibody, and the TSA staining steps (555-Tyramide, Servicebio, G1233) for NeuN (Servicebio, GB15138) were the same as those used for immunofluorescent staining. For FJ-C staining, the following steps were followed: Tissue sections were air-dried on a slide in a 50°C oven for 30 min and then immersed in distilled water for 2 min. The FJ-C staining kit (Biosensis, TR-100-FJT) was used for staining. Dilution solution B was prepared by mixing distilled water and Solution B (potassium permanganate) at a ratio of 9:1. Tissue sections were immersed in dilution solution B for 5 min and then rinsed thoroughly with distilled water for 2 min to complete washing. In a dark environment, dilution solution C was prepared by mixing distilled water, Solution C (FJ-C dye), and Solution D (DAPI) at ratio of 9:1:1. Tissue sections were immersed in dilution solution C for 10 min and then washed three times with distilled water for 1 min each time. The sections were then air-dried at 50°C in the dark for 5 min. A 5 min in xylene was performed, followed by mounting the sections with neutral mounting medium.

### 2.16 Iron ion colorimetric assay

The reagent kit was equilibrated at room temperature (25–28°C) for 20 min in advance. Buffer solution, color development solution, standard solution protective solution, and extraction agent, as well as iron standard solutions of different concentrations, were prepared. Subsequently, appropriate amounts of cortical samples from each group of mice were placed into glass homogenizers, and extraction agent was added for dilution and homogenization. The mixture was then transferred into 1.5 mL EP tubes and centrifuged at 11400 × g for 10 min at 4°C, followed by extraction of the supernatant. Following the instructions, color development solution was added to different concentrations of iron ion standard solutions and each sample, mixed well, and then incubated at 37°C for 10 min for ferrous test or 40 min for total iron test. After centrifugation at 11400 × g for 10 min, 300 μL of the supernatant was taken and transferred into each corresponding well of the 96-well plate. The OD values of each well were measured at 593 nm using a microplate reader. Standard curves were constructed to calculate the concentrations of total iron and ferrous iron in the test samples.

### 2.17 Statistical analysis

This study conducted statistical analysis using SPSS Statistics 26.0 or R Studio software and generated graphs using Prism GraphPad 8.0 software. A significance level of *p* ≤ 0.05 indicated a significant difference, while 0.05 < *p* ≤ 0.1 was considered marginally significant. Descriptive statistics were presented as mean ± standard deviation (SD) for normally distributed data or median (interquartile range) for skewed data. For latency data obtained from the MWM, a three-way repeated measures ANOVA (time * exercise mode * genotype) was used, with the Greenhouse–Geisser correction applied for violations of sphericity. Continuous data with normally distributed were analyzed using two-way ANOVA (exercise mode * genotype). If interaction effects were present, *post hoc* tests were conducted using the least significant difference (LSD) method; if no interaction effects were found, main effects were examined.

## 3 Results

### 3.1 8 weeks of aerobic exercise improves learning and memory abilities in APP/PS1 mice

**Figure 2:**
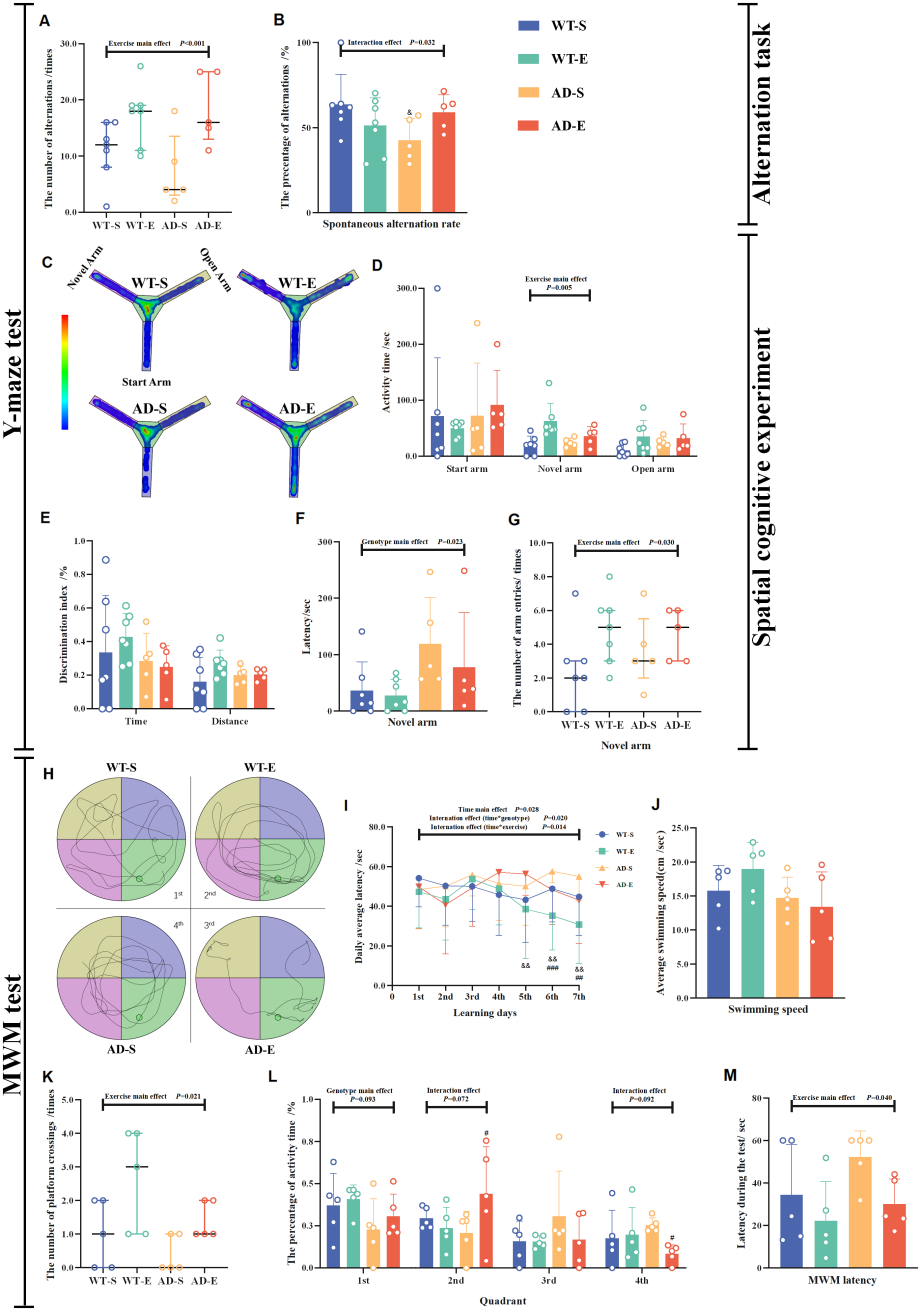
Impaired learning and memory abilities in APP/PS1 mice enhanced by 8 weeks of aerobic exercise. The number of alternations **(A)** and spontaneous alternation rate **(B)** were measured in the Y-maze spontaneous alternation test. Behavioral heat maps of each group were recorded during the Y-maze spatial cognitive experiment **(C)**. The activity time in each arm **(D)**. Discrimination index for time and distance **(E)**, latency **(F)**, and number of entries **(G)** for the novel arm were measured during the Y-maze spatial cognition test on the second day. Behavioral trajectory maps of mice in each group for MWM **(H)**. Latency to find the platform for each group during the 7-day learning period in the MWM **(I)**. During the MWM test period, the average swimming speed **(J)**, the number of platform crossings **(K)**, the percentage of activity time in each quadrant **(L)**, and escape latency **(M)**. In cases of significant interaction effect, “&” denotes significant genotype simple effect between WT-S and AD-S with *p* ≤ 0.05 while “#” indicates a significant exercise simple effect between AD-S and AD-E with *p* ≤ 0.05.

On the first day of the Y-maze test, all arms were opened, and spontaneous alternation behavior was assessed to evaluate the mice’s spatial working memory capability based on the number of spontaneous alternations and the spontaneous alternation rate. The results of the spontaneous alternation experiment indicated a significant main exercise effect (*p* < 0.001, Figure 2A). Regardless of the mouse genotype, the number of spontaneous alternations in the exercise group was significantly higher than that in the sedentary group. However, the results for the spontaneous alternation rate showed a significant interaction effect [*F*(1, 20) = 5.327, *p* = 0.032, η^2^ = 0.332, Figure 2B]. Further LSD simple effect tests revealed a significant genotype simple effect, with the AD-S group exhibiting a significantly lower spontaneous alternation rate than the WT-S group [*F*(1, 20) = 5.709, *p* = 0.027, η^2^ = 0.222, Figure 2B]. On the second day, in the training session of the Y-maze test, the novel arm was closed, and 2 h after the end of the test, the novel arm was reopened. Mice’s spatial recognition memory was assessed based on the latency for novel arm, activity time (the time spent for exploring each arm), discrimination index, and number of entries into the novel arm. The Y-maze exploration heatmaps for each group of mice are shown in Figure 2C. The results of the spatial cognitive experiment on the second day showed a significant exercise main effect on the activity time in novel arm [*F*(1, 20) = 9.959, *p* = 0.005, η^2^ = 0.332, Figure 2D], and number of entries into the novel arm (*p* = 0.030, Figure 2G). Regardless of the mouse genotype, these indicators were significantly increased in the aerobic exercise group compared to the sedentary group. Meanwhile, there was a significant genotype main effect on the latency to find the novel arm [*F*(1, 20) = 6.066, *p* = 0.023, η^2^ = 0.233, Figure 2F]. This indicates that regardless of whether the mice underwent exercise intervention, the APP/PS1 mice took longer to find the novel arm for the first time compared to the C57BL/6 J mice. In APP/PS1 mice, although there was a trend towards decreased discrimination indices for time and distance in the novel arm, no significant effects were found (*p* > 0.050, Figure 2E).

In order to investigate whether aerobic exercise affects the cognitive behavior and memory of mice, the MWM experiment was conducted after the completion of the Y-maze novel arm test. The learning capacity and spatial working memory ability of mice were evaluated by measuring the number of platform crossings, the activity time in the quadrant where platform located, and the escape latency in the MWM test. The changes in escape latency of mice in each group during the 7-day learning period of the MWM experiment are shown in Figure 2I. The results of the three-factor ANOVA for escape latency data during the 7-day period indicated that the escape latency data did not pass the Mauchly’s sphericity test (*p* = 0.025), therefore the Greenhouse Geisser correction was applied to correct the degrees of freedom for repeated measurements of escape latency data. The corrected escape latency data showed no significant second-order interaction effect [*F*(5.272, 400.666) = 0.831, *p* = 0.534, η^2^ = 0.011, Figure 2I], but significant first-order interaction effect of time * exercise [*F*(5.272, 400.666) = 2.848, *p* = 0.014, η^2^ = 0.036, Figure 2I], interaction effect of time * genotype [*F*(5.272, 400.666) = 2.665, *p* = 0.020, η^2^ = 0.034, Figure 2I] and time main effect [*F*(5.272, 400.666) = 2.665, *p* = 0.028, η^2^ = 0.032, Figure 2I] were observed. LSD simple effect tests for the first-order interaction effect of time*exercise showed that on the 6th day [*F*(1, 76) = 11.372, *p* = 0.001, η^2^ = 0.130, Figure 2I] and the 7th day [*F*(1, 76) = 9.770, *p* = 0.003, η^2^ = 0.114, Figure 2I], there were significant exercise simple effect, with the escape latency of the exercise group significantly lower than that of the sedentary group. As for the first-order interaction effect of time*genotype, LSD simple effect tests demonstrated that on the 5th [*F*(1, 76) = 7.647, *p* = 0.007, η^2^ = 0.091, Figure 2I], 6th [*F*(1, 76) = 10.306, *p* = 0.002, η^2^ = 0.119, Figure 2I] and 7th [*F*(1, 76) = 7.321, *p* = 0.008, η^2^ = 0.088, Figure 2I] day, there were significant genotype simple effect, with the escape latency of the APP/PS1 mice significantly higher than that of the C57BL/6 J mice. The specific movement trajectories of mice in each group during the testing period are shown in Figure 2H. The spatial memory experiment of MWM on the 8th day revealed that there was no any significant main effect of exercise, genotype and interaction effect on the swimming speed (*p* > 0.050, Figure 2J). Meanwhile, the Poisson log-linear regression analysis of the number of platform crossings showed a significant exercise main effect (*p* = 0.021, Figure 2K), with the number of platform crossings of exercise groups being higher than that of sedentary groups. There was a marginally significant main genotype effect [*F*(1, 16) = 3.191, *p* = 0.093, η^2^ = 0.166, Figure 2L] on the percentage of activity time in 1st quadrant (the quadrant with platform). The percentage of activity time in 1st quadrant of APP/PS1 mice was lower than that of C57BL/6 J mice. Additionally, there were marginally significant interaction effects on the percentage of activity time in 2nd [*F*(1, 16) = 3.722 *p* = 0.072, η^2^ = 0.189, Figure 2L] and 4th [*F*(1, 16) = 3.203, *p* = 0.092, η^2^ = 0.167, Figure 2L] quadrant. Further LSD tests showed, the percentage of activity time in 2nd quadrant of AD-E group was higher than that of AD-S group [*F*(1, 16) = 4.806, *p* = 0.044, η^2^ = 0.231, Figure 2L], and the percentage of activity time in 4th quadrant in AD-E was lower compared to AD-S [*F*(1, 16) = 5.085, *p* = 0.038, η^2^ = 0.241, Figure 2L]. Furthermore, a significant exercise main effect [*F*(1, 16) = 4.978, *p* = 0.040, η^2^ = 0.237, Figure 2M] on the testing escape latency was also observed, with the escape latency of exercise groups being higher than that of sedentary groups.

### 3.2 8 weeks of aerobic exercise alleviates neuronal degeneration and abnormalities in APP/PS1 mice

**Figure 3:**
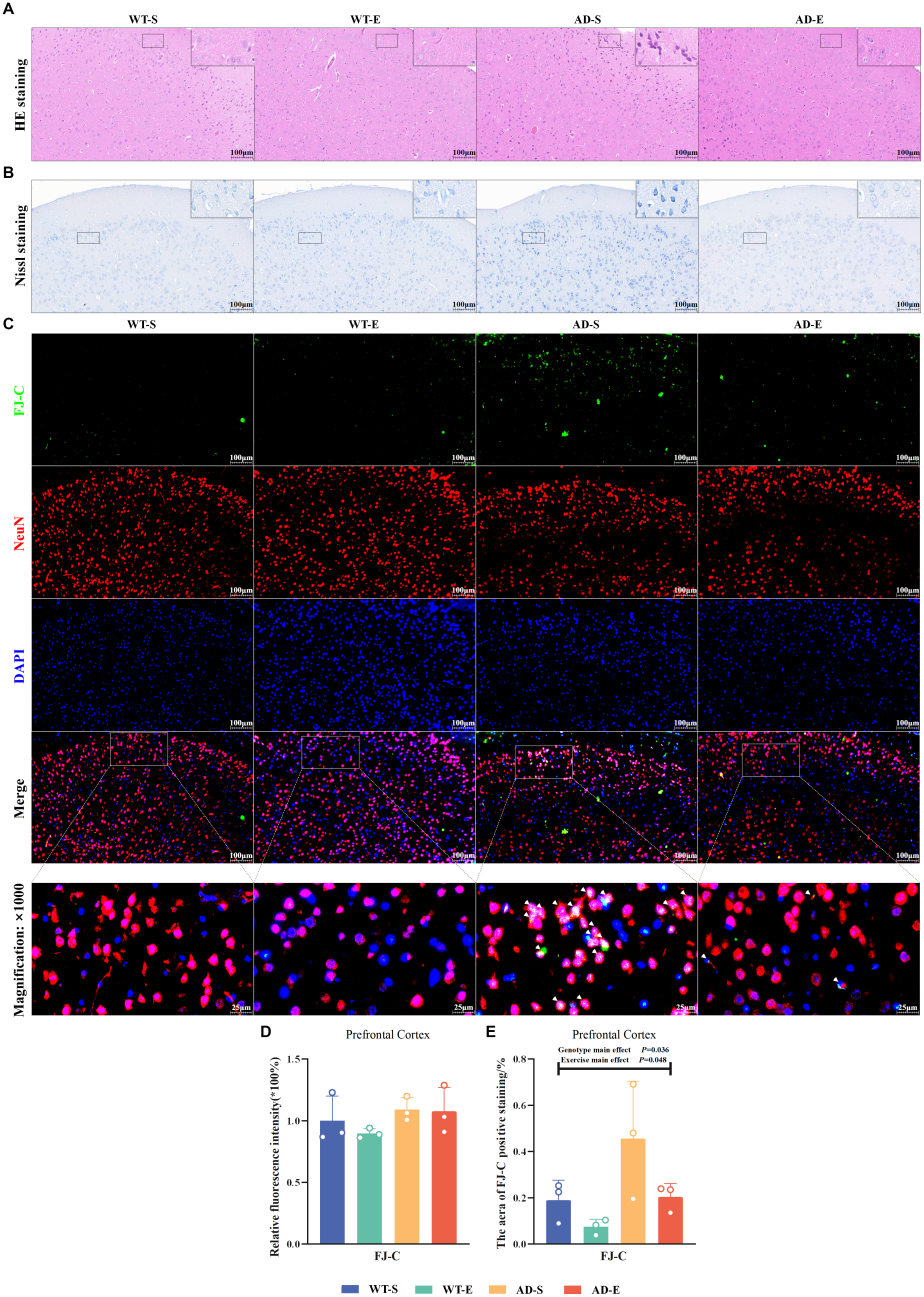
Increased neuronal degeneration and abnormalities in APP/PS1 mice ameliorated by 8 weeks of aerobic exercise. Representative images of HE staining **(A)** and Nissl staining **(B)** in PFC for each group are shown at 250× magnification (insets at 2500× magnification). Representative IF staining images of the PFC at 250× magnification showing FJ-C (green), NeuN (red), DAPI nuclear marker (blue), and triple Merge (composite image at 250× magnification above, 1,000× magnification below) for each group **(C)**. Relative fluorescence intensity **(D)** and positive area **(E)** for FJ-C. In Nissl staining, Nissl bodies appear as dark blue granular structures, and cytoplasm appears light blue. In HE staining, nuclei appear blue-purple, and cytoplasm appears light red. In **(C)**, white arrows indicate colocalization of FJ-C with NeuN or DAPI within cells.

Neuronal atrophy and nuclear condensation are underlying pathological reasons for the decline in cognitive abilities in AD. In this study, the morphological effects of 8-week aerobic exercise on brain cells of APP/PS1 mice were evaluated using HE staining and Nissl staining to assess the degree of cell atrophy and nuclear condensation. In the PFC, HE staining revealed the nuclei of cells in the WT-S, WT-E, and AD-E groups were clearly visible, with distinct nuclear-cytoplasmic boundaries and orderly cell arrangement (Figure 3A). In contrast, cells in the AD-S group showed cytoplasmic deep staining due to diffuse basophilic aggravation, disorganized cell arrangement, disappearance of nuclear dissolution, blurred cytoplasmic boundaries, cell atrophy, nuclear condensation, and neurofibrillary tangles (Figure 3A). In Nissl staining, cells in PFC in the AD-S group showed cytoplasmic deep staining, widespread deep staining areas, and dissolution of Nissl bodies (Figure 3B).

To investigate the impact of an 8-week aerobic exercise intervention on the number of degenerated neurons, this study utilized FJ-C staining to label degenerated cells in the PFC, and used NeuN and DAPI staining to mark neurons, thus assessing neuronal degeneration. In the PFC, the green channel of FJ-C (Figure 3C) was isolated using Image J software, and the fluorescence quantification results of FJ-C immunofluorescence staining were analyzed by selecting a threshold range of 50 to 255. The analysis revealed no significant effects on FJ-C relative fluorescence intensity (*p* > 0.050, Figure 3D), However, there were significant main effects of exercise [*F*(1, 8) = 5.438, *p* = 0.048, η^2^ = 0.405, Figure 3E] and genotype [*F*(1, 8) = 6.322, *p* = 0.036, η^2^ = 0.441, Figure 3E] on the area of FJ-C positive staining. The area of FJ-C positive staining was significantly higher in APP/PS1 mice compared to C57BL/6 J mice, whereas the 8-week aerobic exercise intervention reduced the FJ-C fluorescence staining area.

### 3.3 8 weeks of aerobic exercise decreases Aβ_42_ deposition in APP/PS1 mice

**Figure 4:**
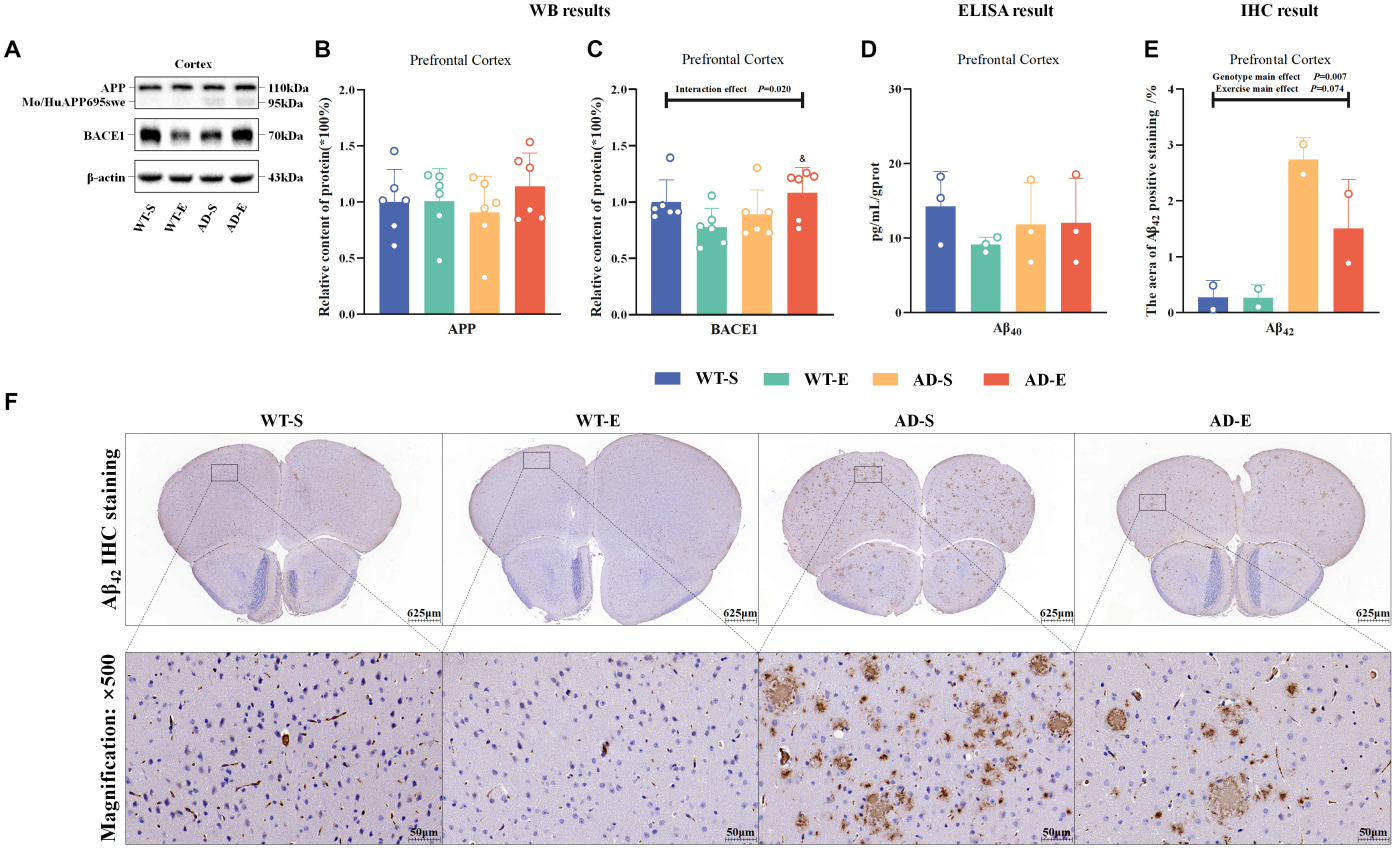
Reduction of Aβ_42_ deposition in APP/PS1 mice following 8 weeks of aerobic exercise. Western blot protein signals of the PFC in each group **(A)**. Relative protein expression levels of APP **(B)** and BACE1 **(C)** in the PFC of each group as detected by WB. ELISA detection of Aβ_40_ in the PFC of each group **(D)**. Statistical results of the area percentage of Aβ_42_ antigen-positive regions in IHC staining of the PFC **(E)**, and tissue morphology images at 40× (upper) and 500× (lower) magnification **(F)**. In cases of significant interaction effect, “&” indicates a significant genotype simple effect between AD-E and WT-E with *p* ≤ 0.05. In **(F)**, hematoxylin stains nuclei blue, and DAB reveals Aβ_42_ antigen-positive expression in brown.

This study measured the relative protein expression of amyloid precursor protein (APP) and β-secretase (BACE1) in the PFC of the brain through WB. Western blot protein signals of the PFC in each group are shown in Figure 4A. In the PFC, the results showed there were no any significant effects on the relative content of APP protein (*p* > 0.050, Figure 4B). There was a significant interaction effect on the relative content of BACE1 protein [*F*(1, 20) = 6.412, *p* = 0.020, η^2^ = 0.243, Figure 4C]. Further LSD simple effect tests revealed a significant genotype simple effect on BACE1 expression in the AD-E group [*F*(1, 20) = 6.891, *p* = 0.016, η^2^ = 0.256, Figure 4C], with a significant increase compared to the WT-E group. There were no significant effects on the content of Aβ_40_ (*p* > 0.050, Figure 4D). There was a significant genotype main effect on the Aβ_42_ antigen-positive area [*F*(1, 4) = 26.002, *p* = 0.007, η^2^ = 0.867, Figures 4E,F], with significantly higher Aβ_42_ antigen-positive area in the APP/PS1 mice compared to C57BL/6 J mice. Although the exercise main effect was not significant, LSD simple test under the interaction effect showed a marginally significant exercise simple effect for the AD-E group [*F*(1, 4) = 5.764 *p* = 0.074, η^2^ = 0.592, Figures 4E,F], with a lower Aβ_42_ antigen-positive area compared to the AD-S group.

### 3.4 Reduced iron storage capacity and elevated heme iron metabolism contributed to enhanced lipid peroxidation in APP/PS1 mice, while lipid peroxidation decreased by 8 weeks of aerobic exercise

**Figure 5:**
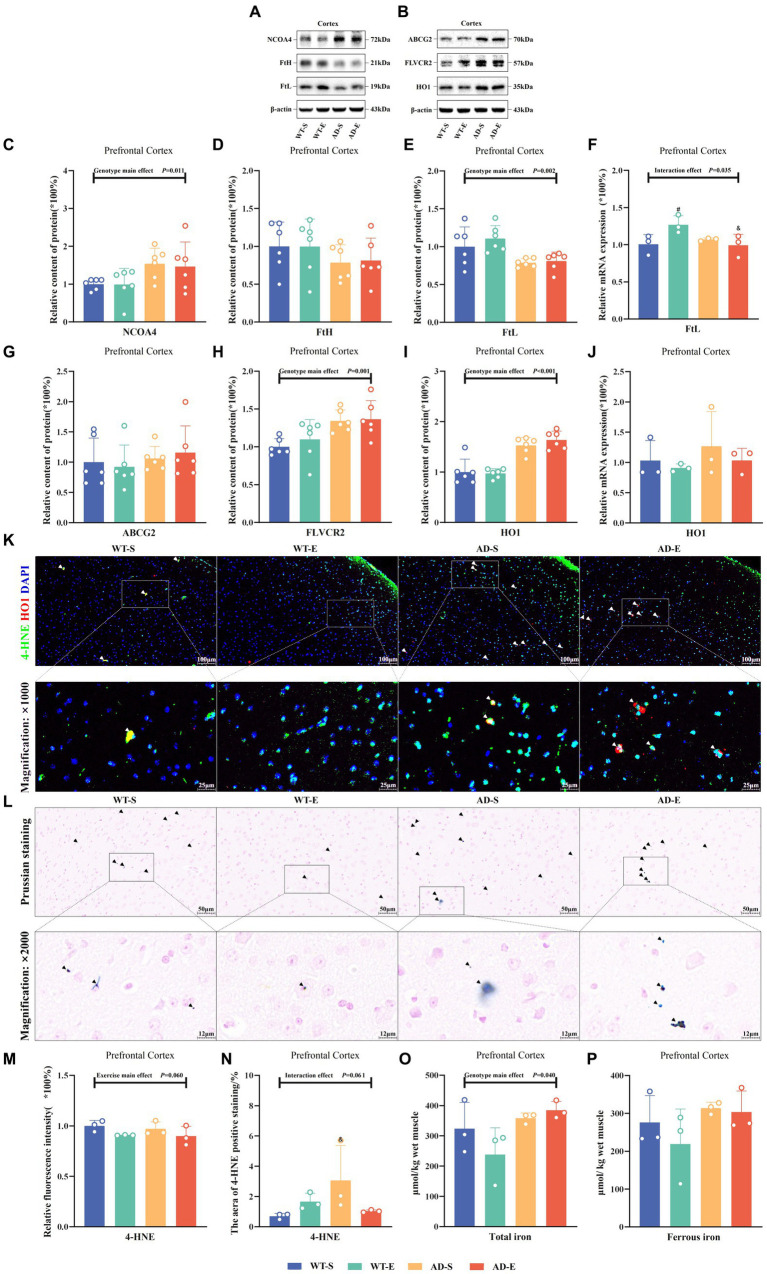
Reduced iron storage capacity and elevated heme iron metabolism associated with enhanced lipid peroxidation in APP/PS1 mice. Western blot protein signals of the PFC in each group **(A)** & **(B)**. Protein expression levels of NCOA4 **(C)**, FtH **(D)**, and FtL **(E)** in the PFC of each group. Relative mRNA expression levels of *FtL* **(F)** in the PFC of each group. Protein expression levels of ABCG2 **(G)**, FLVCR2 **(H)**, and HO1 **(I)** in the PFC of each group, *HO1* mRNA levels **(J)**. IF staining images of the PFC for merged tricolor images with 4-HNE (green), HO1 (red), and DAPI nuclear marker (blue) at 250× (upper) and 1,000× (lower) magnification **(K)**. Prussian blue staining images **(L)** at 500× magnification (upper) and 2000× magnification (lower). Relative fluorescence intensity of 4-HNE **(M)**, and the percentage of image area occupied by fluorescence **(N)**. Total iron **(O)** and ferrous iron levels **(P)** in the PFC of each group as measured by chemical colorimetry. In **(K)**, white arrows indicate the colocalization of 4-HNE and HO1 within cells. In **(L)**, iron-positive areas are indicated by deep blue staining, and black arrows indicate regions positive for Prussian blue staining. In cases of significant interaction effect, “&” indicates significant genotype simple effects between WT-E and AD-E, or between WT-S and AD-S, with *p* ≤ 0.05, while “#” indicates a significant exercise simple effect between WT-S and WT-E with *p* ≤ 0.05.

This study assessed iron storage and ferritin autophagy by measuring the relative protein expression of nuclear receptor coactivator 4 (NCOA4), heavy-chain ferritin (FtH), and light-chain ferritin (FtL), as well as the mRNA levels *FtL*, in the PFC tissues of each group of mice. Western blot protein signals of the PFC in each group are shown in Figures 5A,B. In the PFC, Western blot results showed a significant genotype main effect for NCOA4 expression [*F*(1, 20) = 7.808, *p* = 0.011, η^2^ = 0.281, Figure 5C], as well as significant genotype main effect FtL [*F*(1, 20) = 13.434, *p* = 0.002, η^2^ = 0.402, Figure 5E] expression. Regardless of aerobic exercise intervention, NCOA4 expression in APP/PS1 mice was significantly higher than in C57BL/6 J mice in the PFC, while FtL was lower in APP/PS1 mice than in C57BL/6 J mice. Although there was a trend towards decreased FtH in APP/PS1 mice, there was no significance on FtH (*p* > 0.050, Figure 5D). Additionally, RT-qPCR results showed a significant interaction effect for *FtL* mRNA levels [*F*(1, 8) = 6.434, *p* = 0.035, η^2^ = 0.446, Figure 5F]. Further LSD simple effect tests revealed a significant exercise simple effect for *FtL* mRNA levels in the WT-E group [*F*(1, 8) = 7.629, *p* = 0.025, η^2^ = 0.488, Figure 5F], with significantly higher *FtL* mRNA levels compared to the WT-S group. Moreover, the AD-E group showed a significant genotype simple effect for *FtL* mRNA levels [*F*(1, 8) = 8.460, *p* = 0.020, η^2^ = 0.514, Figure 5F], with significantly lower *FtL* mRNA levels compared to the WT-E group.

To evaluate the influence of heme metabolism and hemoglobin degradation on iron uptake, this study measured the protein expression of ATP-binding cassette subfamily G member 2 (ABCG2), FLVCR2, HO1, and *HO1* mRNA in the PFC. Additionally, the study employed IF double-labeling technique to assess the expression distribution and co-localization of 4-HNE and HO1 in the PFC. Results showed no any significant effect on ABCG2 (*p* > 0.05, Figure 5G). However it had significant main genotype effects on FLVCR2 [*F*(1, 20) = 13.640, *p* = 0.001, η^2^ = 0.405, Figure 5H] and HO1 [*F*(1, 20) = 66.566, *p* < 0.001, η^2^ = 0.769, Figure 5I]. Although there was a trend towards increased *HO1* mRNA levels in APP/PS1 mice, result indicated no significant main effects or interaction effects on *HO1* mRNA levels (*p* > 0.050, Figure 5J). Using Image J software to analyze the fluorescence quantification of 4-HNE immunofluorescence staining, marginal significance was observed for the exercise main effect on the relative fluorescence intensity of 4-HNE [*F*(1, 8) = 4.771, *p* = 0.060, η^2^ = 0.374, Figure 5M] and for the interaction effect on the area of 4-HNE positive staining [*F*(1, 8) = 4.746, *p* = 0.061, η^2^ = 0.372, Figure 5N]. Further LSD simple effect tests revealed a genotype simple effect, with the area of 4-HNE positive staining higher in the AD-S group than the WT-S group [*F*(1, 8) = 5.913, *p* = 0.041, η^2^ = 0.425, Figure 5N]. Additionally, a marginal significance was observed for the exercise simple effect, with the area of 4-HNE positive staining lower in the AD-E group than the AD-S group [*F*(1, 8) = 4.374, *p* = 0.070, η^2^ = 0.353, Figure 5N]. Furthermore, co-localization of 4-HNE and HO1 was observed in PFC of the WT-S, AD-S, and AD-E groups (white arrows in Figure 5K).

This study utilized Prussian blue staining to assess the distribution of iron in the PFC of each group of mice and quantified the levels of total iron and ferrous iron in the PFC using a chemical colorimetry method. Prussian blue staining results indicated the presence of iron ion-positive areas in the brains of all groups, with a broader distribution of iron ion-positive staining observed in the brains of APP/PS1 mice (Figure 5L, black arrows). The quantitative results of the chemical colorimetry method showed a significant genotype main effect on the total iron level in the PFC [*F*(1, 8) = 5.964, *p* = 0.040, η^2^ = 0.427, Figure 5O]. In the APP/PS1 group, the total iron level in the PFC was significantly higher than that in the C57BL/6 J group. Regarding cortical ferrous iron detection, although a similar trend was observed, there was no significant genotype main effect and other effects (*p* > 0.050, Figure 5P).

### 3.5 8 weeks of aerobic exercise activates the Xc^−^/GPx4 pathway enhancing lipid antioxidant capacity in PFC

**Figure 6:**
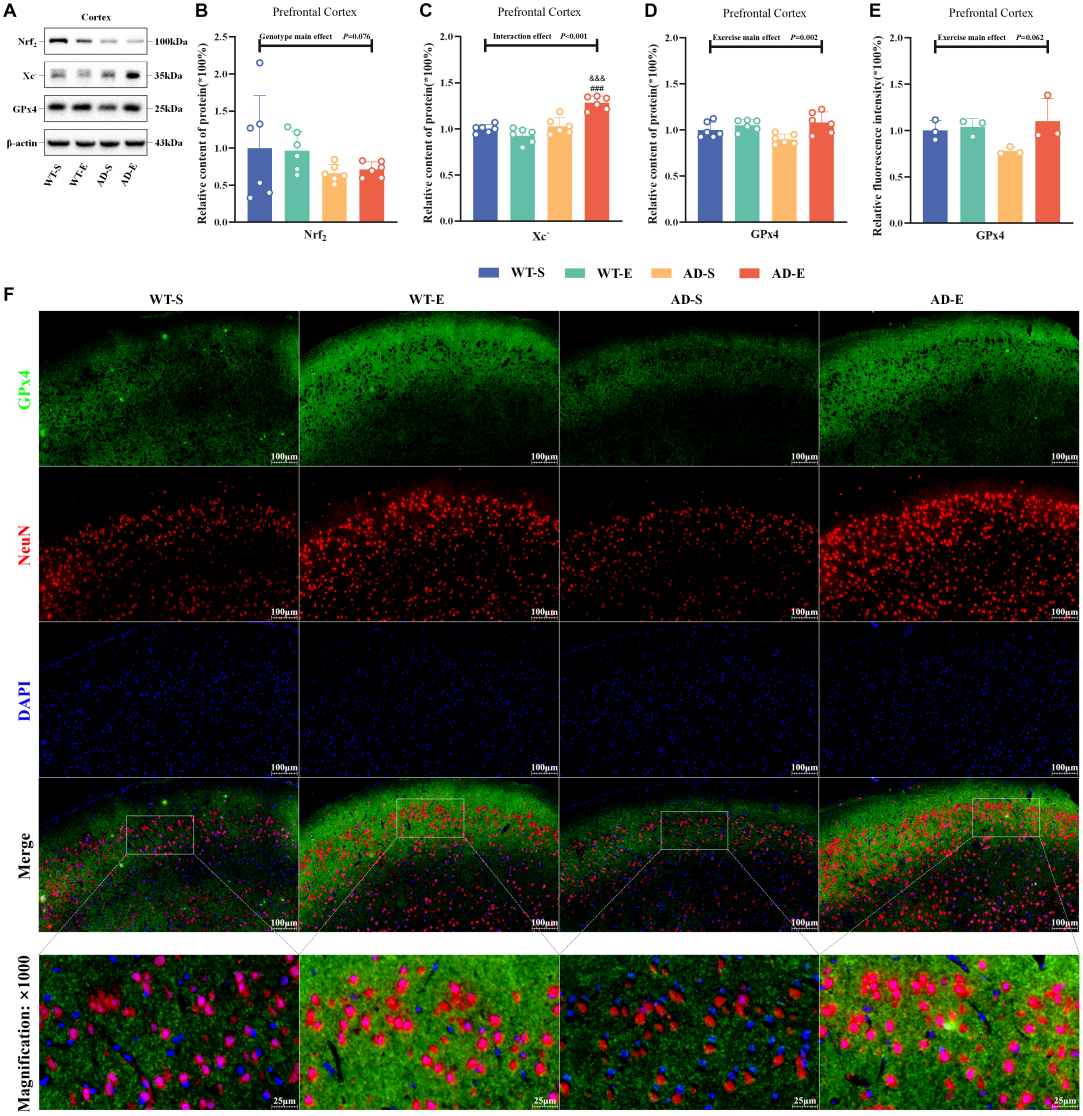
8 weeks of aerobic exercise intervention upregulates the Xc^−^/GPx4 Pathway enhancing lipid antioxidant capacity in PFC. Western blot protein signal images of the PFC in each group **(A)**. Protein expression levels of Nrf_2_ **(B)**, Xc^−^ **(C)**, GPx4 **(D)**, and relative fluorescence intensity of 4-HNE **(E)** in the PFC of each group. IF staining images of the PFC at 250× magnification for GPx4 (green), NeuN (red), and DAPI nuclear marker (blue), with merged tricolor images at 250× (upper) and 1,000× (lower) magnification **(F)**. In cases of significant interaction effect, “&&&” indicates a significant genotype simple effect between AD-E and WT-E with *p* ≤ 0.001, and “###” indicates a significant exercise simple effect between AD-S and AD-E with *p* ≤ 0.001.

To assess the impact of aerobic exercise and genotypes on the lipid antioxidant capacity of the ferroptosis pathway, this study examined the protein expression of Nrf_2_, System Xc^−^ (Xc^−^), and glutathione peroxidase 4 (GPx4) in the PFC. Western blot protein signals of the PFC in each group are shown in Figure 6A. The WB results indicated marginal significance in the main genotype effect of Nrf_2_, with APP/PS1 mice lower than C57BL/6 J mice [*F*(1, 20) = 3.493, *p* = 0.076, η^2^ = 0.149, Figure 6B]. However, there was a significant interaction effect for Xc^−^ [*F*(1, 20) = 28.654, *p* < 0.001, η^2^ = 0.589, Figure 6C], and exercise main effect for GPx4 [*F*(1, 20) = 11.972, *p* = 0.002, η^2^ = 0.374, Figure 6D]. Further LSD simple effect tests revealed a significant genotype simple effect for Xc^−^ expression in AD-E compared to WT-E [*F*(1, 20) = 68.360, *p* < 0.001, η^2^ = 0.774, Figure 6C]. Additionally, compared to AD-S, there was a significant exercise simple effect for Xc^−^ expression in AD-E [*F*(1, 20) = 34.259, *p* < 0.001, η^2^ = 0.631, Figure 6C]. Xc^−^ expression in the AD-E group was significantly higher than that in the AD-S and WT-E groups. The green channel of GPx4 (Figure 6F) was isolated using Image J software, and the fluorescence quantification results of GPx4 immunofluorescence staining were analyzed by selecting a threshold range of 35 to 255, which demonstrated that marginal significance was observed for the exercise main effect on the relative fluorescence intensity of GPx4 [*F*(1, 8) = 4.713, *p* = 0.062, η^2^ = 0.371, Figure 6E], with exercise groups showing higher levels compared to APP/PS1 counterparts.

## 4 Discussion

Exercise is a complex endogenous stress response that can have multifaceted effects on various organs, including the brain. In the treatment and delay of AD, long-term aerobic exercise is widely utilized in clinical practice as an effective intervention to slow the progression of AD. Numerous studies have demonstrated that exercise can delay the onset of AD through a series of biological mechanisms. These include reducing Aβ deposition (Adlard et al., 2005), inhibiting abnormal Tau protein phosphorylation (Ohia-Nwoko et al., 2014), increasing the expression of brain-derived neurotrophic factors (Choi et al., 2018), decreasing neuroinflammation (Nichol et al., 2008), and enhancing neuroplasticity (Wakhloo et al., 2020). Furthermore, in models of healthy individuals, appropriate exercise can also mitigate normal cognitive decline through mechanisms such as increasing cerebral blood flow (Ainslie et al., 2008) and inducing functional hypoxia (Wakhloo et al., 2020). The mechanisms by which exercise delays the onset of AD have not yet been fully elucidated. This study explored the effects of long-term aerobic exercise on iron metabolism and ferroptosis in the prefrontal cortex, thereby providing a perspective on how exercise may mitigate AD.

### 4.1 Improvement of learning and spatial working memory in APP/PS1 mice by 8 weeks of aerobic exercise

Spatial cognition and memory impairment are typical clinical symptoms of AD. The MWM and Y-maze are commonly used to assess spatial working memory and spatial recognition memory in mice or rats. The Y-maze spontaneous alternation task is a method for evaluating the ability of spatial working memory in animal models, with animals demonstrating strong memory typically showing an increase in spontaneous alternation rate and a higher tendency to explore novel arms (Kraeuter et al., 2019). The results of the Y-maze spontaneous alternation task showed that compared to C57BL/6 J mice, APP/PS1 mice had significantly decreased latency to find the novel arm, meanwhile the spontaneous alternation rate in the AD-S group was significantly lower than that in the WT-S group, indicating impaired learning and memory abilities in APP/PS1 mice, leading to prolonged time to find the novel arm and decreased alternation between arms. Conversely, various indices of the novel arm showed significant main effects of exercise, with 8 weeks of aerobic exercise increasing spontaneous alternation counts, activity time in the novel arm, and entries into the novel arm for both C57BL/6 J and APP/PS1 mice, indicating a positive improvement in spatial cognition and learning and memory abilities due to aerobic exercise.

Additionally, in this study, the MWM was further used to assess memory and learning abilities in mice. In the MWM test, experimental animals were trained to find a submerged platform hidden in the pool to assess their spatial cognition and memory abilities. Compared to C57BL/6 J mice, APP/PS1 mice showed longer latency, more errors, and fewer platform crossings, reflecting impaired learning and memory abilities. Previous studies have shown that 8 weeks of aerobic exercise intervention can shorten the latency and increase the number of platform crossings in APP/PS1 mice (Cui et al., 2023). Other studies have also shown that 8 weeks of treadmill exercise in APP-C105 mice reduced the latency and distance in the MWM test, increased the number of platform crossings, and increased the activity time in the quadrant where the platform located (Choi et al., 2021). Consistent with these findings, our study also found that the activity time in the platform quadrant was lower in APP/PS1 mice compared to C57BL/6 J mice, while the escape latency during the learning period was higher in APP/PS1 mice. This indicates impaired learning and memory abilities in APP/PS1 mice. However, 8-weeks aerobic exercise could shorten the escape latency during the learning and testing periods, significantly increase the number of platform crossings. This indicates that the results of the MWM test are consistent with those of the Y-maze test, which indicate 8-weeks aerobic exercise can improve spatial cognition and learning and memory abilities in both C57BL/6 J and APP/PS1 mice.

### 4.2 Increased Aβ_42_ deposition in APP/PS1 mice, but reduced by 8 weeks of aerobic exercise

Aβ depositing in the brain is a primary component of amyloid plaques, a hallmark pathological feature of AD (Hardy and Selkoe, 2002). Additionally, substantial iron deposition has been detected in amyloid plaques (Cheignon et al., 2018). Aβ is a normal byproduct of cellular metabolism, originating from APP (Chen et al., 2017). To be specific, APP has two degradation pathways: In the non-amyloidogenic pathway, APP is first cleaved by α-secretase to generate CTF83 and soluble APPα, followed by cleavage of CTF83 by γ-secretase to produce soluble P3 peptide and the intracellular domain of APP. In the amyloidogenic pathway, APP is initially cleaved by β-secretase to generate CTF99 and soluble APPβ, with subsequent cleavage of CTF99 by γ-secretase resulting in insoluble Aβ_40_ or Aβ_42_ and APP intracellular domain (AICD) (Cheignon et al., 2018). If Aβ_40_ or Aβ_42_ is not promptly degraded or transported, it can interact with metal ions such as iron, copper, and zinc in the brain to form Aβ metal ion oligomers, leading to the formation of Aβ fibrils and subsequent accumulation of Aβ deposition (Cheignon et al., 2018). Compared to the less toxic Aβ_40_, Aβ_42_ is more hydrophobic and prone to aggregation, considered a more critical factor in AD development; among the two, Aβ_42_ is more closely associated with AD pathology (Xiao et al., 2015). In previous studies, Xia et al. (2019) found that aerobic exercise for 3 months reduced Aβ deposition area in the hippocampal tissue of APP/PS1 mice using IHC staining, with ELISA results indicating decreased expression of Aβ_42_ in the hippocampal tissue of APP/PS1 mice following aerobic exercise. Similar findings were reported by Khodadadi et al. (2018). In the cerebral cortex, Belaya et al. (2021) found that voluntary wheel running for 6 months reduced Aβ deposition area in the cerebral cortex of 5 × FAD mice using IF staining. In this study, IHC staining revealed a significant increase in the distribution area of Aβ_42_ monomers in the PFC of APP/PS1 mice; although the main effect of exercise was not significant, LSD simple effect analysis under the interaction effect suggested a marginal significant effect of 8 weeks of aerobic exercise on the downregulation of Aβ_42_ in APP/PS1 mice, consistent with previous studies. These findings indicate that 8 weeks of aerobic exercise intervention can reduce the formation of Aβ_42_ monomers in APP/PS1 mice, which has a positive impact on inhibiting the onset and progression of AD.

### 4.3 The effects of 8-weeks aerobic exercise on iron metabolism and ferroptosis-related pathway in APP/PS1 mice

Iron is an essential component of numerous biomolecules and biological processes. It plays a crucial role in oxygen transport and storage, electron transfer, neurotransmitter synthesis and so on. Despite its importance in normal physiological processes, disruptions in iron homeostasis or iron metabolism are associated with various diseases (González-Domínguez et al., 2014). As early as the 1960s, studies reported significant iron accumulation in the brains of AD patients (Hallgren and Sourander, 1960), particularly in the hippocampus and frontal cortex (Yan and Zhang, 2020; van Duijn et al., 2017). Evidence indicates an increase in brain iron levels in APP/PS1 mice from 3 to 8 months, followed by a decrease at 24 months (Svobodová et al., 2019). However, the mechanisms underlying iron dysregulation in the brains of AD patients remain unclear. In neurons and glial cells, a variety of iron metabolism-related proteins regulate iron transport, uptake, storage, export, and redox processes. Abnormal cellular iron metabolism leads to excessive intracellular iron accumulation, inducing iron overload and potentially triggering ferroptosis.

Hemoglobin metabolism in the brain is regulated by ABCG2 and FLVCR2 (Gozzelino, 2016; Chiabrando et al., 2018; Latunde-Dada et al., 2006; Rajagopal et al., 2008). When hemoglobin is absorbed by neurons via the FLVCR2 receptor, it is degraded by HO1 in conjunction with NADPH and cytochrome P450, releasing free Fe^2+^, carbon monoxide, and biliverdin (Si and Wang, 2020). HO1 is highly sensitive to oxidative stress and inflammatory responses, such as those induced by Aβ, which upregulate *HO1* mRNA expression (Si and Wang, 2020). Biliverdin produced during this process counteracts oxidative stress and inflammation. Elevated HO1 levels have been observed in the brains of AD patients, with significantly increased *HO1* mRNA expression in cortical and vascular brain tissues (Schipper et al., 1995, 2000). Additionally, HO1 is highly expressed in glial cells positive for senile plaques and NFTs, indicating its multifaceted role in neurons (Schipper et al., 2000). HO1 generates neuroprotective biliverdin and removes aberrant Tau proteins in AD pathology (Barone et al., 2014; Choi and Kim, 2022). However, while protecting neurons by degrading heme, HO1 also produces free Fe^2+^, which exacerbates oxidative stress through the Fenton reaction (Ward et al., 2014). In previous literature, only one study has reported the effect of exercise on heme iron metabolism in AD models (Belaya et al., 2021). They found a significant upregulation of *HO1* mRNA levels in the cortex of 5 × FAD mice, which is consistent with our findings at the HO1 protein expression and transcription levels. Notably, they also found that exercise significantly downregulated *HO1* mRNA levels and suggested that exercise-induced downregulation of HO1 expression could inhibit brain iron overload. However, our study did not observe a similar trend at the protein level, although a similar trend was observed at the transcription level in AD-E mice. The upregulation of HO1 protein and transcription levels in the PFC of APP/PS1 mice observed in this study might be related to a compensatory response under AD pathology. Due to enhanced neuroinflammation and oxidative stress in the AD brain, increased heme intake is necessary to form biliverdin to counteract oxidative stress and inhibit neuroinflammation. However, under AD pathological conditions, the resultant free Fe^2+^ from this process is difficult to manage, leading to enhanced membrane lipid peroxidation and induction of the lipid peroxidation product such as 4-HNE. Thus, cells may require other effective pathways to neutralize the negative effects of Fe^2+^ produced by HO1 degradation. This potential pathway could be the Xc^−^/GPx4 anti-lipid peroxidation pathway that we discovered in this study. Additionally, this study found a significant increase in the protein expression levels of key heme uptake molecule FLVCR2 in the brains of APP/PS1 mice. This might also be a compensatory effect related to elevated HO1 levels, suggesting an increased heme flux in the brain. Increased FLVCR2 expression facilitates substantial heme entry into cells, promoting HO1-mediated release of free Fe^2+^, further intensifying oxidative stress through the Fenton reaction in the PFC. Using double IF labeling, we found colocalization of 4-HNE and HO1 in the PFC of APP/PS1 mice, suggesting that high HO1 expression may induce lipid peroxidation and upregulate 4-HNE of a lipid peroxidation marker. Additionally, the expression of 4-HNE exhibited a marginally significant main effect of exercise, indicating that 8 weeks of aerobic exercise can reduce cellular lipid peroxidation, which is beneficial in preventing subsequent neuronal ferroptosis. The increase of HO1 expression in APP/PS1 mice was consistent with the increase of iron level, which indicate the increase of HO1 expression result the increase in total iron. In this study, we proved the point via chemical colorimetric assays and Prussian blue staining in the PFC. The results indicated a significant increase in total iron levels in the PFC of APP/PS1 mice, with a similar trend observed for ferrous iron levels. Prussian blue staining also revealed similar strain-specific differences in tissue morphology. In previous studies, only two have investigated the impact of exercise on brain iron levels in AD models. Belaya’s study using inductively coupled plasma mass spectrometry found no significant exercise main effect on total iron levels in the cortex of 6-month-old 5 × FAD mice (Belaya et al., 2021). Conversely, Choi’s study using a chemical colorimetric method demonstrated that exercise has profound influence on APP/PS1, which demonstrated that 8-weeks of exercise reduced Fe^2+^, Fe^3+^, and total iron levels in the motor cortex of 24-month-old APP-C105 mice (Choi et al., 2021). Our findings are consistent with Choi’s study. Eight weeks of aerobic exercise reduced iron levels in the PFC of C57BL/6 J mice. The discrepancy between this study and Belaya’s could be attributed to differences in mouse strains and iron measurement methods. Overall, the findings indicate a significant increase in iron levels in the PFC under AD pathological conditions, leading to substantial iron deposition in the frontal cortex. Although a trend towards reduced total iron and ferrous iron levels was observed in the exercised C57BL/6 J mice, no significant exercise main effect was found statistically.

Ferritin is a hollow iron storage protein composed of highly symmetrical FtH and FtL subunits (Arosio et al., 2009). Its primary function is to store excess free iron ions in the body, thereby reducing the toxic effects of free iron ions. However, NCOA4 can bind to ferritin and transport it to lysosomes for autophagy, leading to the release of large amounts of Fe^3+^ into the cytoplasm and increasing sensitivity to ferroptosis (Wang et al., 2022). In neuroinflammation, NCOA4 interacts with ferritin, promoting its autophagic degradation (Wang et al., 2022). The degradation of ferritin further releases a substantial amount of free iron, ultimately triggering lipid peroxidation and ferroptosis. Previous studies have shown that FtH and FtL are significantly elevated in AD model mice, whereas aerobic exercise can reduce their expression (Choi et al., 2021). Conversely, other studies have indicated that voluntary wheel running significantly reduces ferritin mRNA levels in the cortex of 5 × FAD mice, which is consistent with our results regarding *FtL* mRNA levels (Belaya et al., 2021). Through quantitative analysis of FtH and FtL protein expression, we found that FtL protein levels were significantly reduced in the PFC of APP/PS1 mice in this study. Additionally, using RT-qPCR to quantify *FtL* mRNA levels, we found that 8 weeks of aerobic exercise increased *FtL* mRNA expression, while *FtL* mRNA levels were downregulated in the APP/PS1 exercise group. The intergroup transcriptional changes in FtL of this study are consistent with Belaya’s findings (Belaya et al., 2021). The discrepancy between *FtL* mRNA and protein levels suggests that *FtL* may be regulated by factors that enhance FtL protein degradation. The upregulation of NCOA4, a key factor in ferritin autophagy, in APP/PS1 mice may induce the autophagic degradation of FtL. Therefore, the reduction of FtH and FtL in the PFC may be related to AD-induced NCOA4 upregulation. The decreased ferritin in the PFC of APP/PS1 mice cannot accommodate the storage of excess free iron, leading to an increase in free iron ions, which exacerbates the Fenton reaction, promotes the formation of PUFAs peroxides, and increases sensitivity to ferroptosis. The elevated total iron levels in the PFC of APP/PS1 mice of our study also supports this conclusion.

PUFAs are highly expressed and easily peroxidized fatty acids in cell membranes. These fatty acids are activated and re-esterified by long-chain acyl-CoA synthetase 4 and Lys phosphatidylcholine acyltransferase 3, forming lipid-peroxyl radicals that infiltrate the lipid bilayer of organelles or cell membranes. These lipid-peroxyl radicals, through autoxidation or enzymatic processes, form lipid peroxides, thereby inducing negative cascading reactions such as altered membrane fluidity and increased permeability (Kagan et al., 2017). The Xc^−^ system is a cystine/glutamate transporter and constitutes the rate-limiting step in GSH synthesis. The antioxidant function of GSH requires GPx4 as a cofactor to reduce membrane lipid peroxides to non-toxic alcohols, thereby inhibiting the oxidative damage of PUFAs during ferroptosis. Consequently, cystine metabolism dysfunction and GPx4 expression inhibition led to the accumulation of intracellular lipid peroxides, elevating sensitivity to ferroptosis. Simultaneously, GPX4 can inhibit NF-κb/TNF pathway to prevent the occurrence of ferroptosis activated by neuroinflammation (Li et al., 2018). In the prevention of ferroptosis, Nrf_2_, a transcription factor activated in response to cellular oxidative stress and other stimuli, plays a crucial role. The critical downstream targets of the Nrf_2_ pathway are GPx4 and SLC7A11 (Hayes and Dinkova-Kostova, 2014). The SLC7A11 is the functional subunit of the Xc^−^ system. Currently, there is no literature reporting the regulation of ferroptosis-related lipid antioxidant pathways in AD models by exercise. However, a study has shown that continuous treadmill exercise for 14 days, at 30 min per day and 10 meters/min, increased the expression of Nrf_2_, SLC7A11, and GPx4 in an ischemia–reperfusion brain model. Injection of the SLC7A11 inhibitor Erastin reduced the expression of SLC7A11 and GPx4, indicating that 14 days of aerobic exercise can activate the Nrf_2_/SLC7A11/GPx4 pathway, thereby reducing sensitivity to ferroptosis in the ischemia–reperfusion brain model (Liu et al., 2022). Another study reported that 4 weeks of aerobic exercise reversed ferroptosis markers in a traumatic brain injury model, including upregulating the expression of GPx4, Xc^−^, and FPN1 (Chen et al., 2023). These results suggest that aerobic exercise can reduce sensitivity to ferroptosis. Ramsey et al. observed a significant decrease in Nrf_2_ levels in AD cases (Ramsey et al., 2007). Additionally, Wu et al. found that Nrf_2_ expression was significantly reduced in a sporadic AD rat model, while swimming intervention activated Nrf_2_ expression (Wu et al., 2018). In this study, 8 weeks of aerobic exercise did not observe an upregulation of Nrf_2_. But we found that Nrf_2_ expression was reduced in the PFC of APP/PS1 mice, consistent with Ramsey’s previous finding (Ramsey et al., 2007). Theoretically, Nrf_2_ downregulation would induce a decrease in Xc^−^ and GPx4. However, our study found an opposite trend in the expression of Xc^−^ and GPx4 in the PFC, suggesting that aerobic exercise might regulate Xc^−^ and GPx4 expression through pathways independent of Nrf_2_. This study discovered that 8 weeks of aerobic exercise significantly increased Xc^−^ levels in the PFC of APP/PS1 mice and significantly elevated GPx4 levels in the PFC. These findings suggest that aerobic exercise can upregulate Xc^−^ expression through pathways independent of Nrf_2_, enhance the transport of glutathione synthesis precursors, and increase GPx4 expression and utilization of GSH in both APP/PS1 and C57BL/6 J mice. This helps counteract ROS produced by the iron-dependent Fenton reaction, inhibit the peroxidation of PUFAs, and play a positive role in reducing neuronal sensitivity to ferroptosis.

## 5 Conclusion

**Figure 7:**
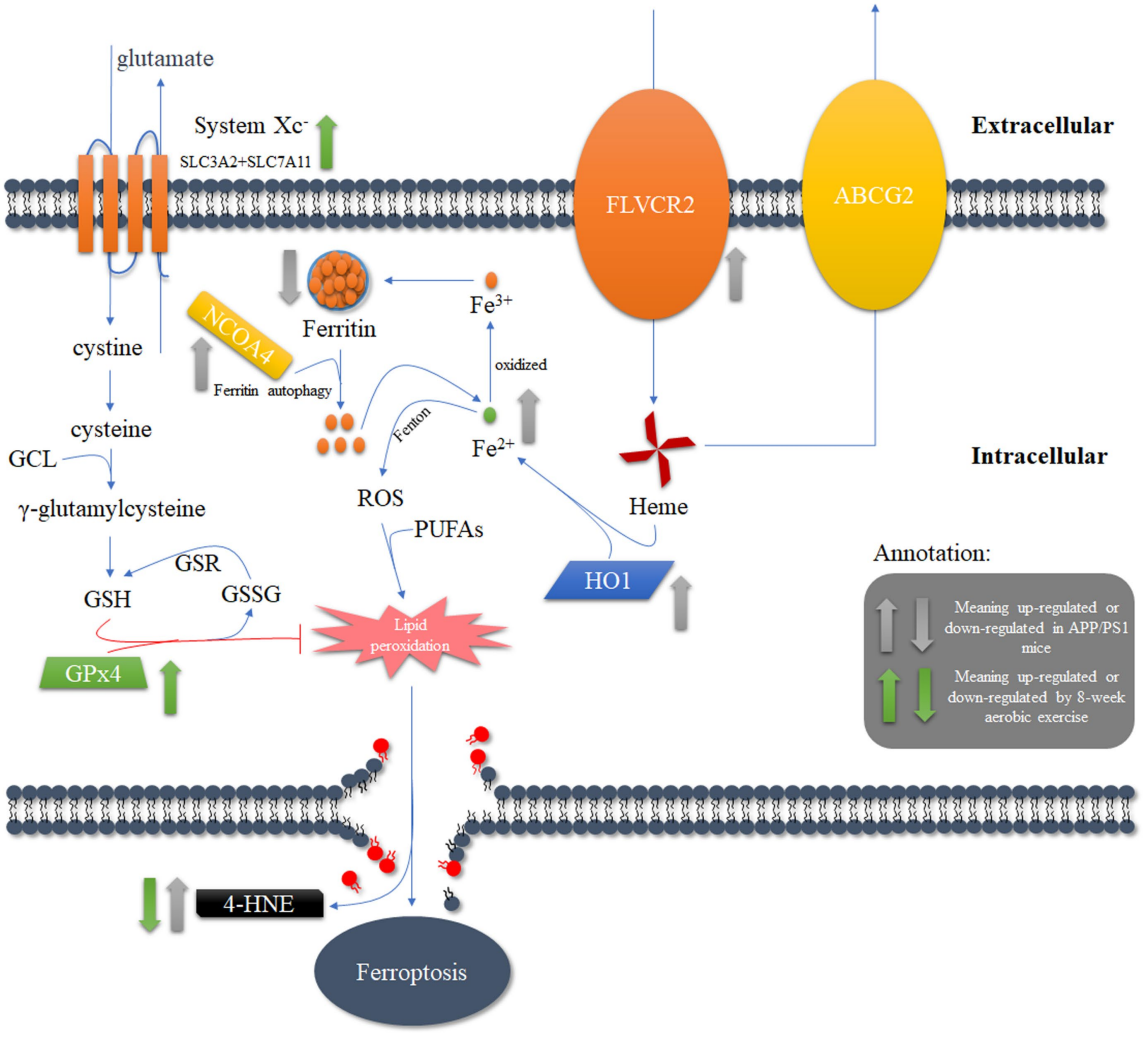
The potential mechanism underlying the reduction of ferroptosis in APP/PS1 mice through the activation of lipid antioxidant pathways after 8 weeks of aerobic exercise intervention.

In summary, the enhanced heme degradation and reduced ferritin levels in the PFC of APP/PS1 mice may exacerbate the oxidative stress effects of Fe^2+^, leading to increased sensitivity to neuronal ferroptosis. However, 8-weeks aerobic exercise may inhibit the lipid peroxidation induced by these processes through the activation of the Xc^−^/GPx4 pathway, thereby reducing the sensitivity of neurons to ferroptosis and positively improving the spatial cognition, learning and memory abilities of APP/PS1 mice (Figure 7).

## Data Availability

The original contributions presented in the study are included in the article/Supplementary material, further inquiries can be directed to the corresponding author.
