# Supplementary figures and images for: 8-weeks aerobic exercise ameliorates cognitive deficit and mitigates ferroptosis triggered by iron overload in the prefrontal cortex of APPSwe/PSEN1dE9 mice through Xc−/GPx4 pathway
Source: Front Neurosci. 2024 Sep 9;18:1453582. doi: 10.3389/fnins.2024.1453582 (PMC11417105; doi:10.3389/fnins.2024.1453582)

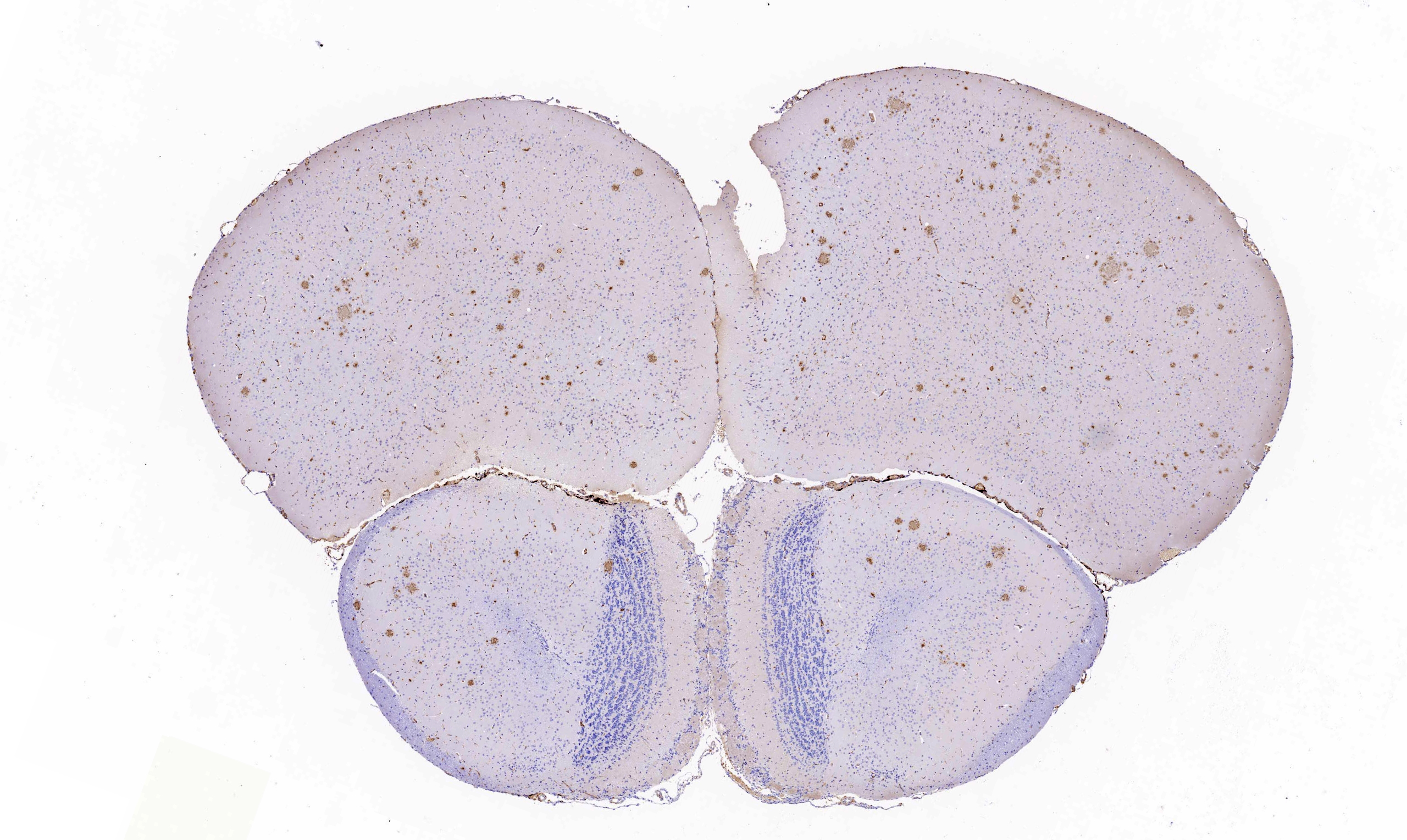

Supplement: Supplementary file 1 [file Presentation_1.ZIP › Abeta42/ADE3─╘ ╟░╢ε Amyloid 4_4.0x.jpg]

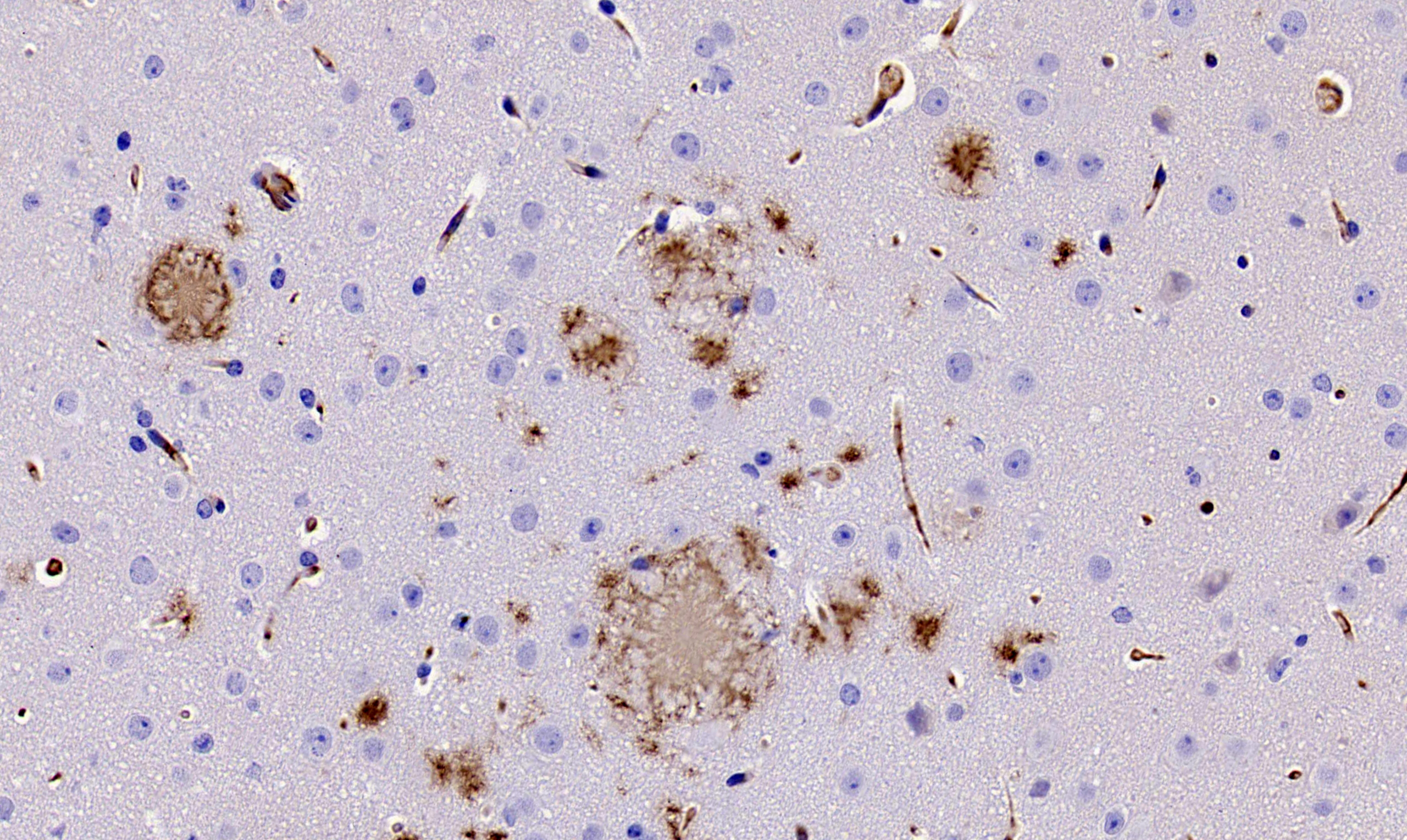

Supplement: Supplementary file 1 [file Presentation_1.ZIP › Abeta42/ADE3─╘ ╟░╢ε Amyloid 4_50.0x.jpg]

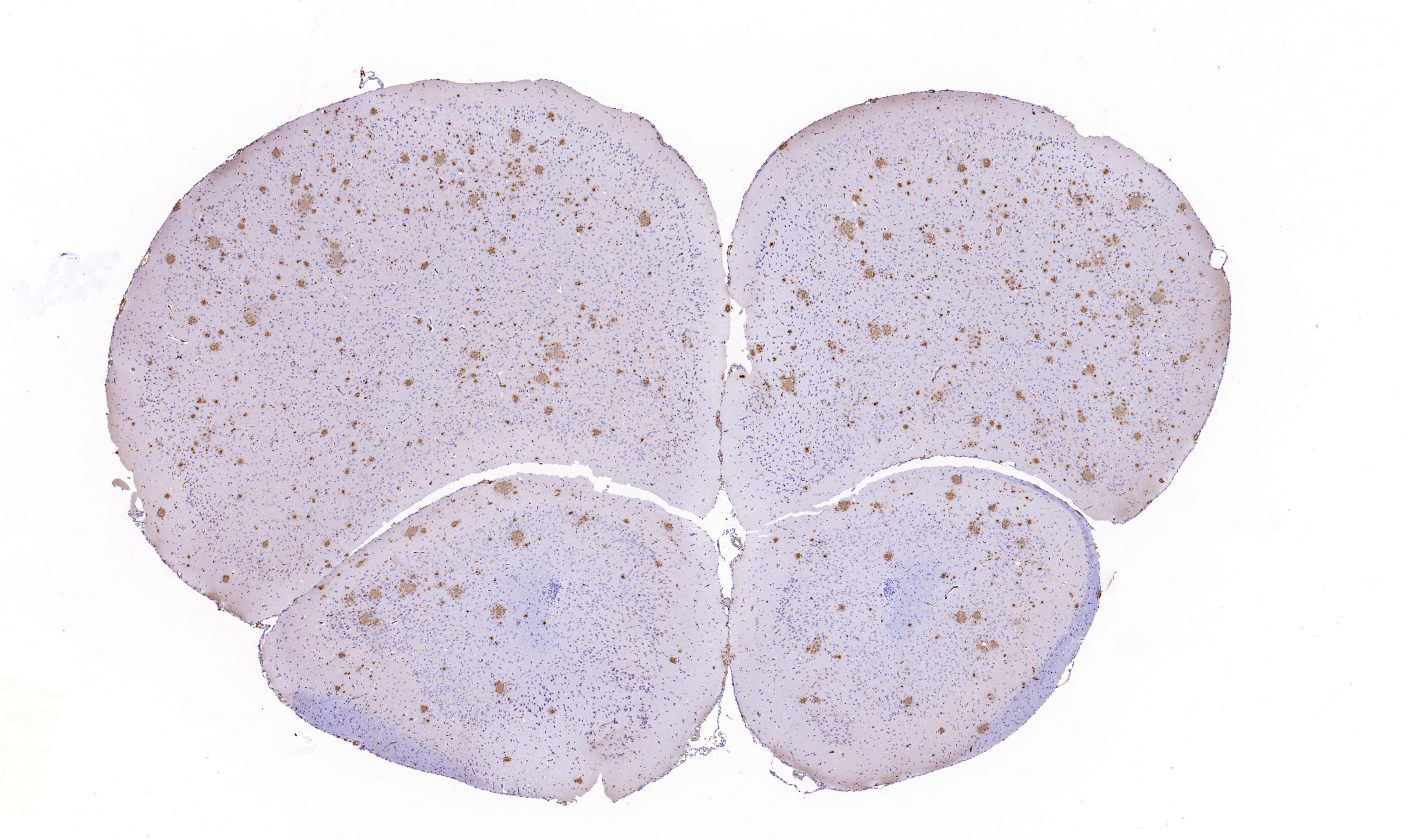

Supplement: Supplementary file 1 [file Presentation_1.ZIP › Abeta42/ADS3─╘ ╟░╢ε Amyloid 4_4.0x.jpg]

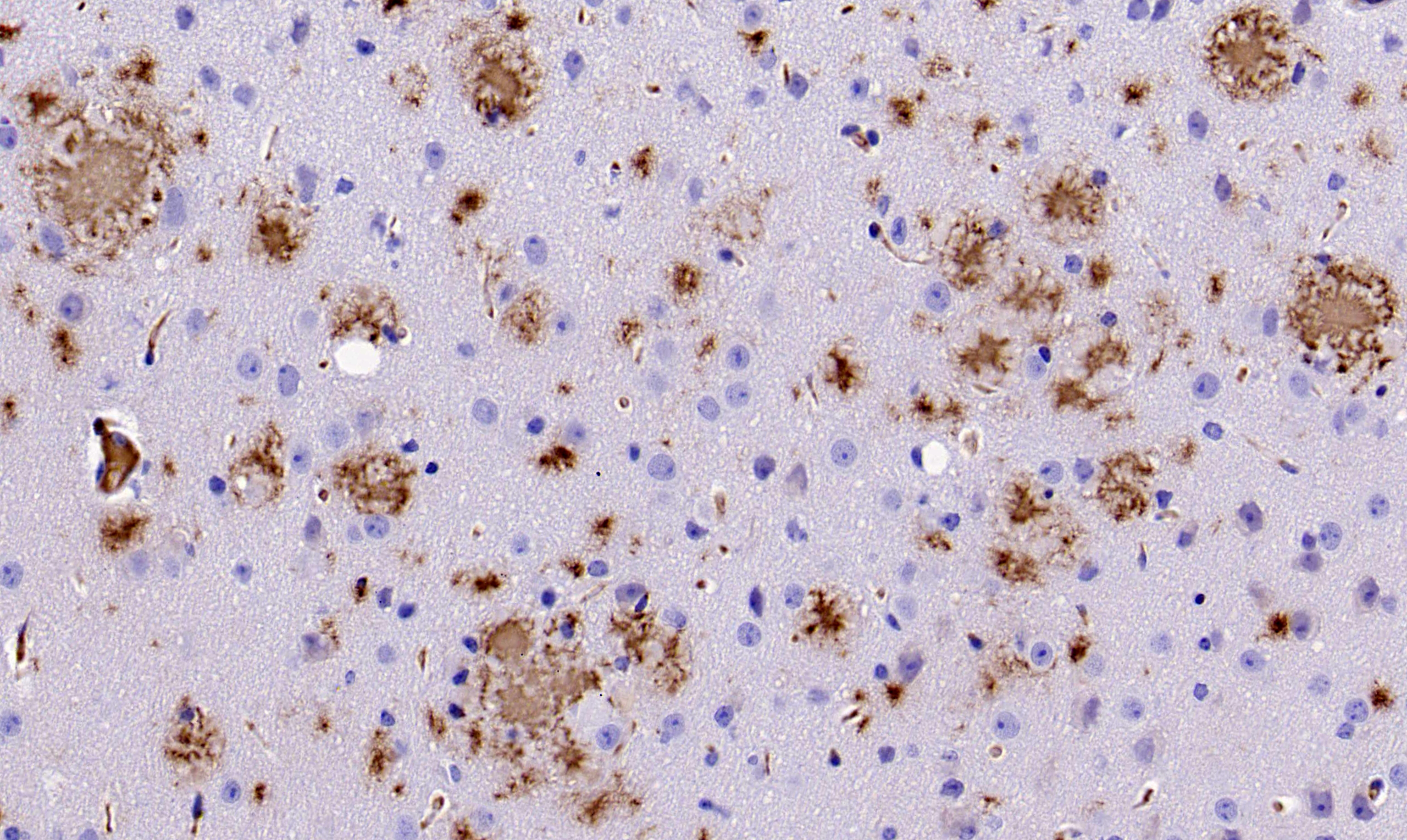

Supplement: Supplementary file 1 [file Presentation_1.ZIP › Abeta42/ADS3─╘ ╟░╢ε Amyloid 4_50.0x.jpg]

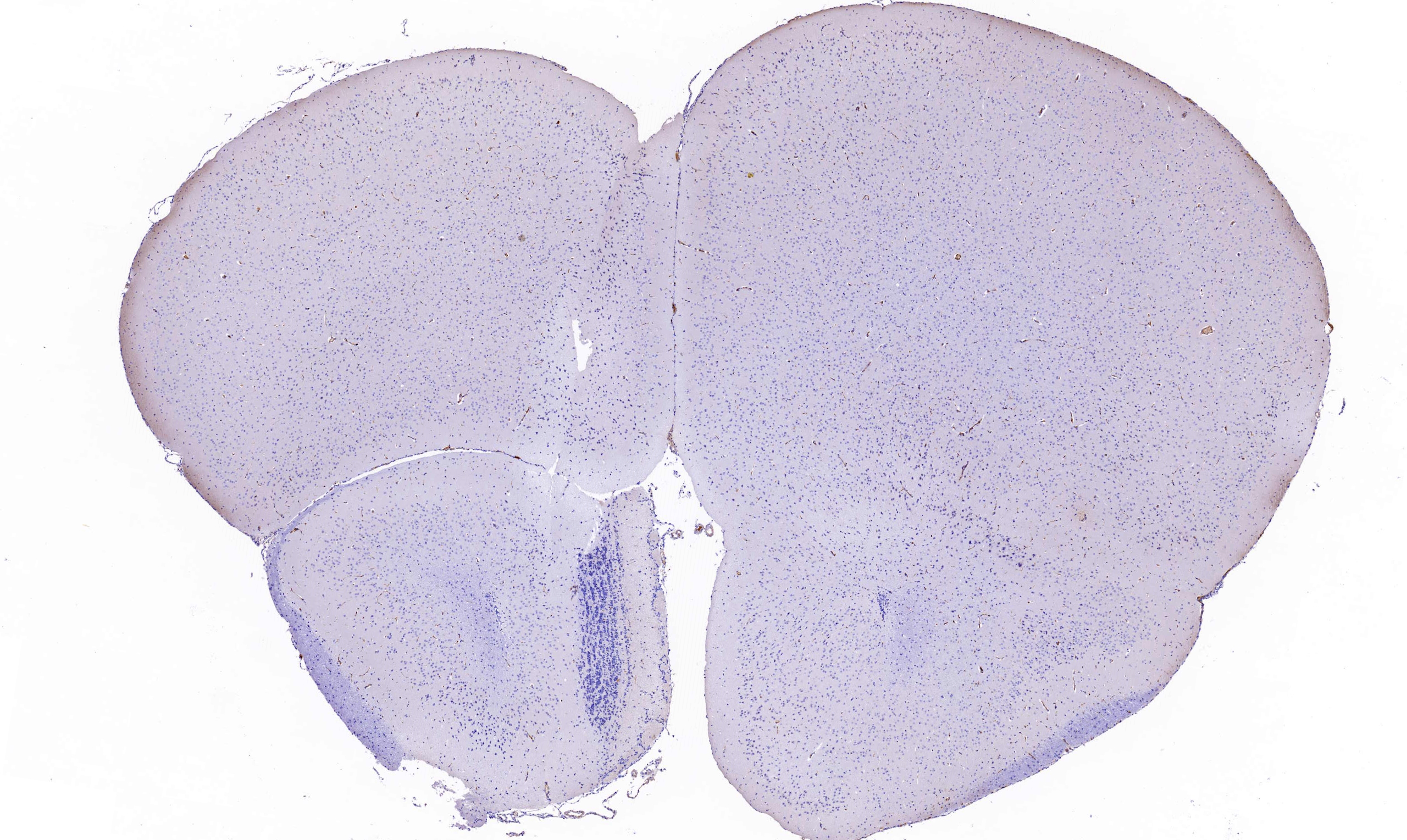

Supplement: Supplementary file 1 [file Presentation_1.ZIP › Abeta42/WTE3─╘ ╟░╢ε Amyloid 4_4.0x.jpg]

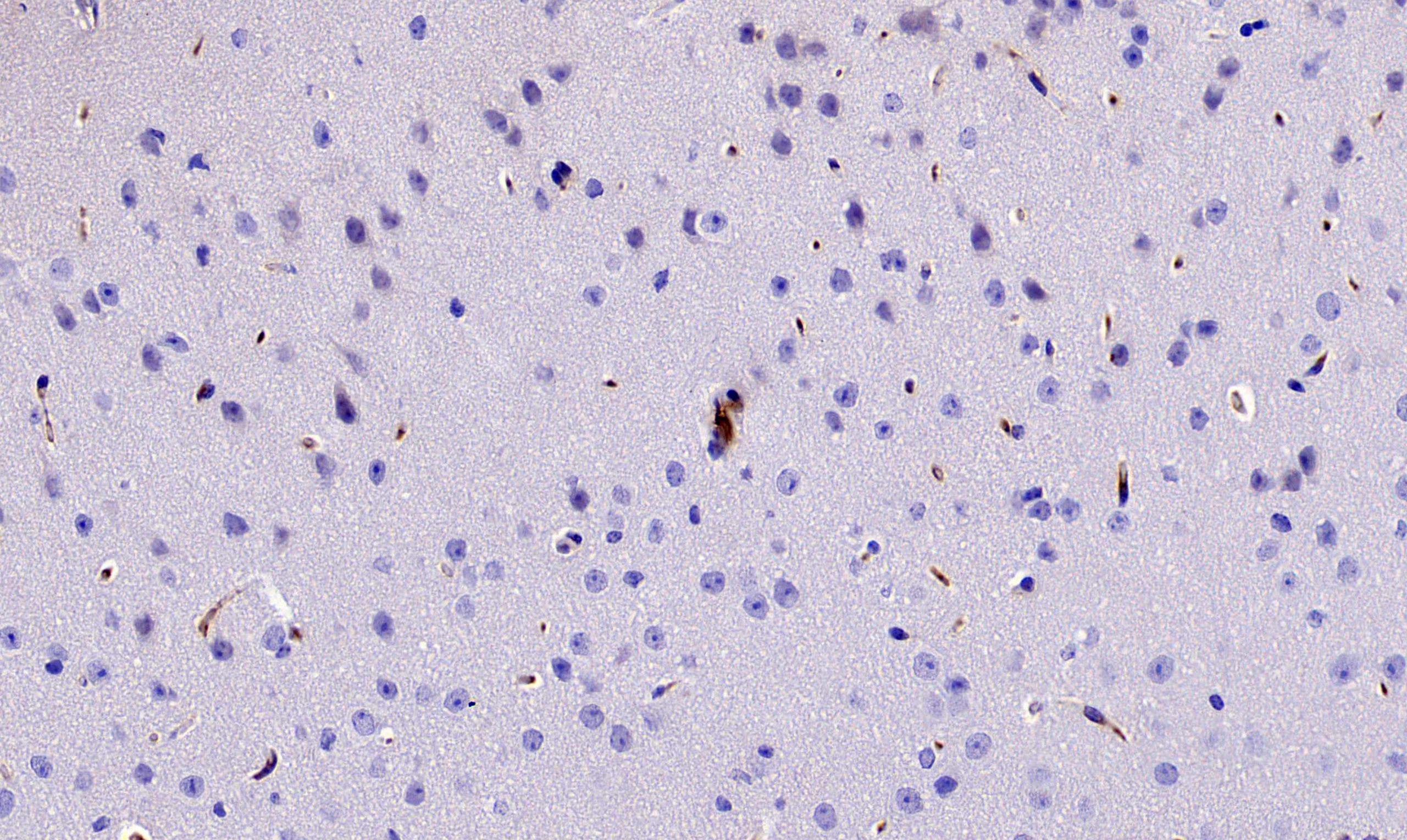

Supplement: Supplementary file 1 [file Presentation_1.ZIP › Abeta42/WTE3─╘ ╟░╢ε Amyloid 4_50.0x.jpg]

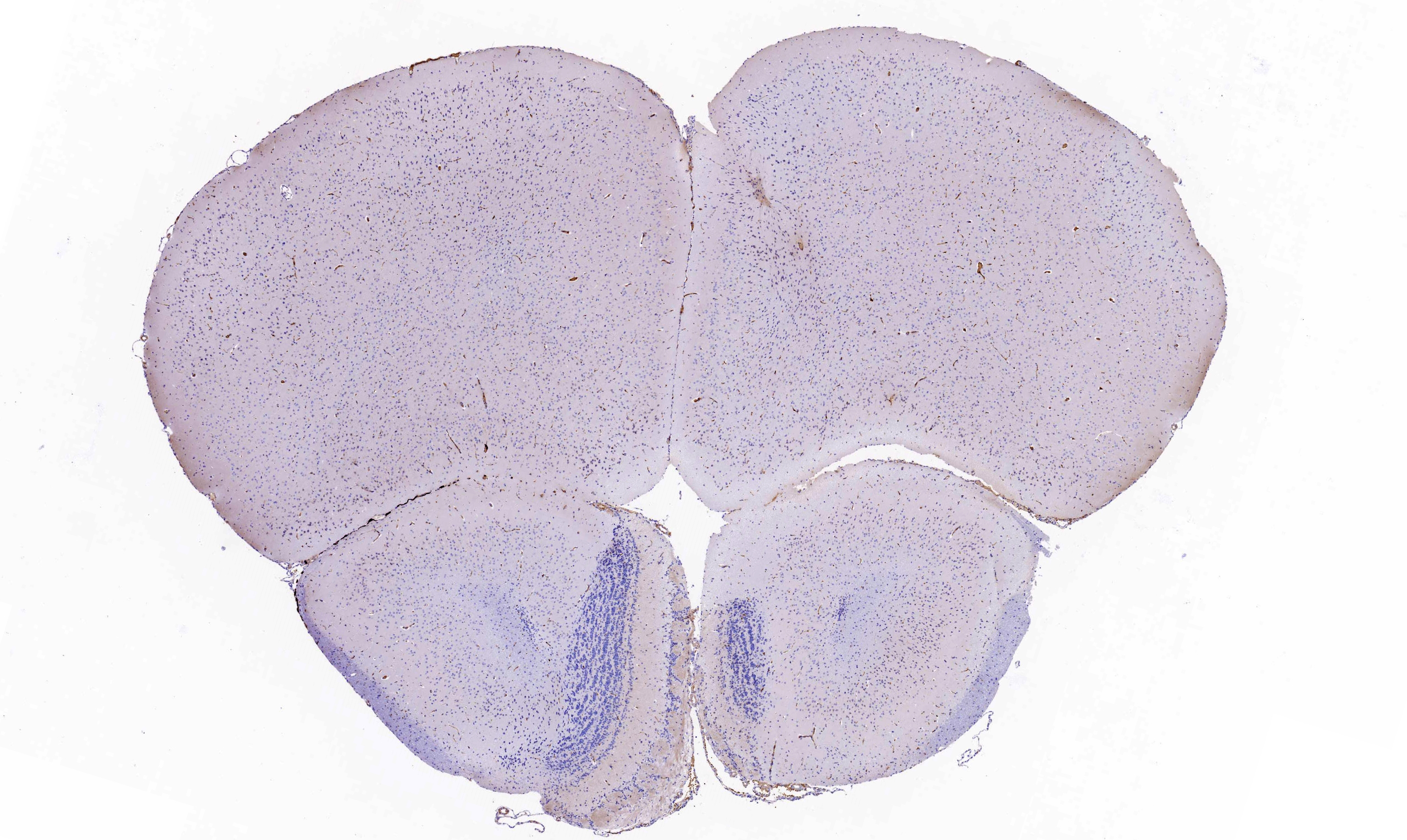

Supplement: Supplementary file 1 [file Presentation_1.ZIP › Abeta42/WTS3─╘ ╟░╢ε Amyloid 4_4.0x.jpg]

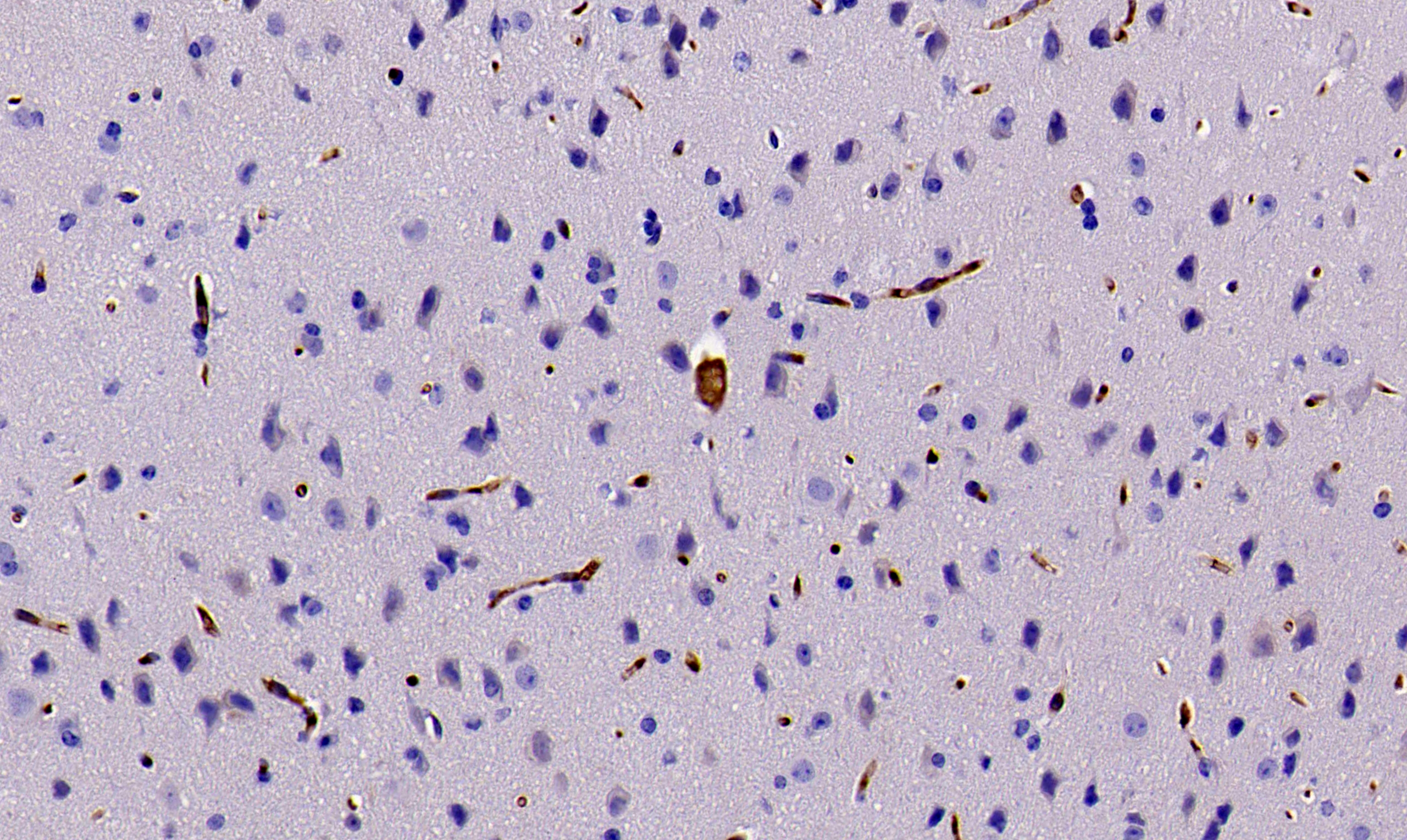

Supplement: Supplementary file 1 [file Presentation_1.ZIP › Abeta42/WTS3─╘ ╟░╢ε Amyloid 4_50.0x.jpg]

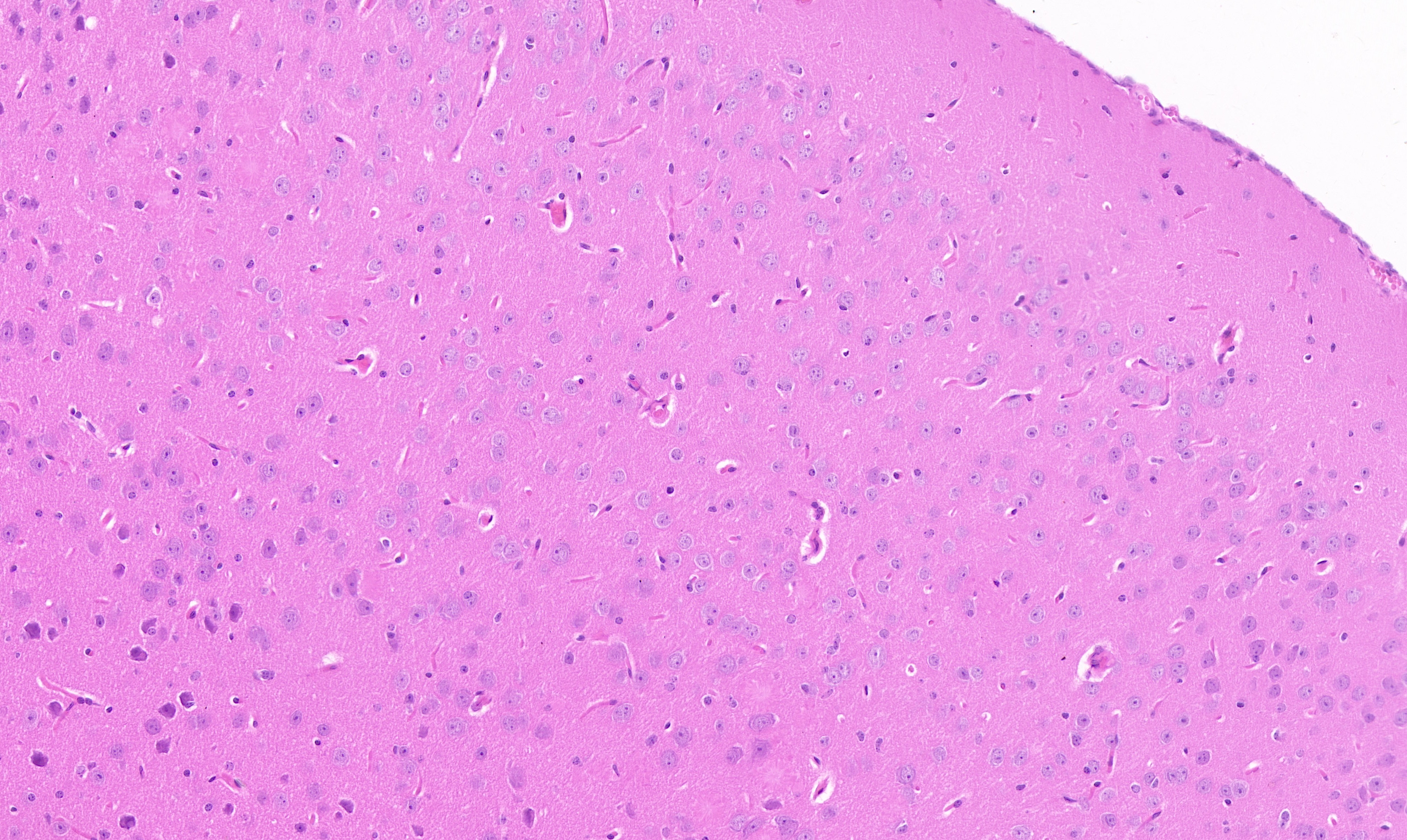

Supplement: Supplementary file 2 [file Presentation_2.ZIP › HE staining/ADE3 ─╘ ╟░╢ε_25.0x.jpg]

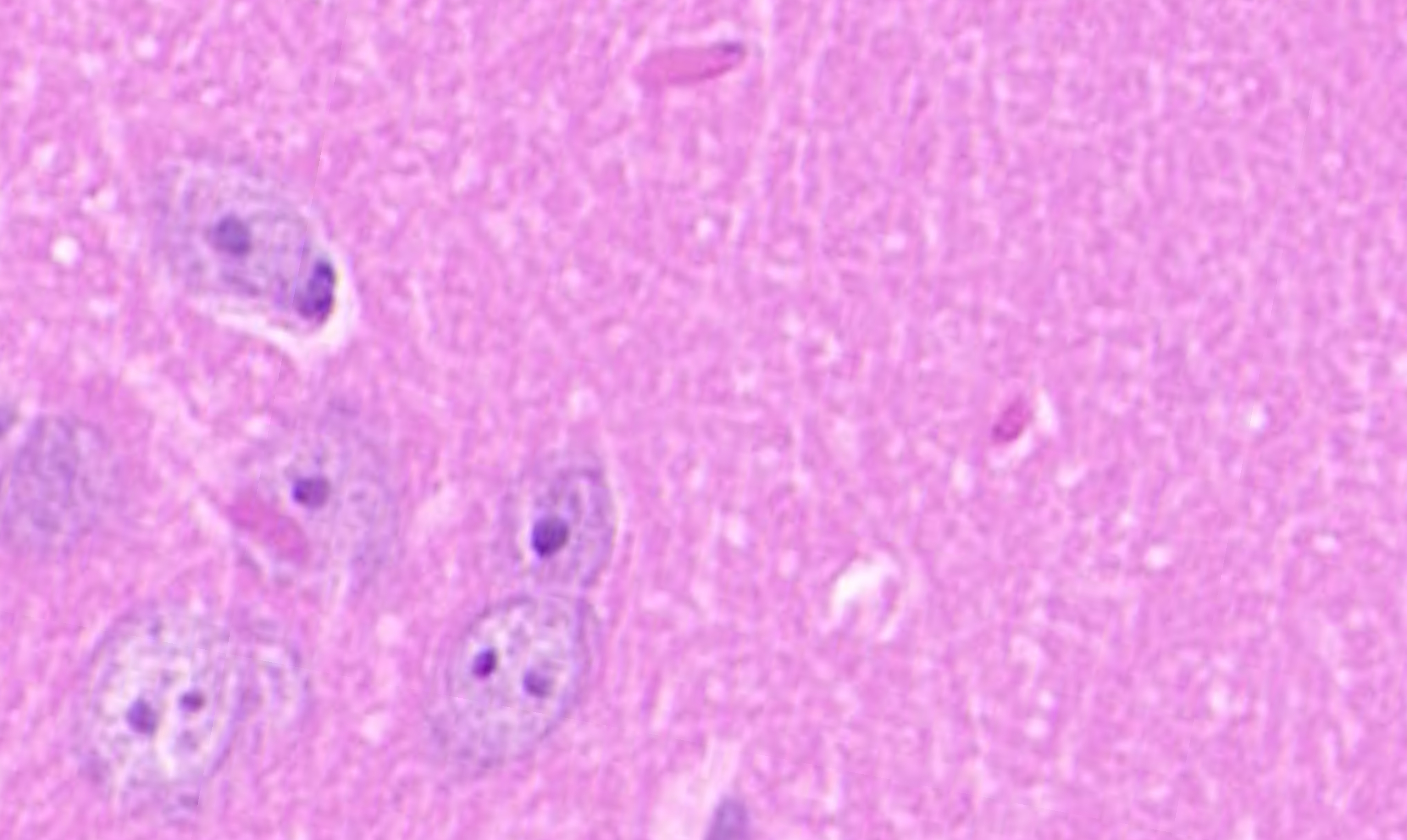

Supplement: Supplementary file 2 [file Presentation_2.ZIP › HE staining/ADE3 ─╘ ╟░╢ε_250.0x.jpg]

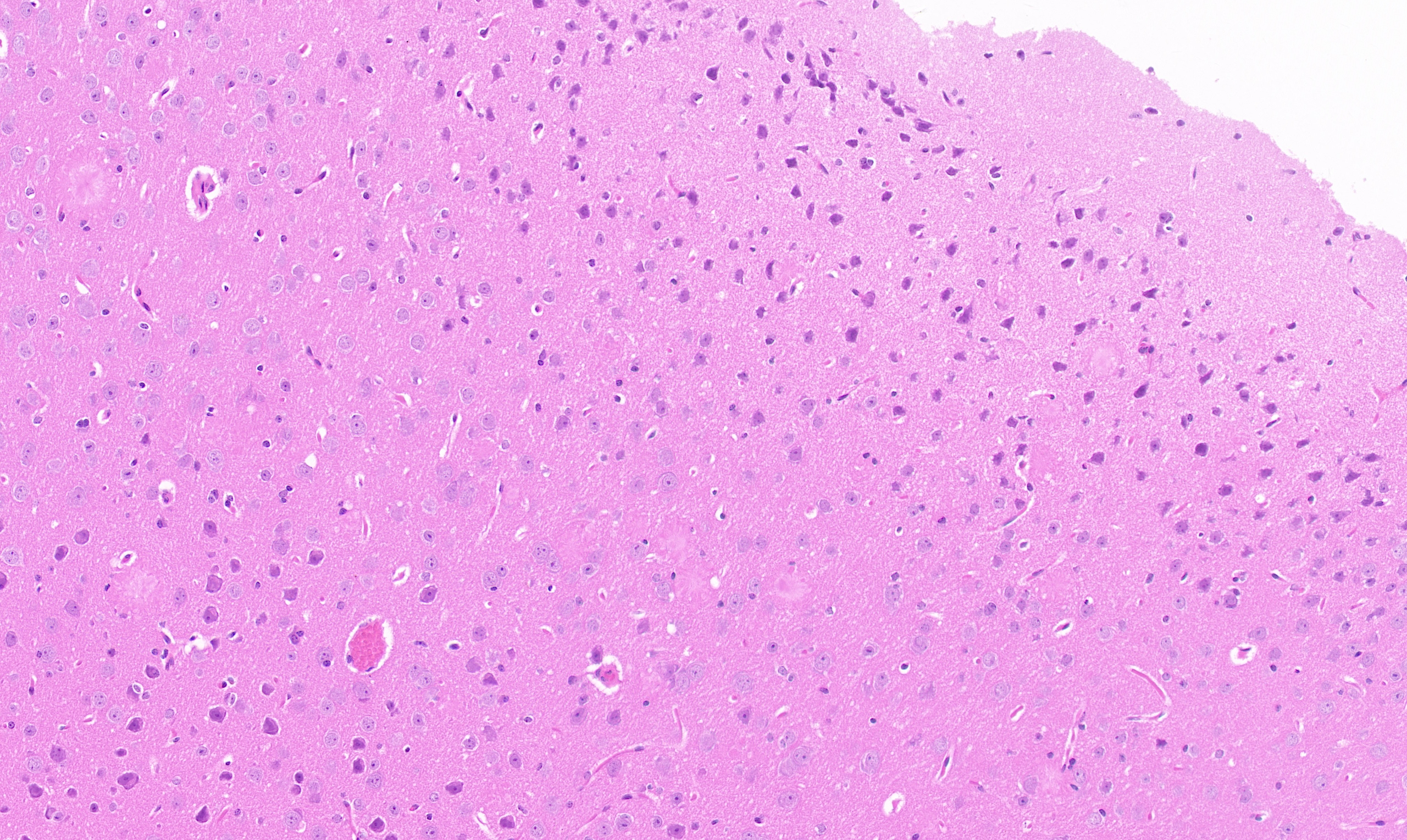

Supplement: Supplementary file 2 [file Presentation_2.ZIP › HE staining/ADS3 ─╘ ╟░╢ε_25.0x.jpg]

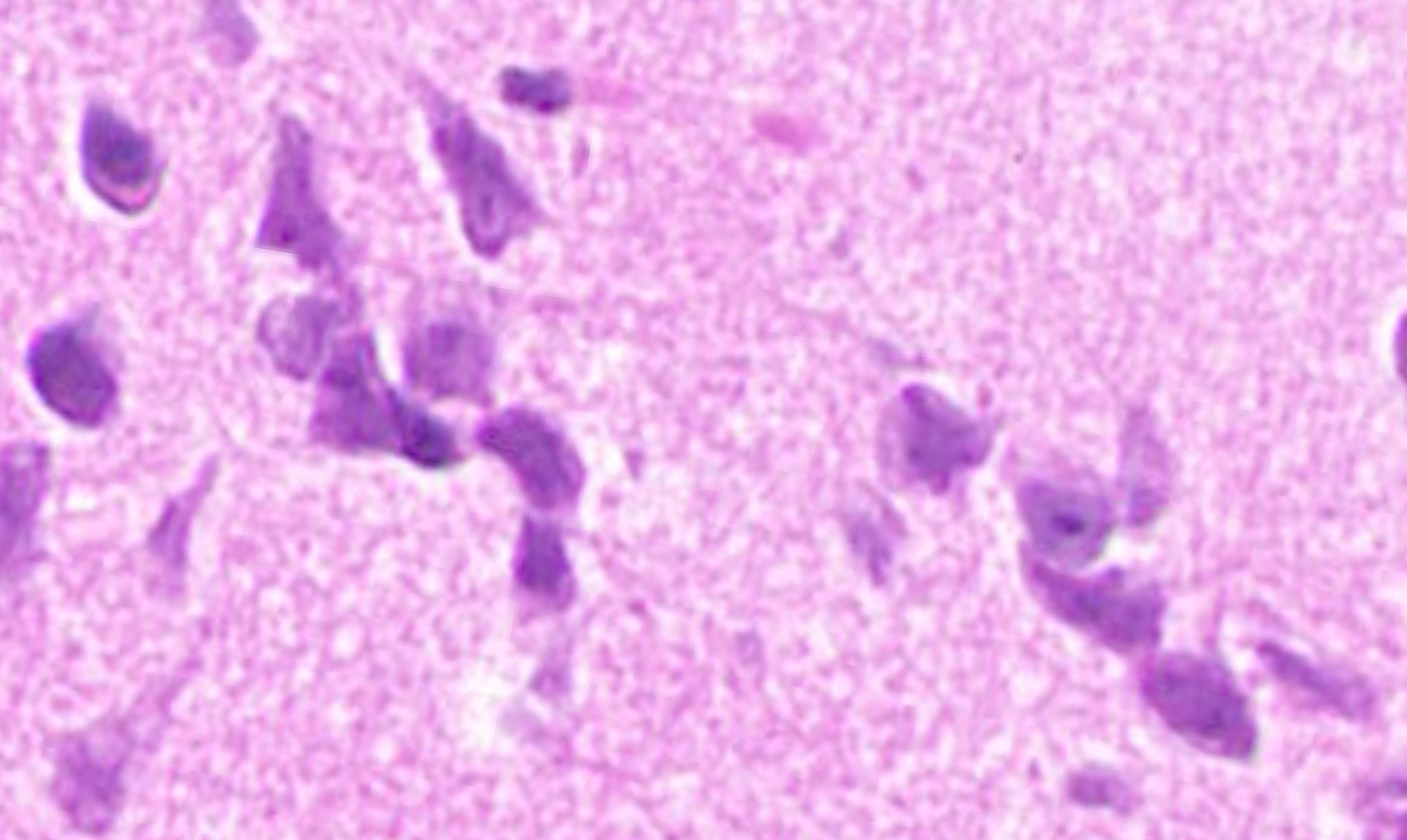

Supplement: Supplementary file 2 [file Presentation_2.ZIP › HE staining/ADS3 ─╘ ╟░╢ε_250.0x.jpg]

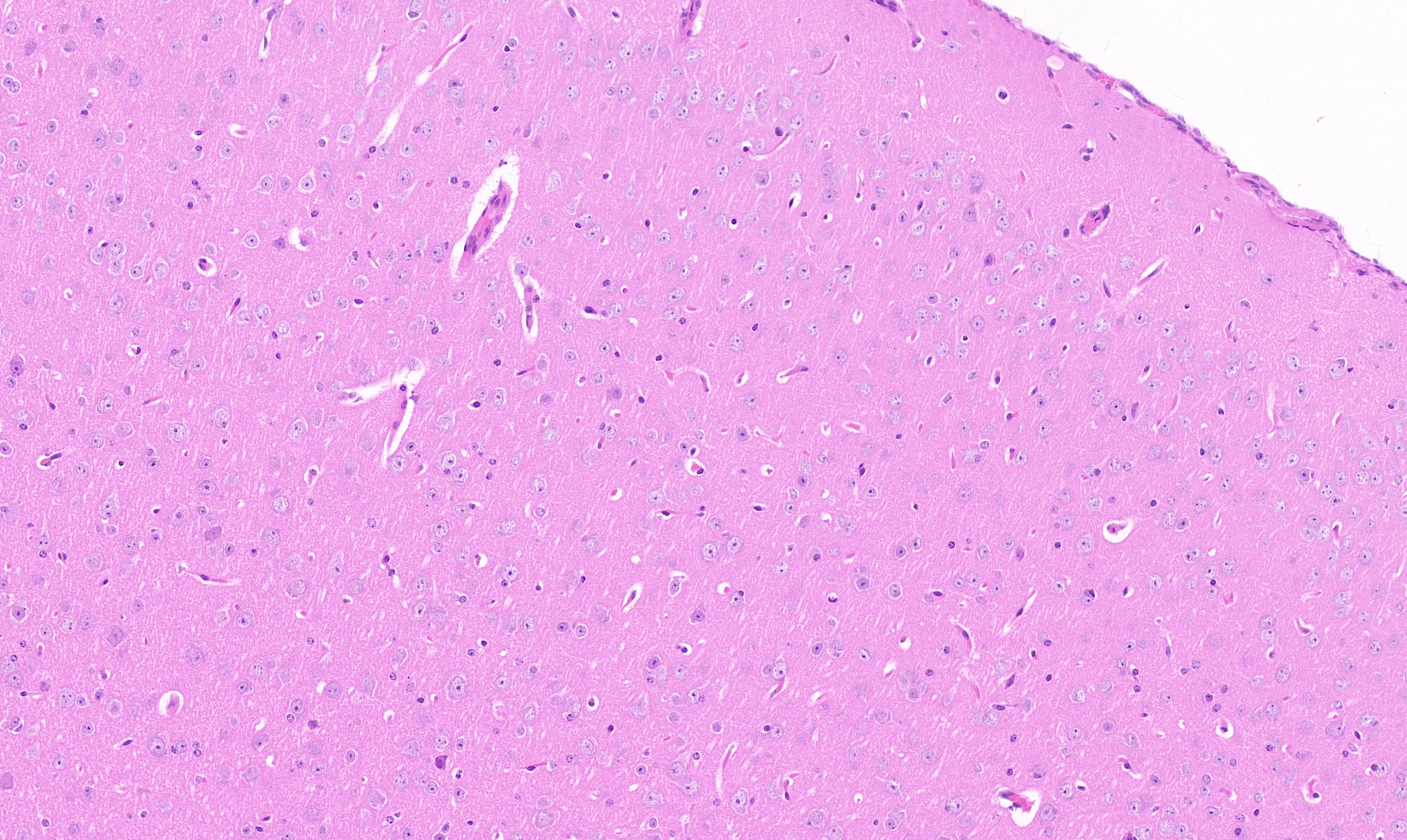

Supplement: Supplementary file 2 [file Presentation_2.ZIP › HE staining/WTE3 ─╘ ╟░╢ε_25.0x.jpg]

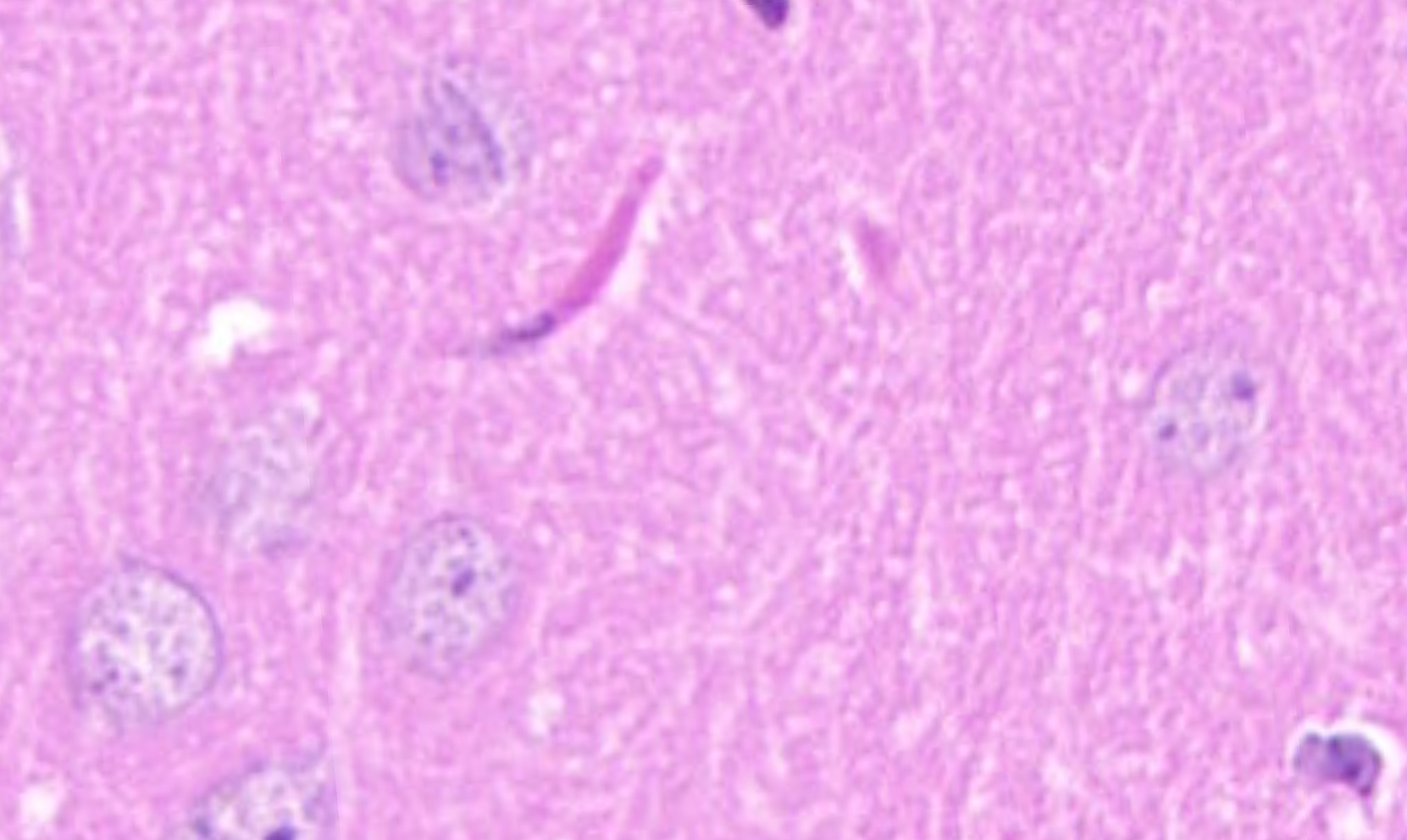

Supplement: Supplementary file 2 [file Presentation_2.ZIP › HE staining/WTE3 ─╘ ╟░╢ε_250.0x.jpg]

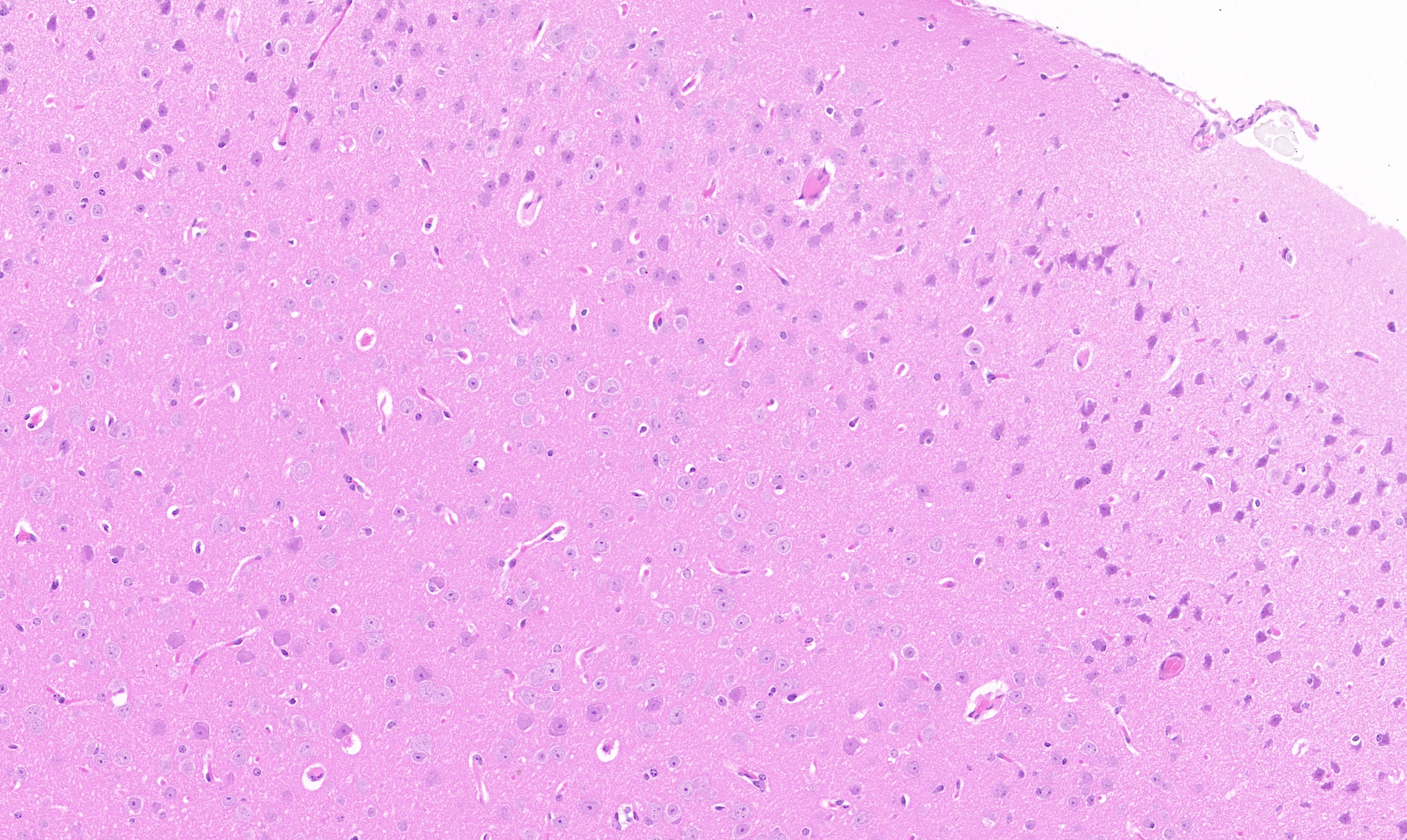

Supplement: Supplementary file 2 [file Presentation_2.ZIP › HE staining/WTS3 ─╘ ╟░╢ε_25.0x.jpg]

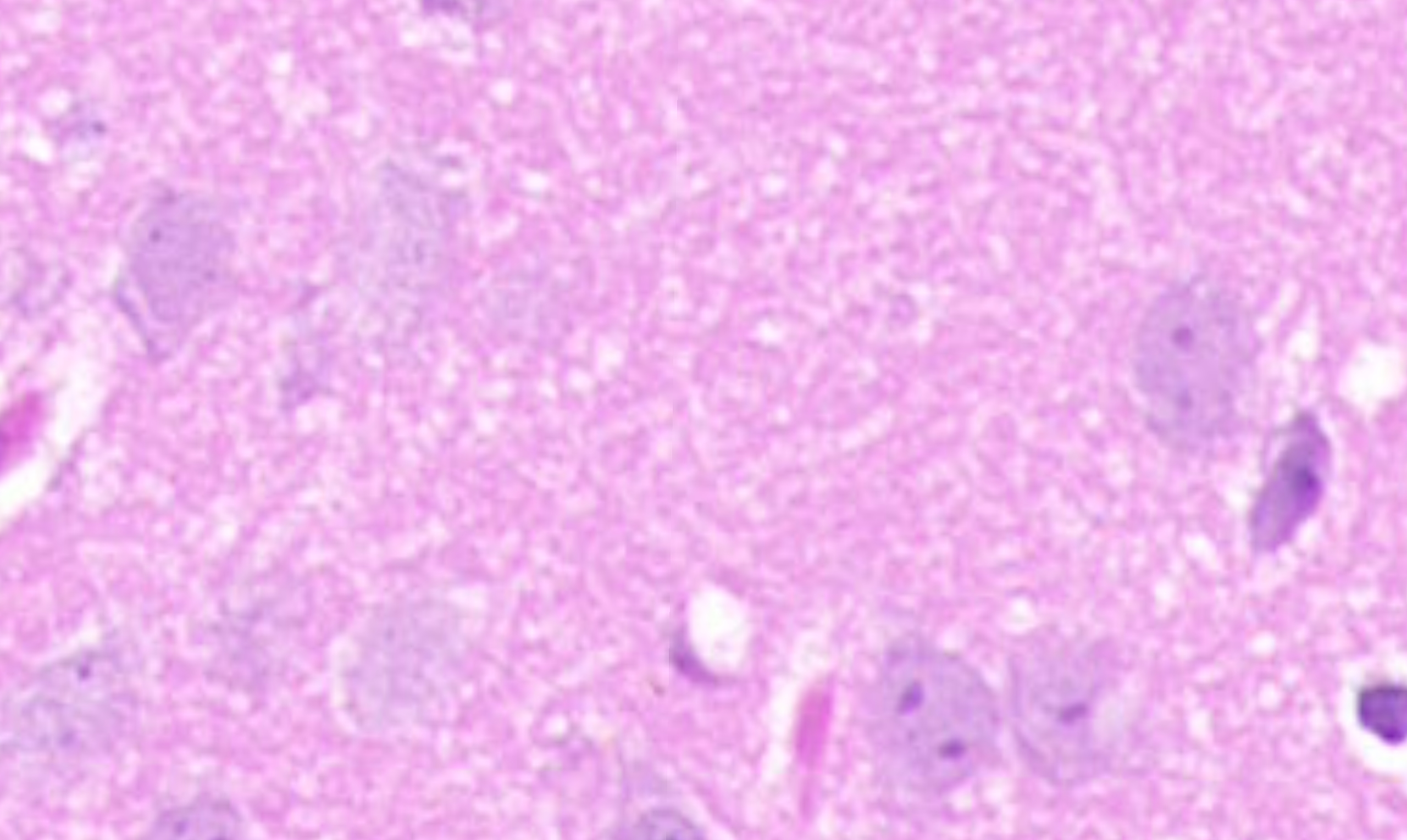

Supplement: Supplementary file 2 [file Presentation_2.ZIP › HE staining/WTS3 ─╘ ╟░╢ε_250.0x.jpg]

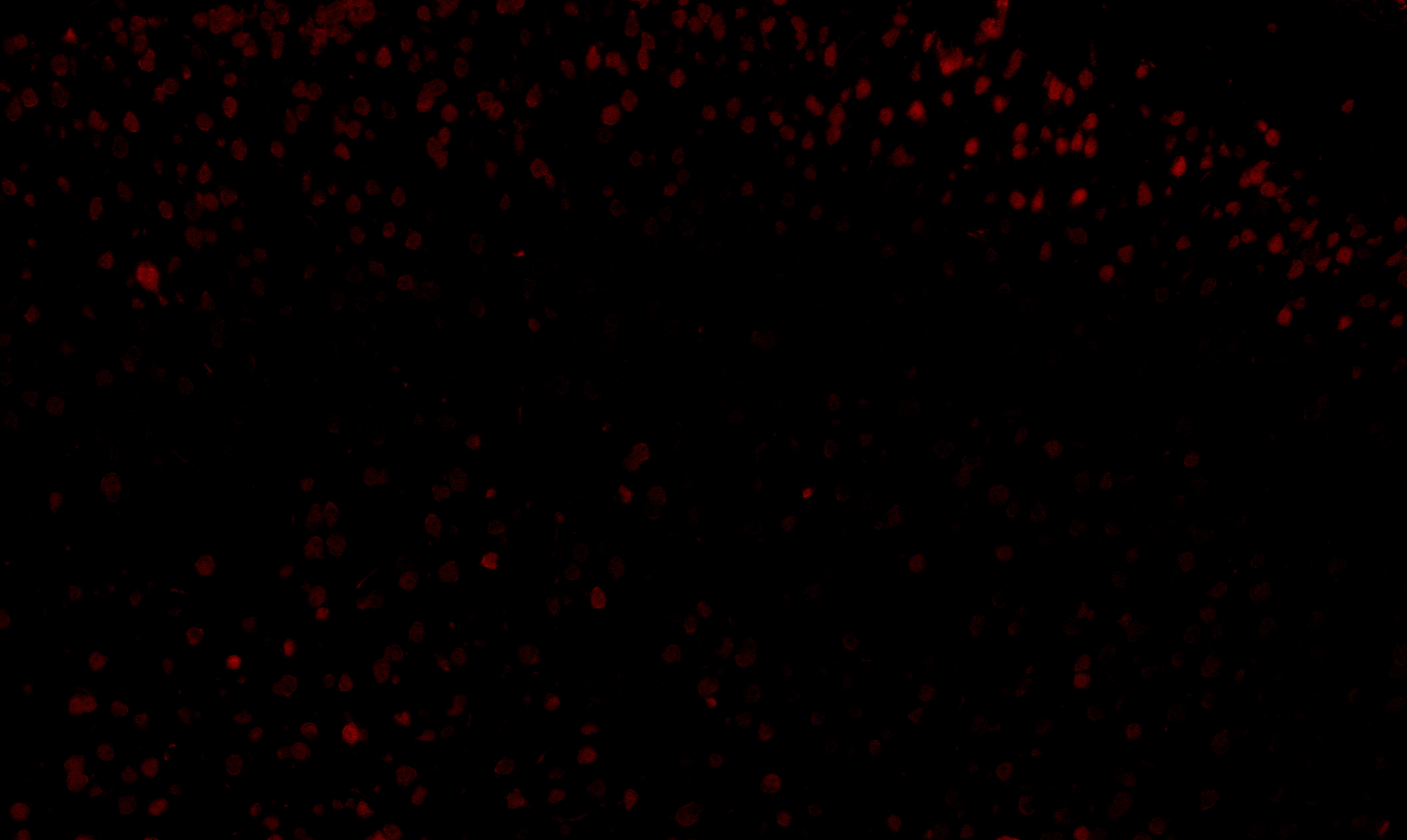

Supplement: Supplementary file 3 [file Presentation_3.ZIP › FJC-NEUN/║∞╔1⁄2ADE3─╘ ╟░╢ε NEUN║∞+FJC┬╠ IF 7_25.0x.jpg]

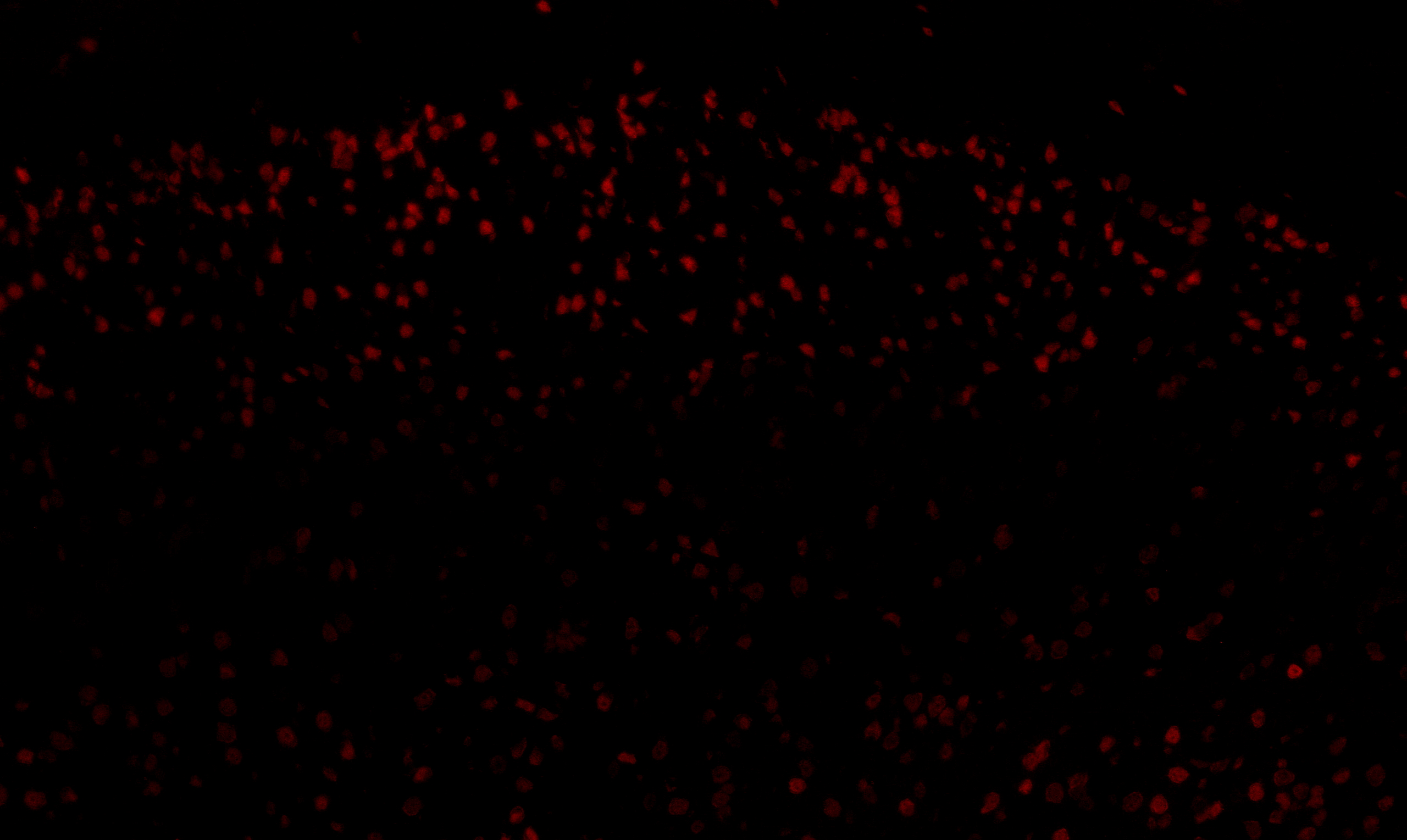

Supplement: Supplementary file 3 [file Presentation_3.ZIP › FJC-NEUN/║∞╔1⁄2ADS1─╘ ╟░╢ε NEUN║∞+FJC┬╠ IF 6_25.0x.jpg]

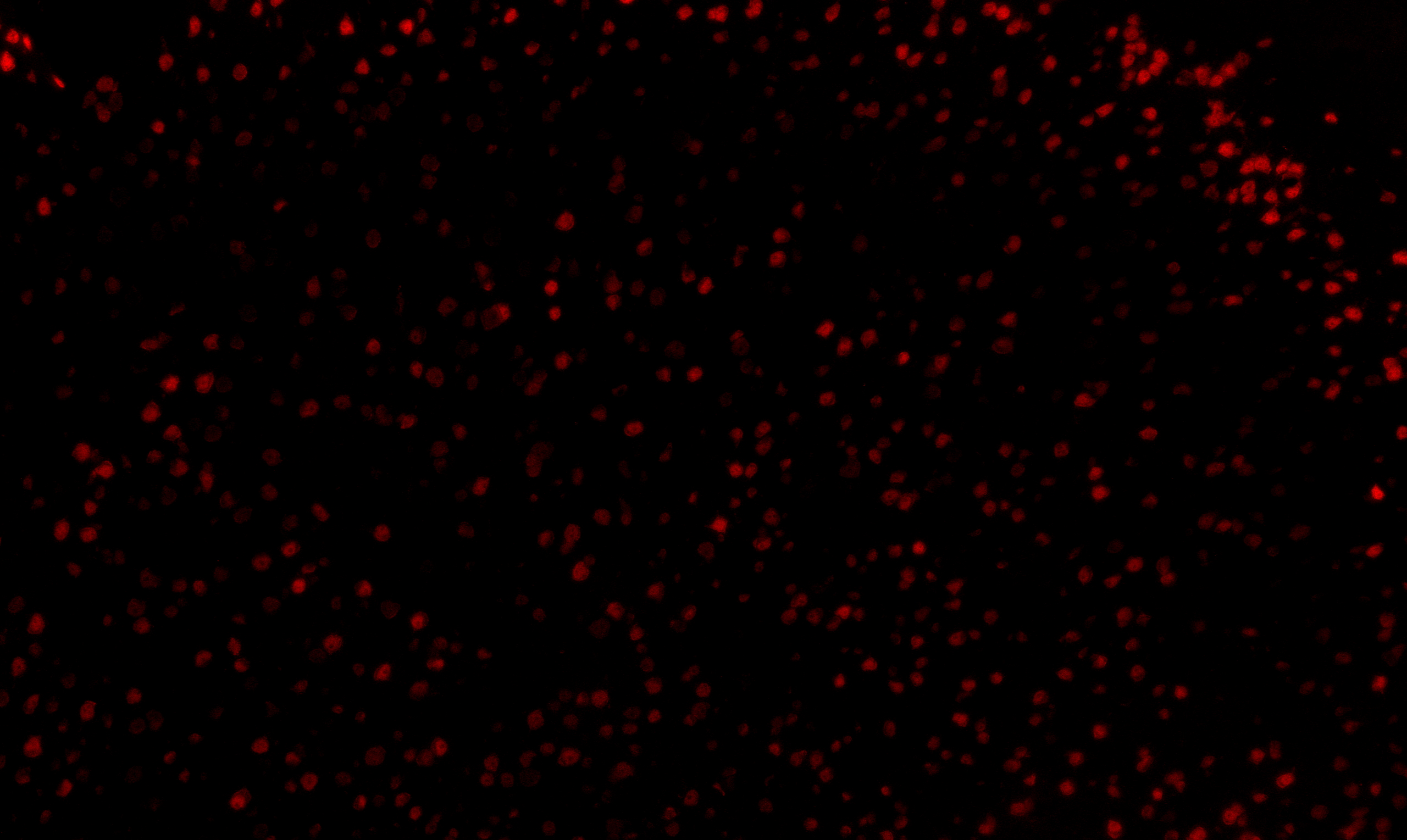

Supplement: Supplementary file 3 [file Presentation_3.ZIP › FJC-NEUN/║∞╔1⁄2WTE1─╘ ╟░╢ε NEUN║∞+FJC┬╠ IF 7_25.0x.jpg]

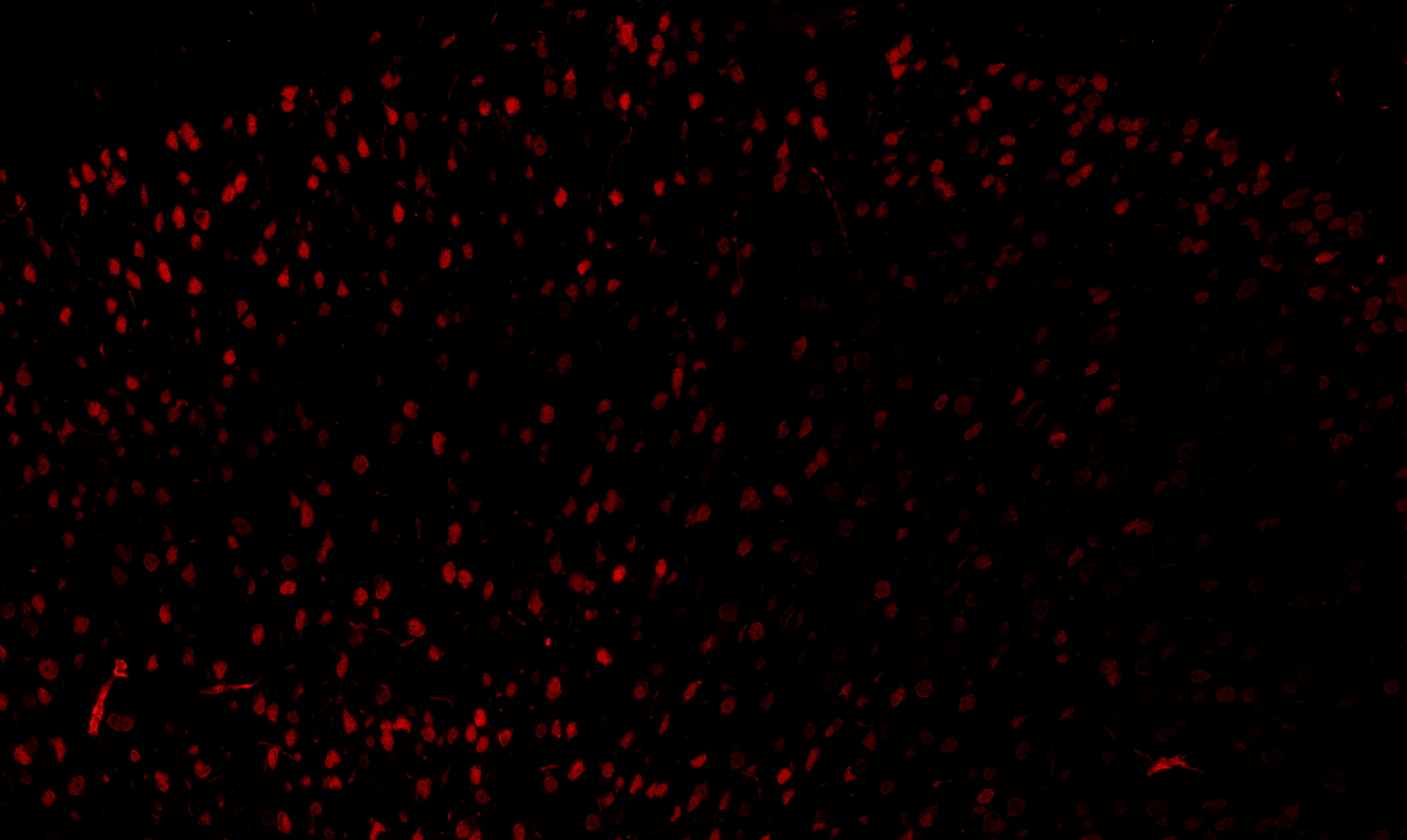

Supplement: Supplementary file 3 [file Presentation_3.ZIP › FJC-NEUN/║∞╔1⁄2WTS-3─╘ ╟░╢ε └Φ│1⁄4╤≤IF-NEUNú¿Rú⌐+FJCú¿Gú⌐-6_25.0x.jpg]

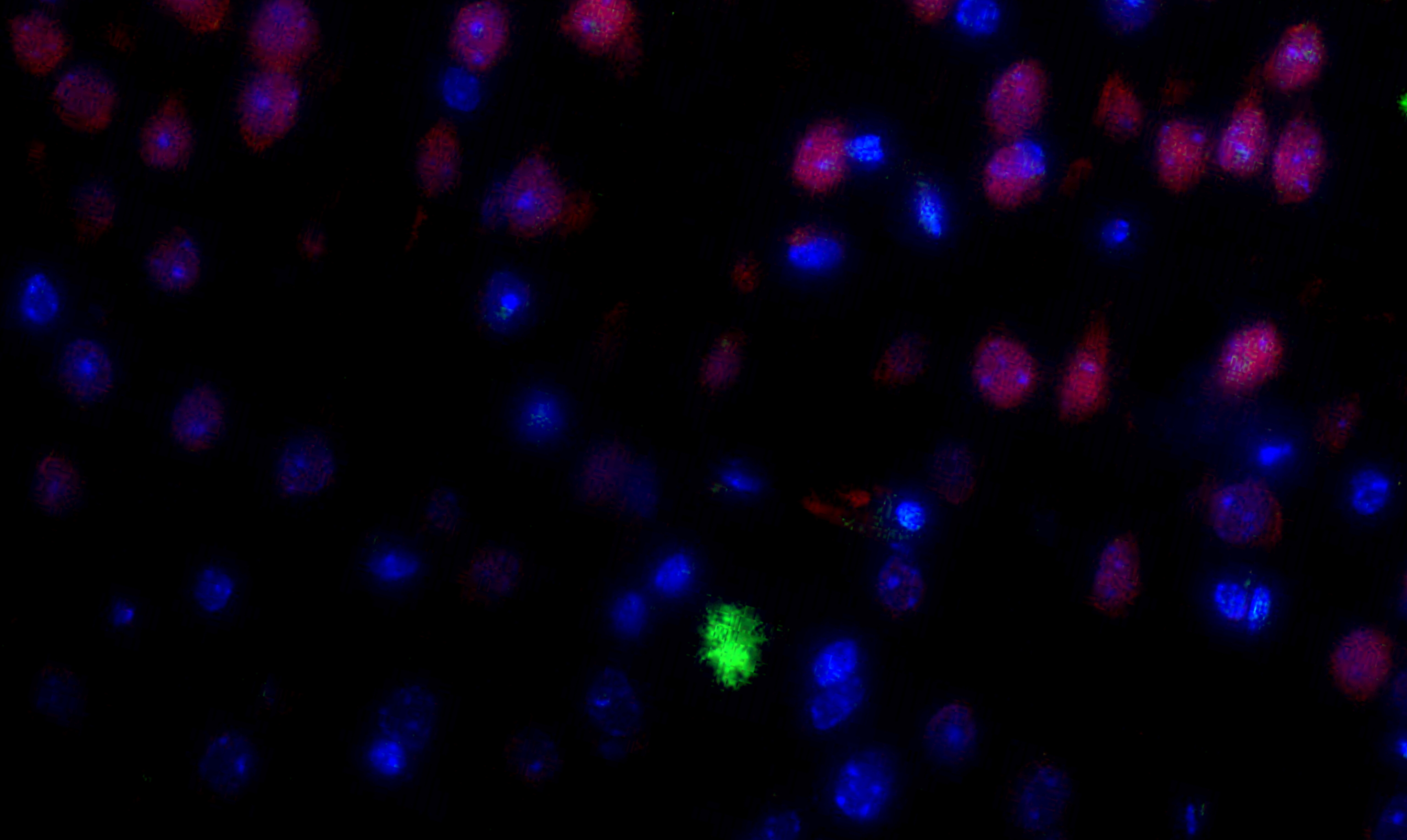

Supplement: Supplementary file 3 [file Presentation_3.ZIP › FJC-NEUN/╗∞║╧ADE3─╘ ╟░╢ε NEUN║∞+FJC┬╠ IF 7_100.0x.jpg]

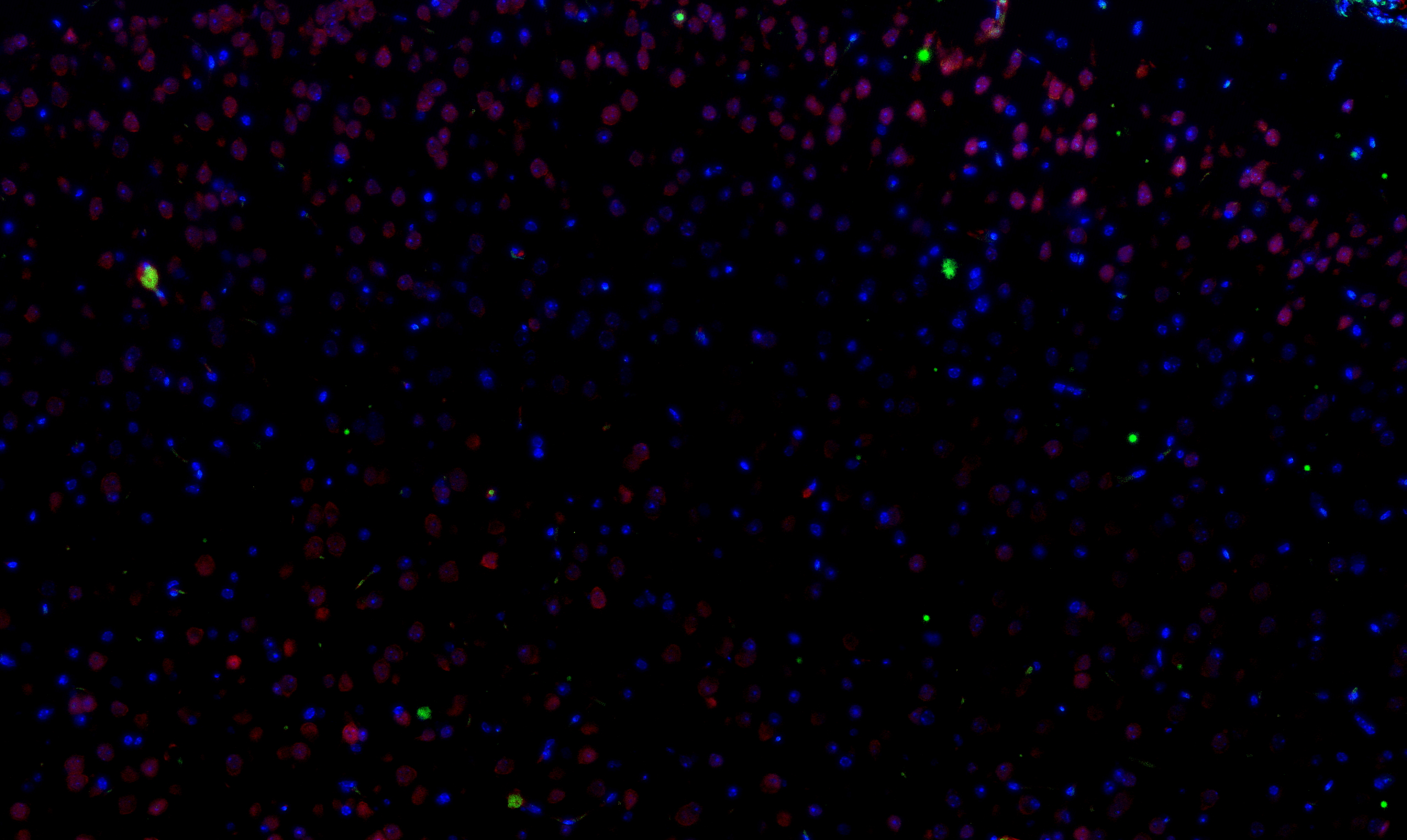

Supplement: Supplementary file 3 [file Presentation_3.ZIP › FJC-NEUN/╗∞║╧ADE3─╘ ╟░╢ε NEUN║∞+FJC┬╠ IF 7_25.0x.jpg]

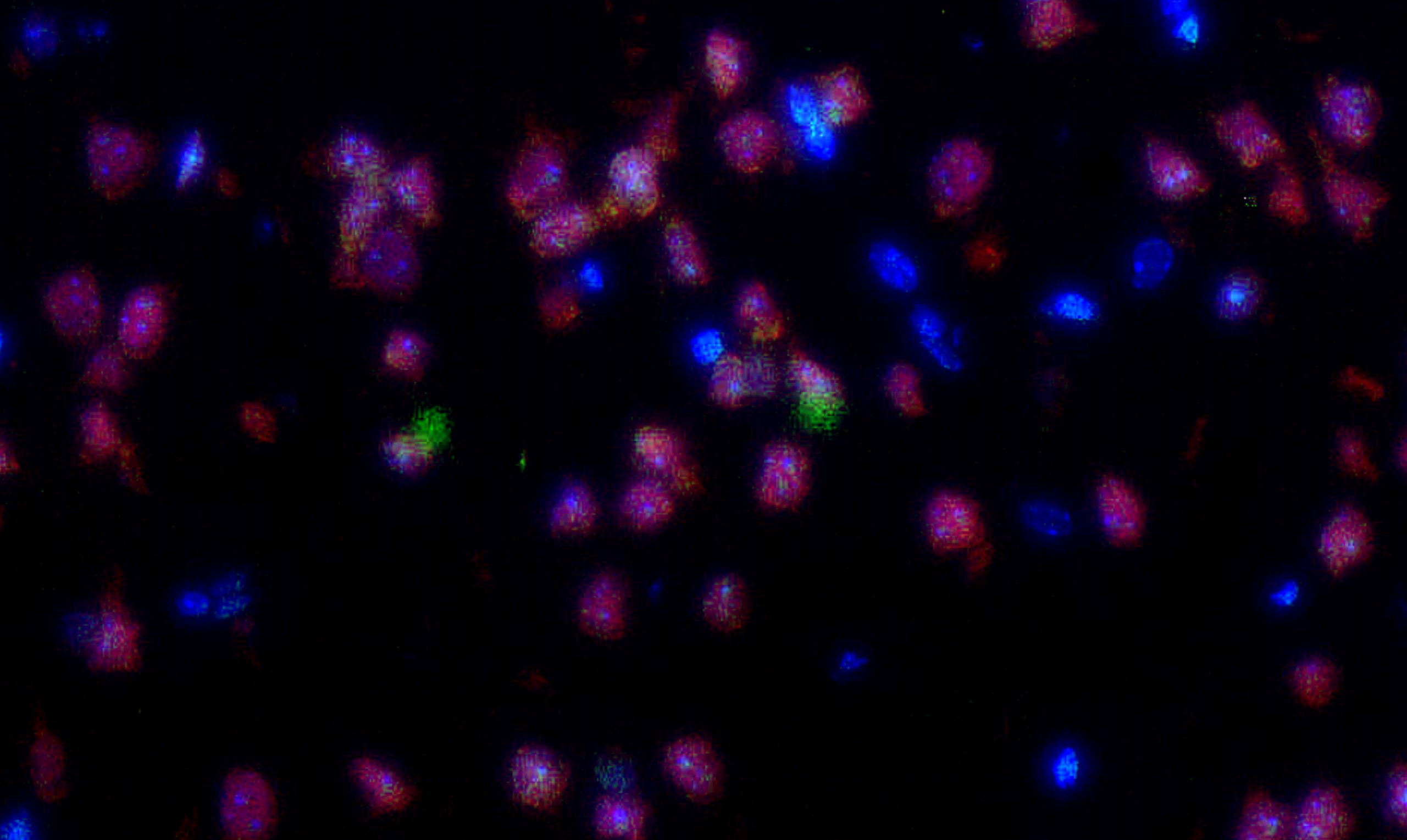

Supplement: Supplementary file 3 [file Presentation_3.ZIP › FJC-NEUN/╗∞║╧ADS1─╘ ╟░╢ε NEUN║∞+FJC┬╠ IF 6_100.0x.jpg]

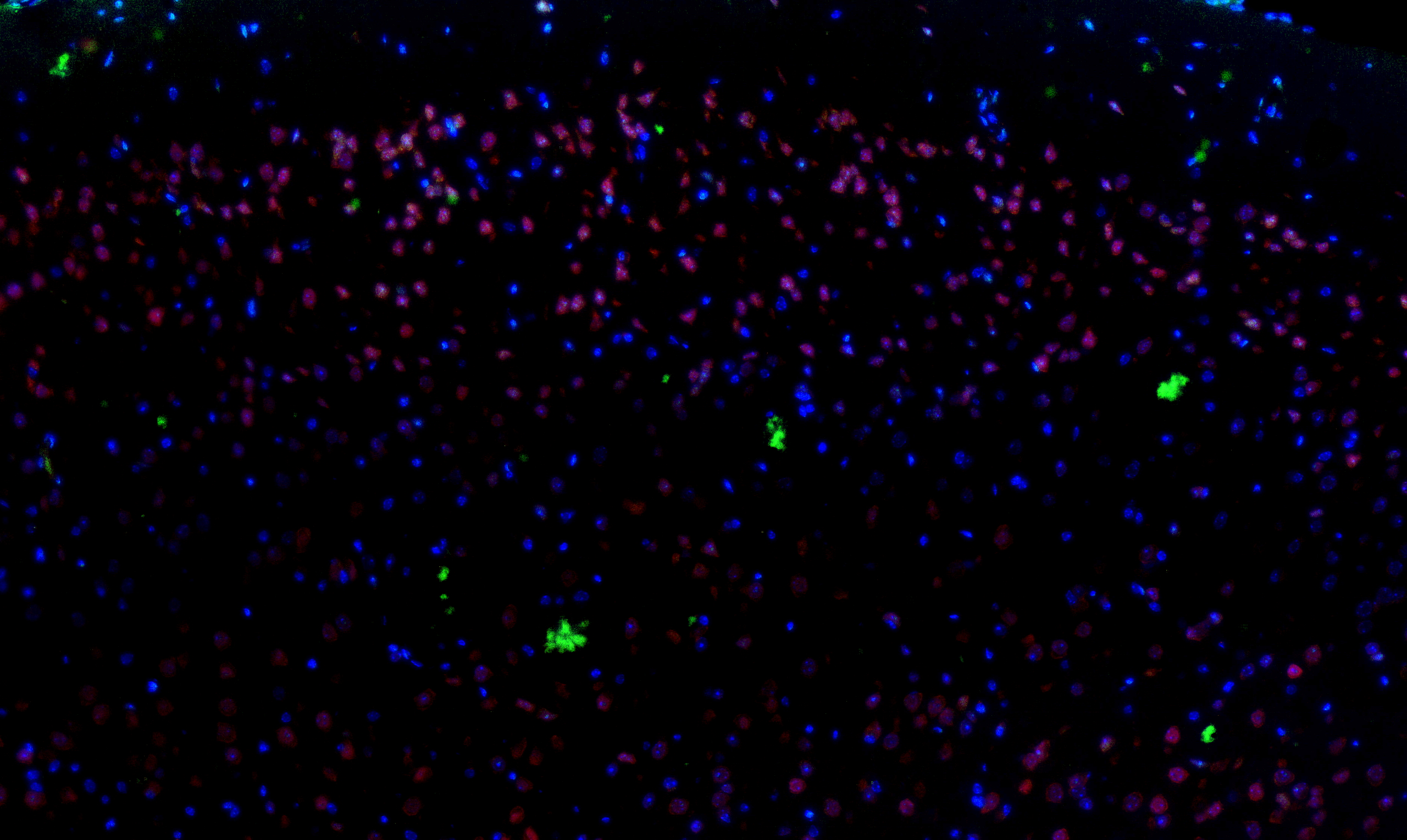

Supplement: Supplementary file 3 [file Presentation_3.ZIP › FJC-NEUN/╗∞║╧ADS1─╘ ╟░╢ε NEUN║∞+FJC┬╠ IF 6_25.0x.jpg]

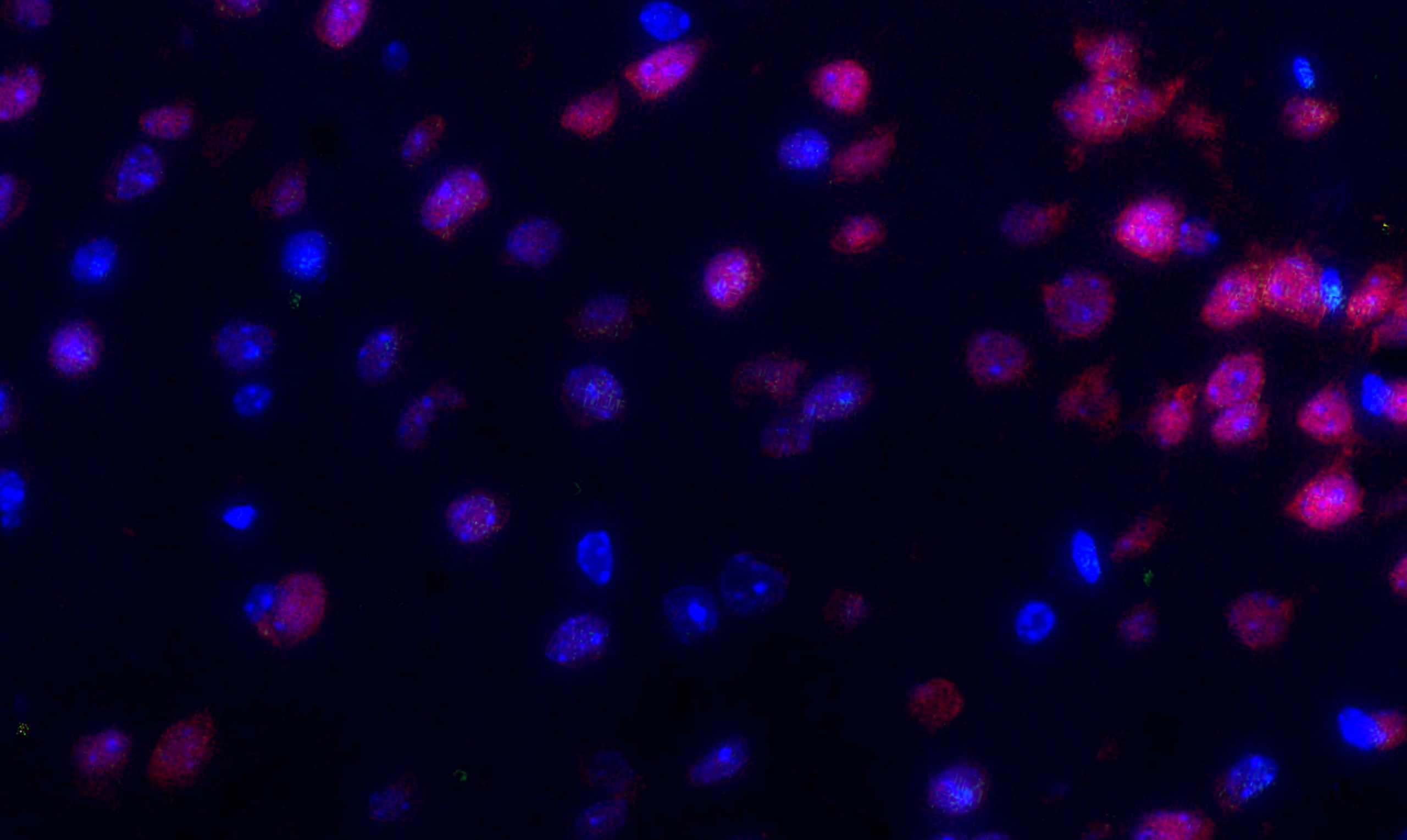

Supplement: Supplementary file 3 [file Presentation_3.ZIP › FJC-NEUN/╗∞║╧WTE1─╘ ╟░╢ε NEUN║∞+FJC┬╠ IF 7_100.0x.jpg]

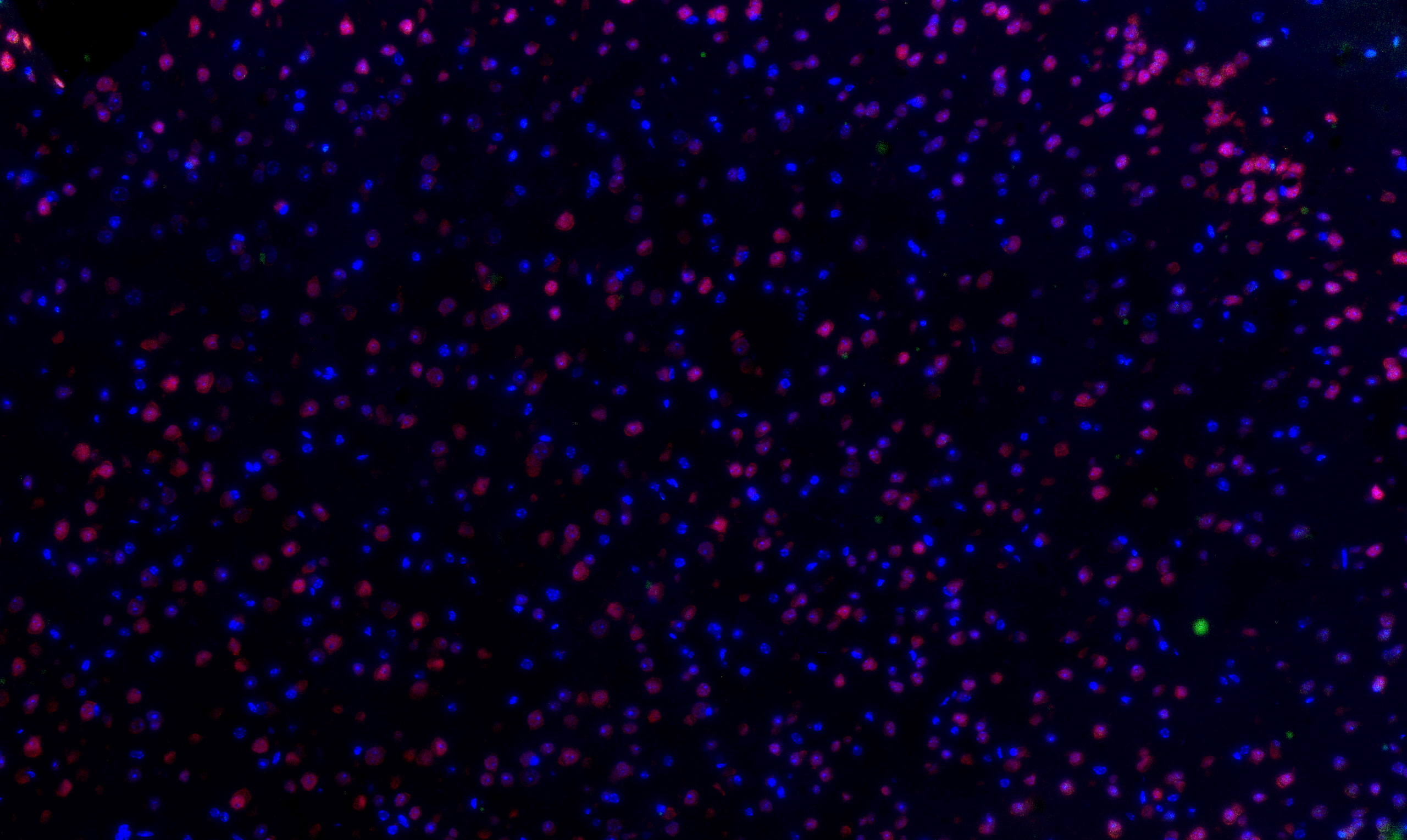

Supplement: Supplementary file 3 [file Presentation_3.ZIP › FJC-NEUN/╗∞║╧WTE1─╘ ╟░╢ε NEUN║∞+FJC┬╠ IF 7_25.0x.jpg]

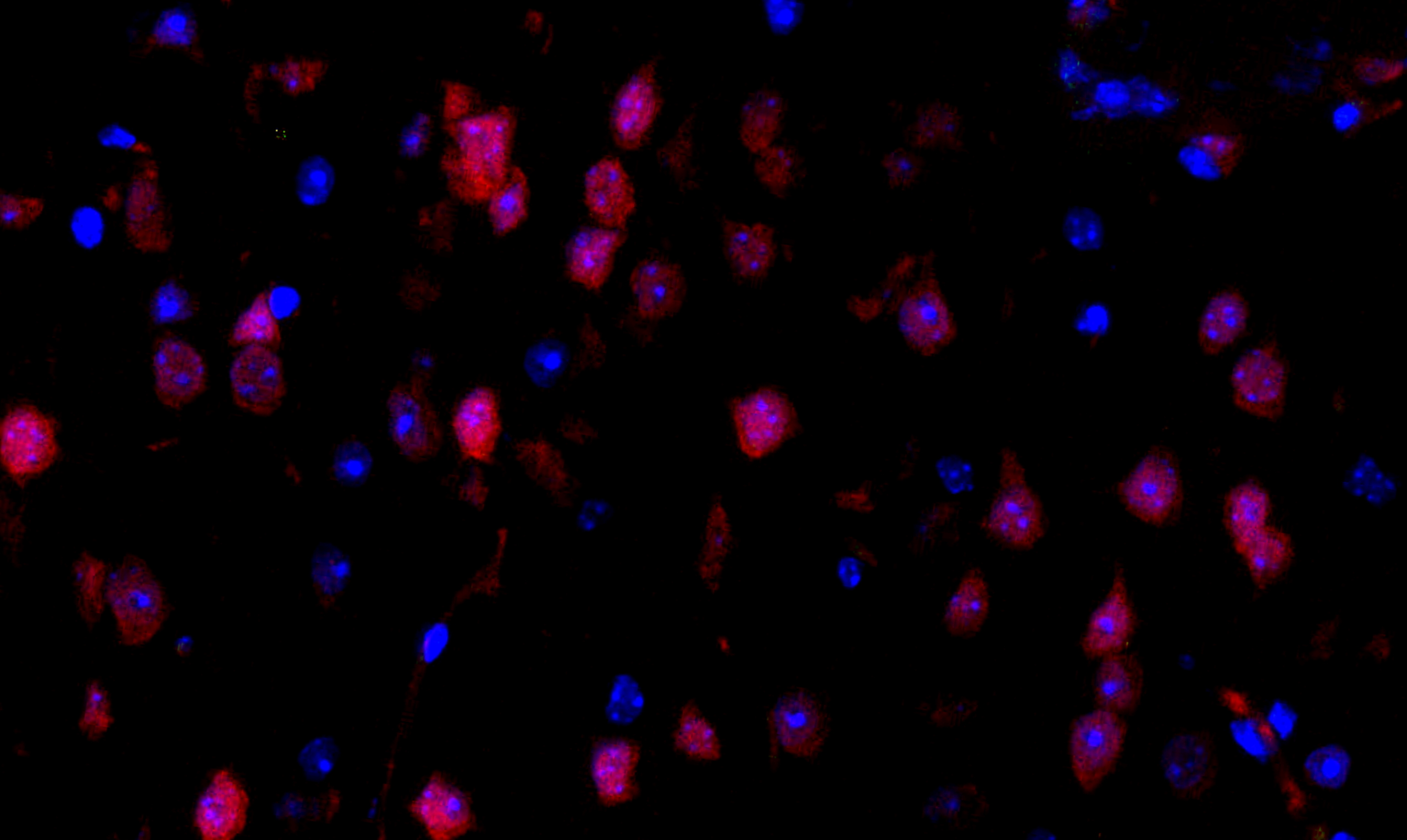

Supplement: Supplementary file 3 [file Presentation_3.ZIP › FJC-NEUN/╗∞║╧WTS-3─╘ ╟░╢ε └Φ│1⁄4╤≤IF-NEUNú¿Rú⌐+FJCú¿Gú⌐-6_100.0x.jpg]

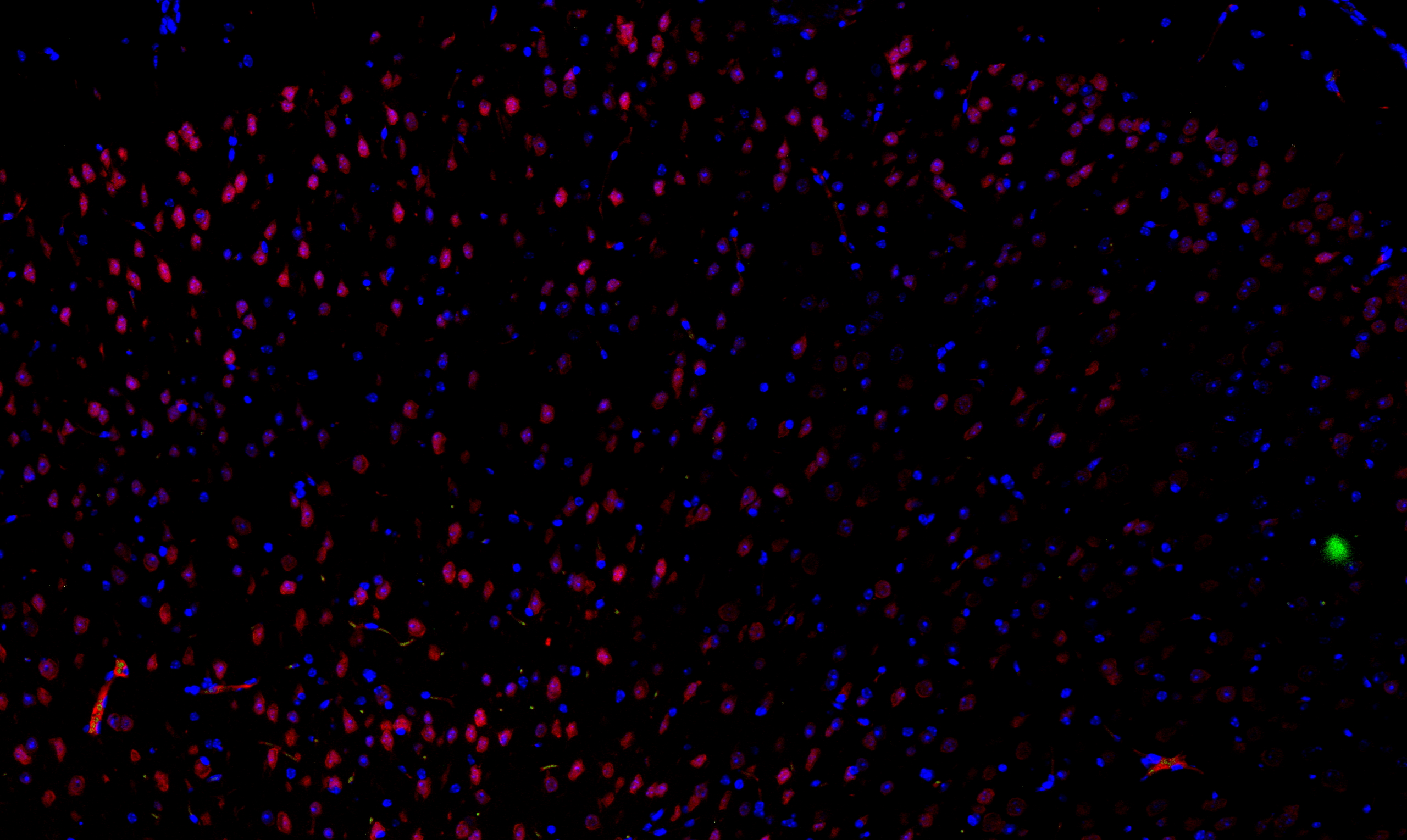

Supplement: Supplementary file 3 [file Presentation_3.ZIP › FJC-NEUN/╗∞║╧WTS-3─╘ ╟░╢ε └Φ│1⁄4╤≤IF-NEUNú¿Rú⌐+FJCú¿Gú⌐-6_25.0x.jpg]

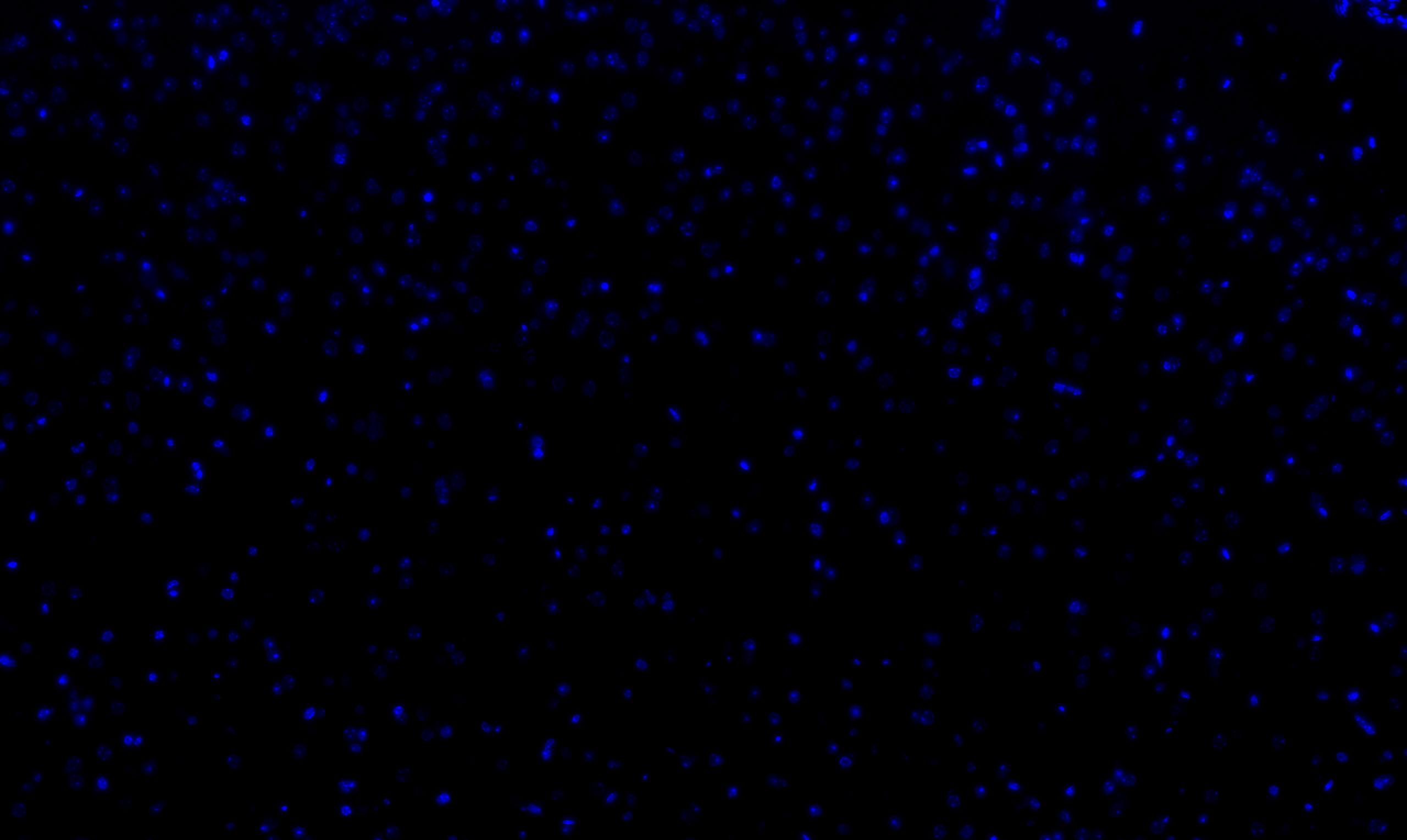

Supplement: Supplementary file 3 [file Presentation_3.ZIP › FJC-NEUN/└╢╔1⁄2ADE3─╘ ╟░╢ε NEUN║∞+FJC┬╠ IF 7_25.0x.jpg]

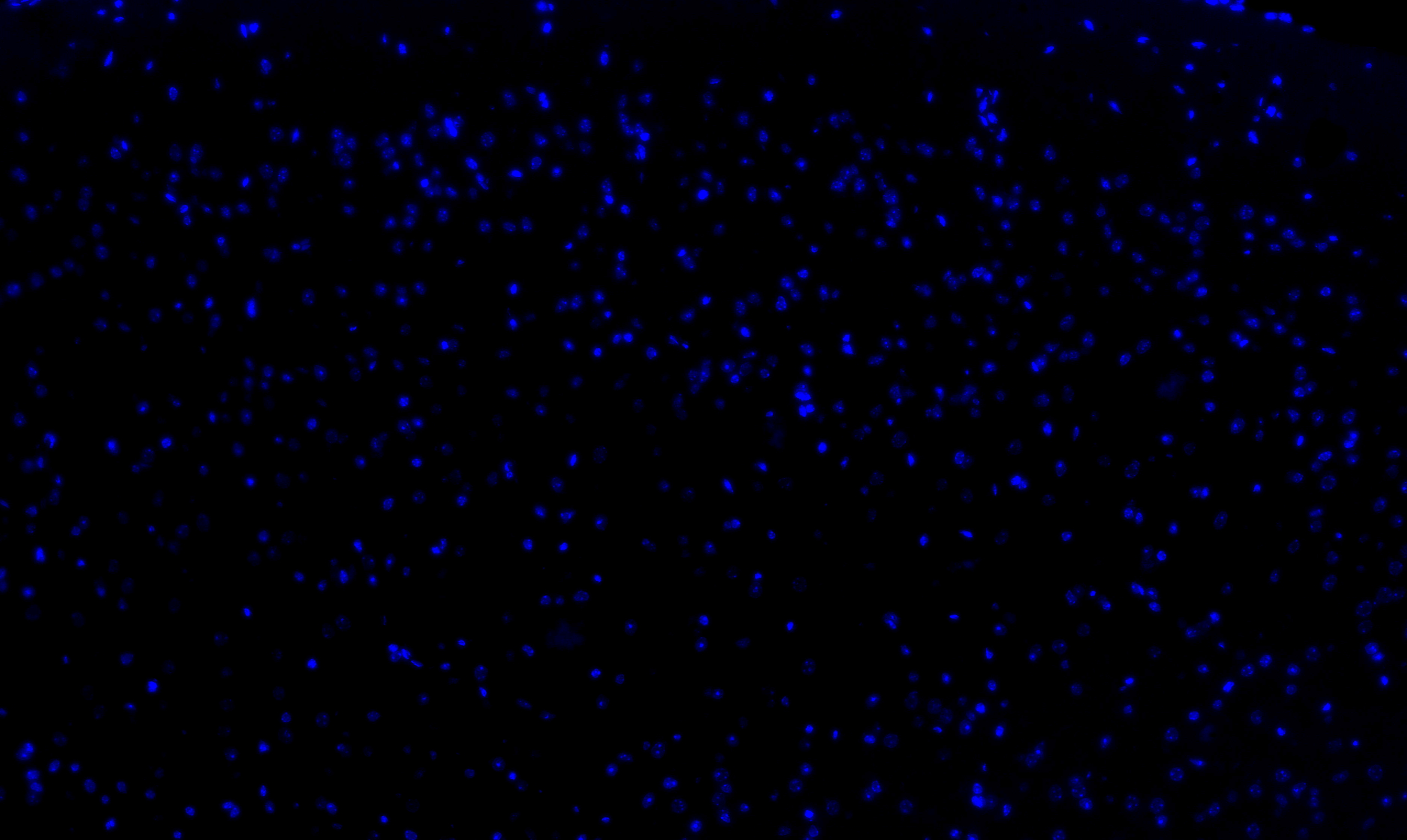

Supplement: Supplementary file 3 [file Presentation_3.ZIP › FJC-NEUN/└╢╔1⁄2ADS1─╘ ╟░╢ε NEUN║∞+FJC┬╠ IF 6_25.0x.jpg]

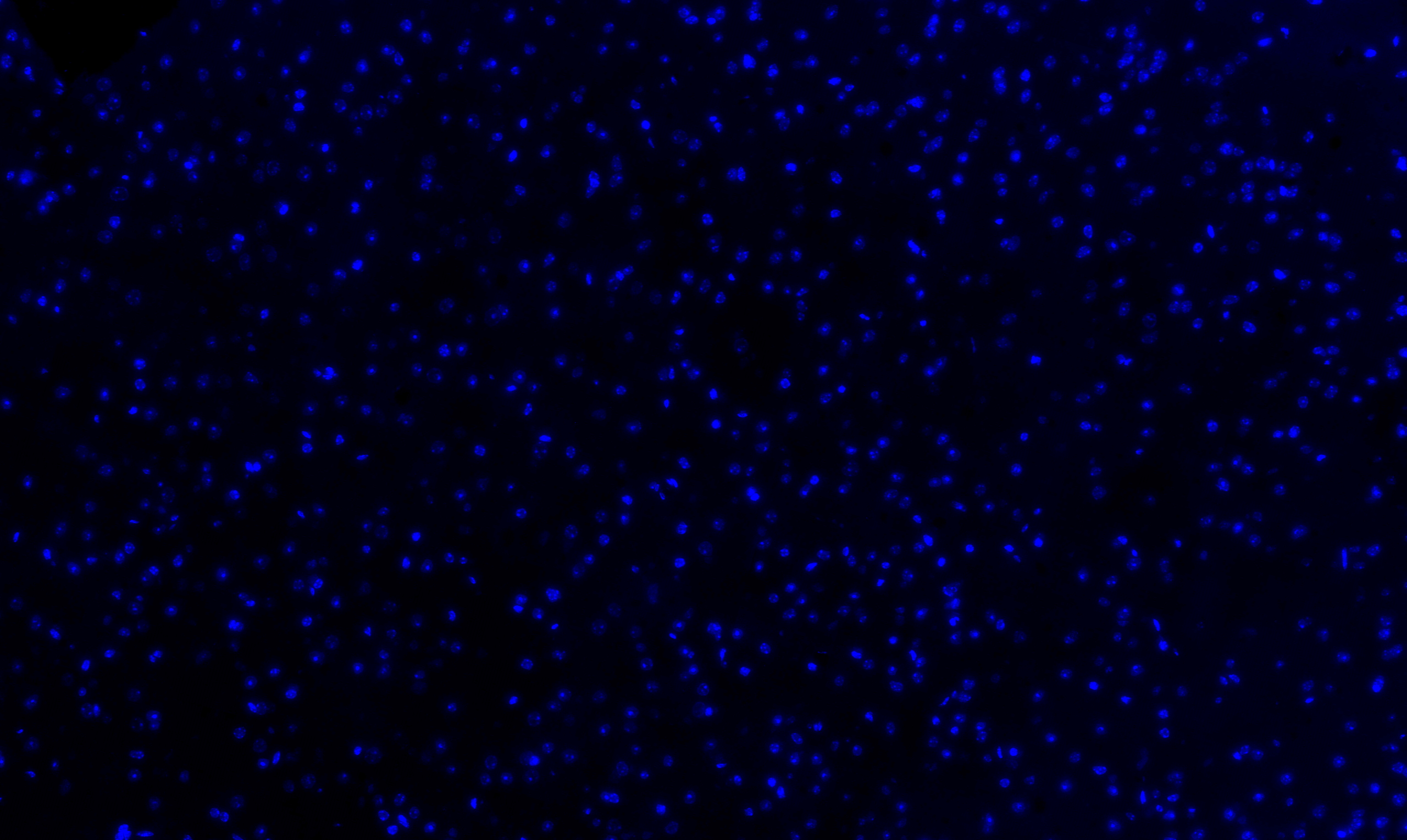

Supplement: Supplementary file 3 [file Presentation_3.ZIP › FJC-NEUN/└╢╔1⁄2WTE1─╘ ╟░╢ε NEUN║∞+FJC┬╠ IF 7_25.0x.jpg]

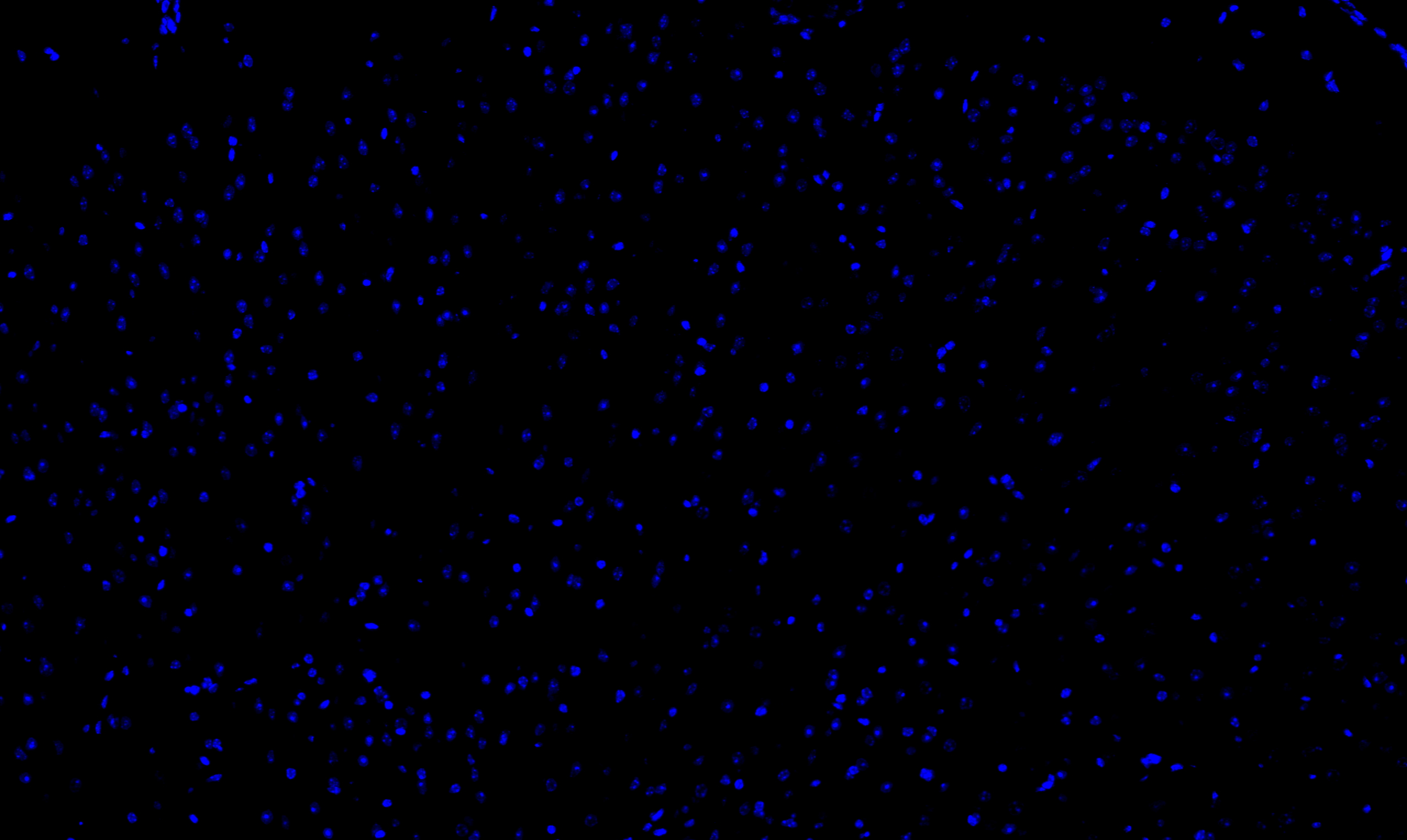

Supplement: Supplementary file 3 [file Presentation_3.ZIP › FJC-NEUN/└╢╔1⁄2WTS-3─╘ ╟░╢ε └Φ│1⁄4╤≤IF-NEUNú¿Rú⌐+FJCú¿Gú⌐-6_25.0x.jpg]

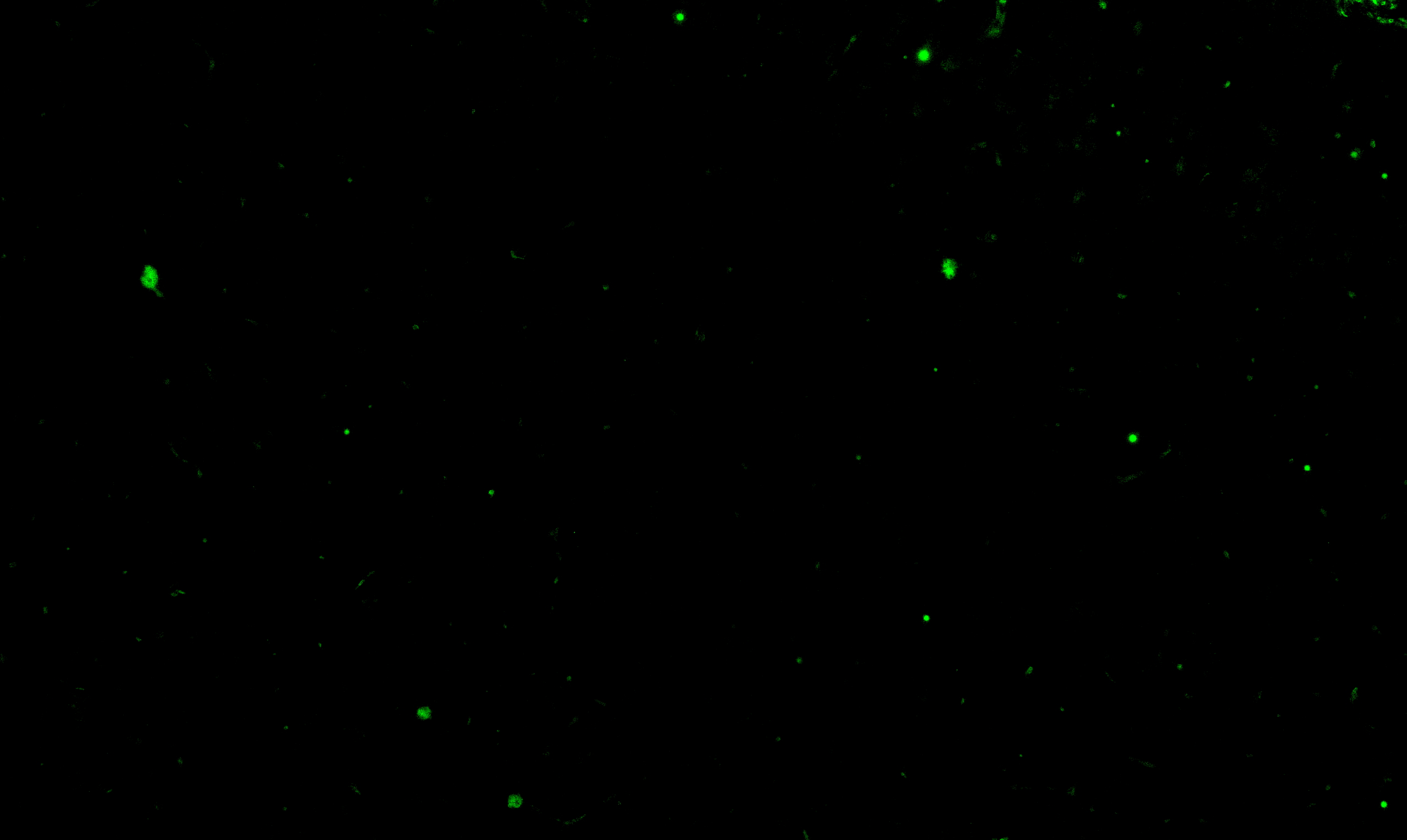

Supplement: Supplementary file 3 [file Presentation_3.ZIP › FJC-NEUN/┬╠╔1⁄2ADE3─╘ ╟░╢ε NEUN║∞+FJC┬╠ IF 7_25.0x.jpg]

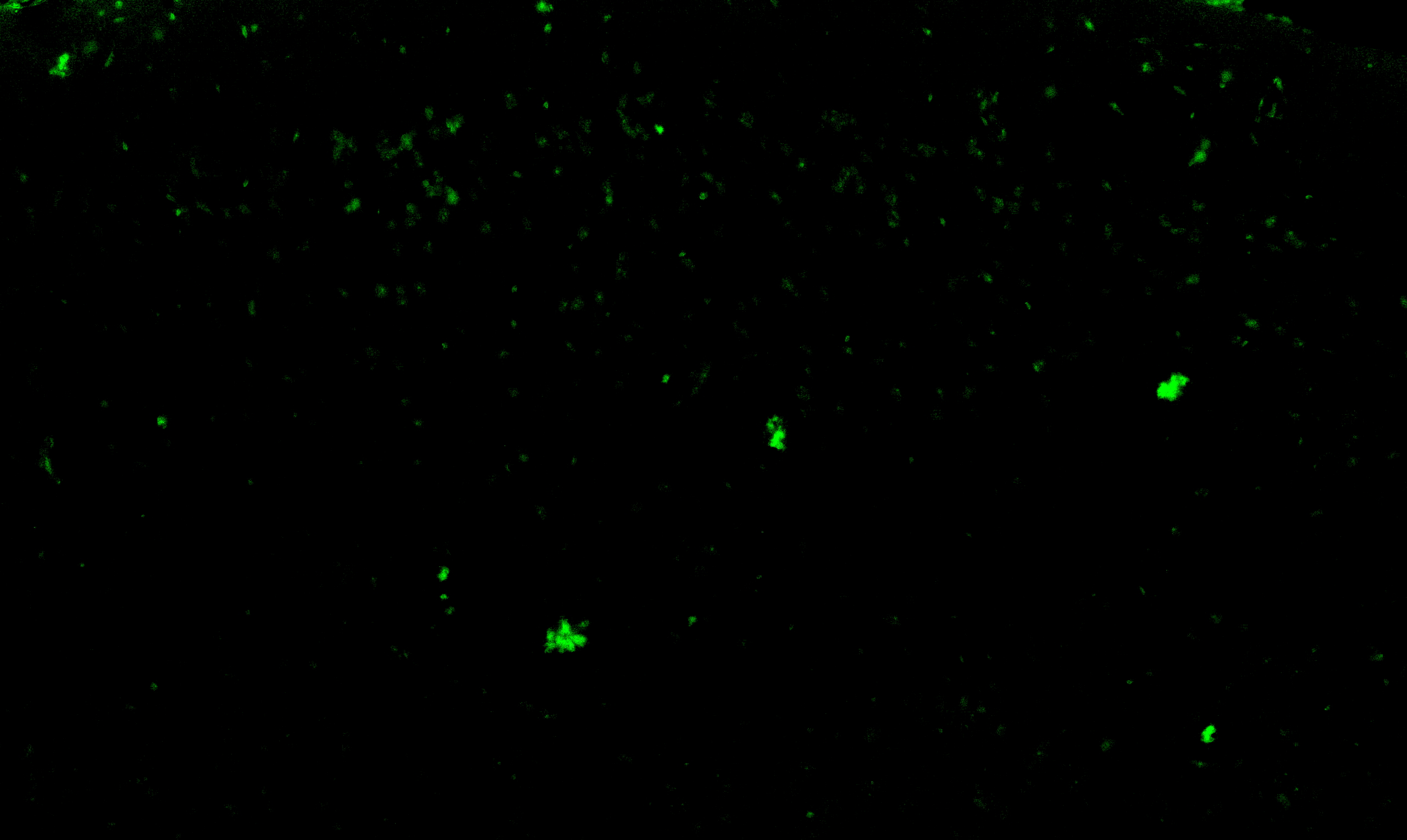

Supplement: Supplementary file 3 [file Presentation_3.ZIP › FJC-NEUN/┬╠╔1⁄2ADS1─╘ ╟░╢ε NEUN║∞+FJC┬╠ IF 6_25.0x.jpg]

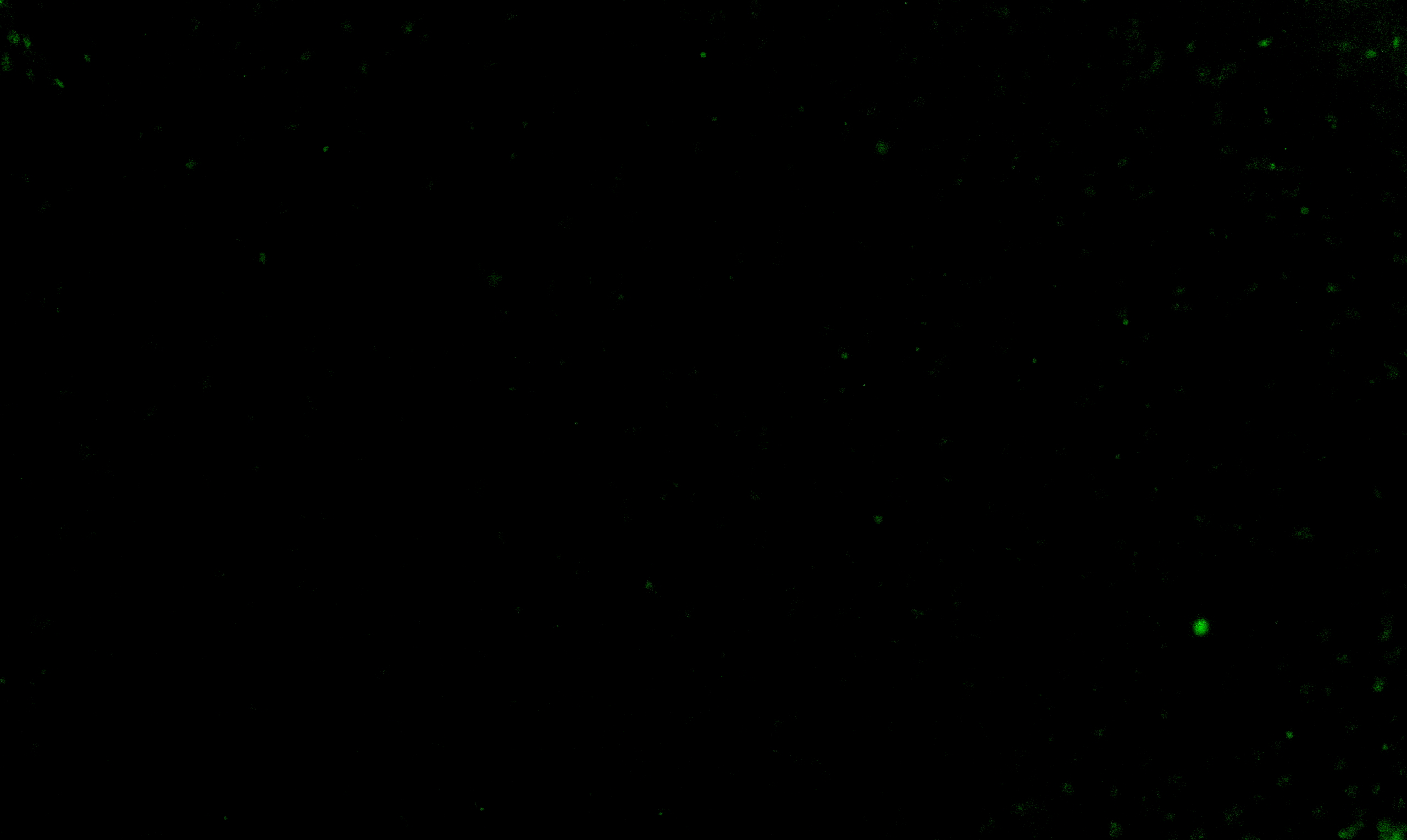

Supplement: Supplementary file 3 [file Presentation_3.ZIP › FJC-NEUN/┬╠╔1⁄2WTE1─╘ ╟░╢ε NEUN║∞+FJC┬╠ IF 7_25.0x.jpg]

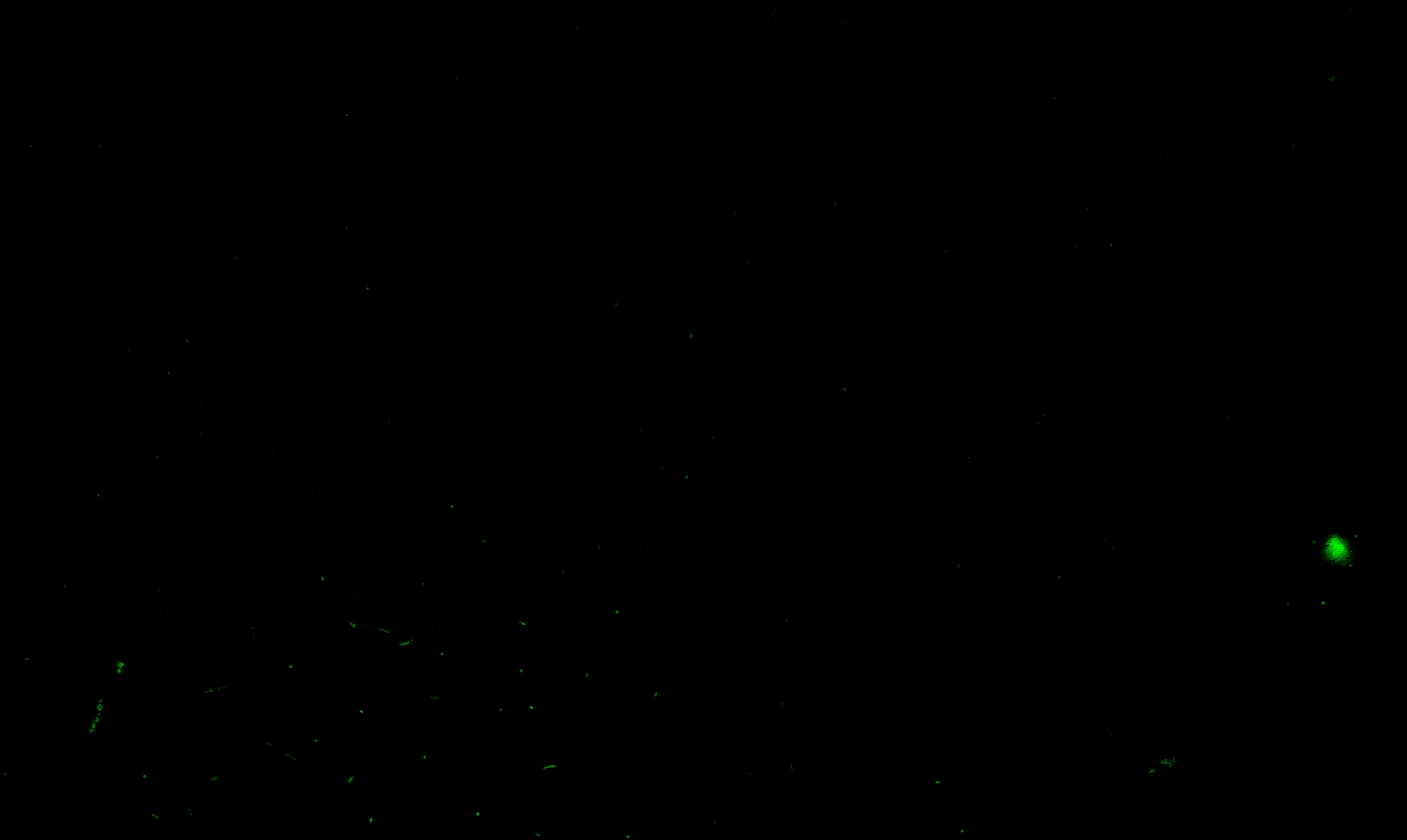

Supplement: Supplementary file 3 [file Presentation_3.ZIP › FJC-NEUN/┬╠╔1⁄2WTS-3─╘ ╟░╢ε └Φ│1⁄4╤≤IF-NEUNú¿Rú⌐+FJCú¿Gú⌐-6_25.0x.jpg]

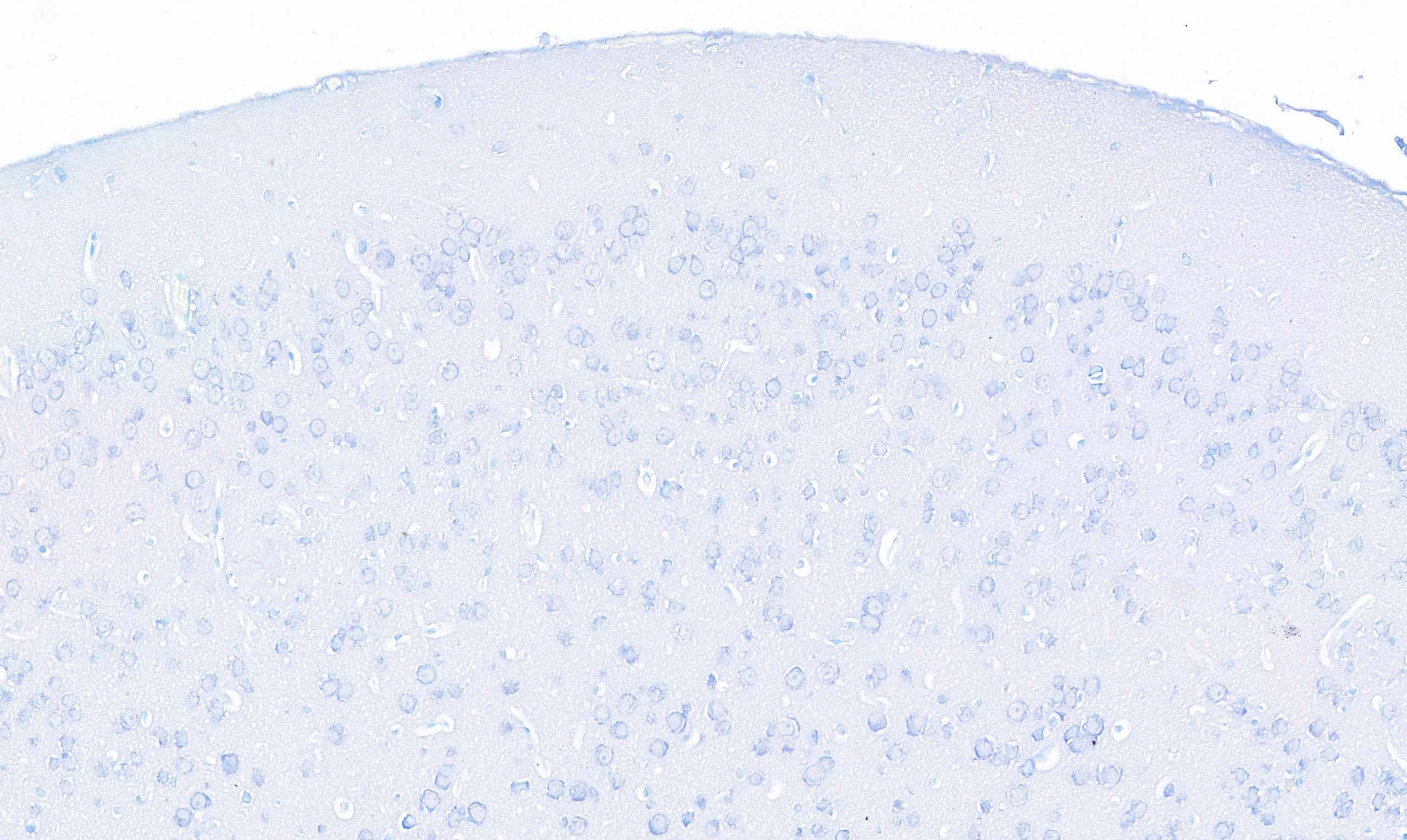

Supplement: Supplementary file 4 [file Presentation_4.ZIP › Nissle staining/ADE3 ─╘ ╟░╢ε ─ß╩╧_25.0x.jpg]

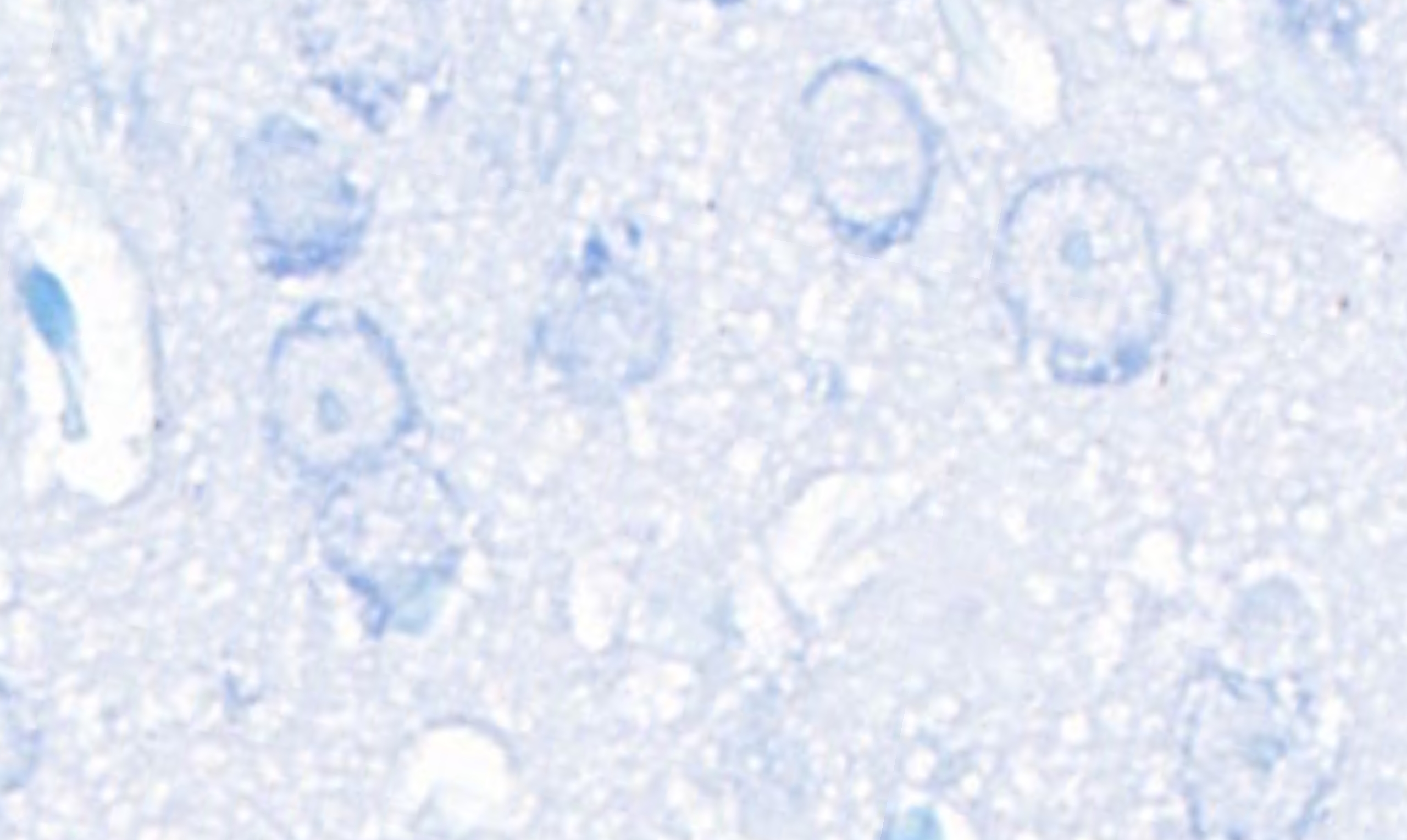

Supplement: Supplementary file 4 [file Presentation_4.ZIP › Nissle staining/ADE3 ─╘ ╟░╢ε ─ß╩╧_250.0x.jpg]

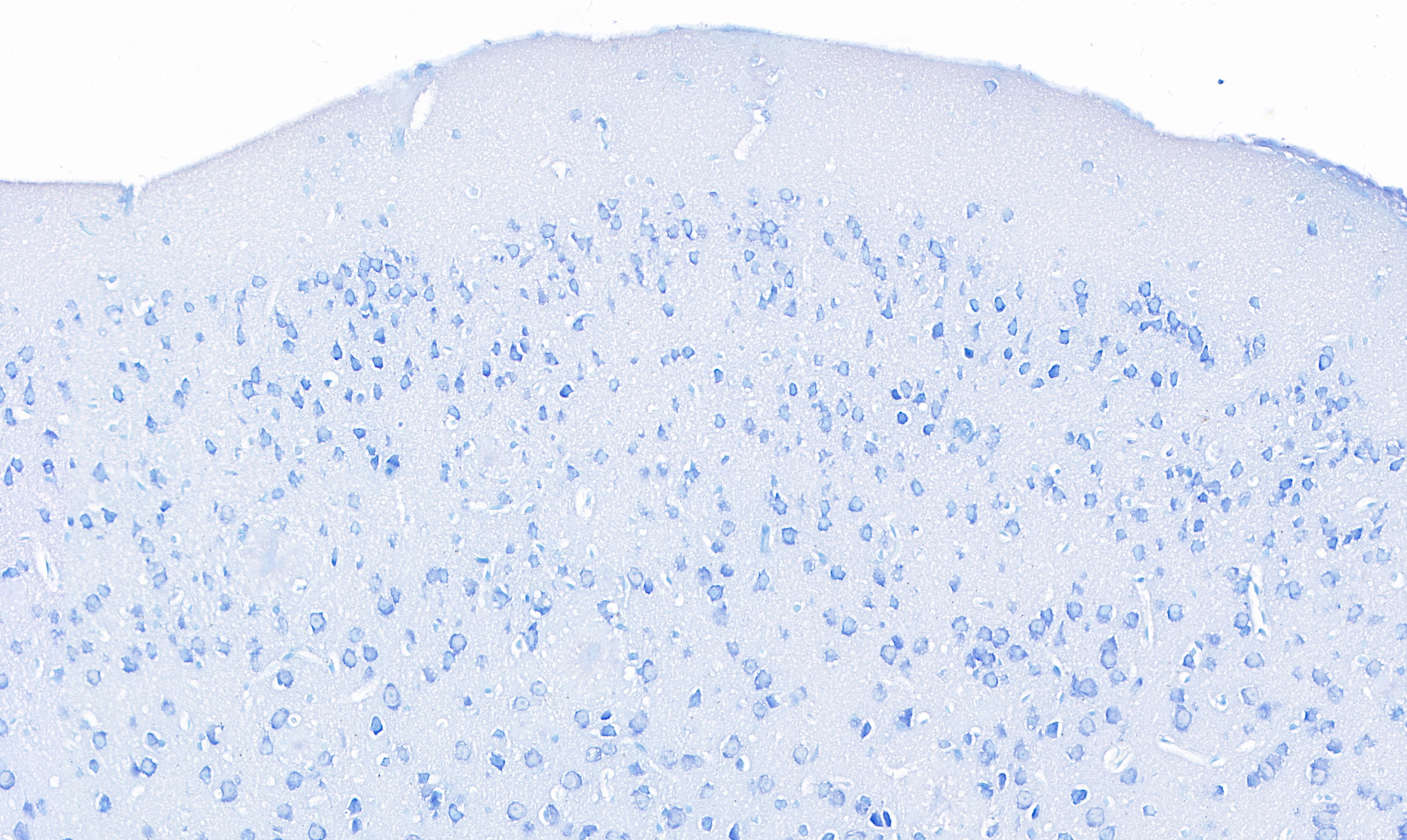

Supplement: Supplementary file 4 [file Presentation_4.ZIP › Nissle staining/ADS3 ─╘ ╟░╢ε ─ß╩╧_25.0x.jpg]

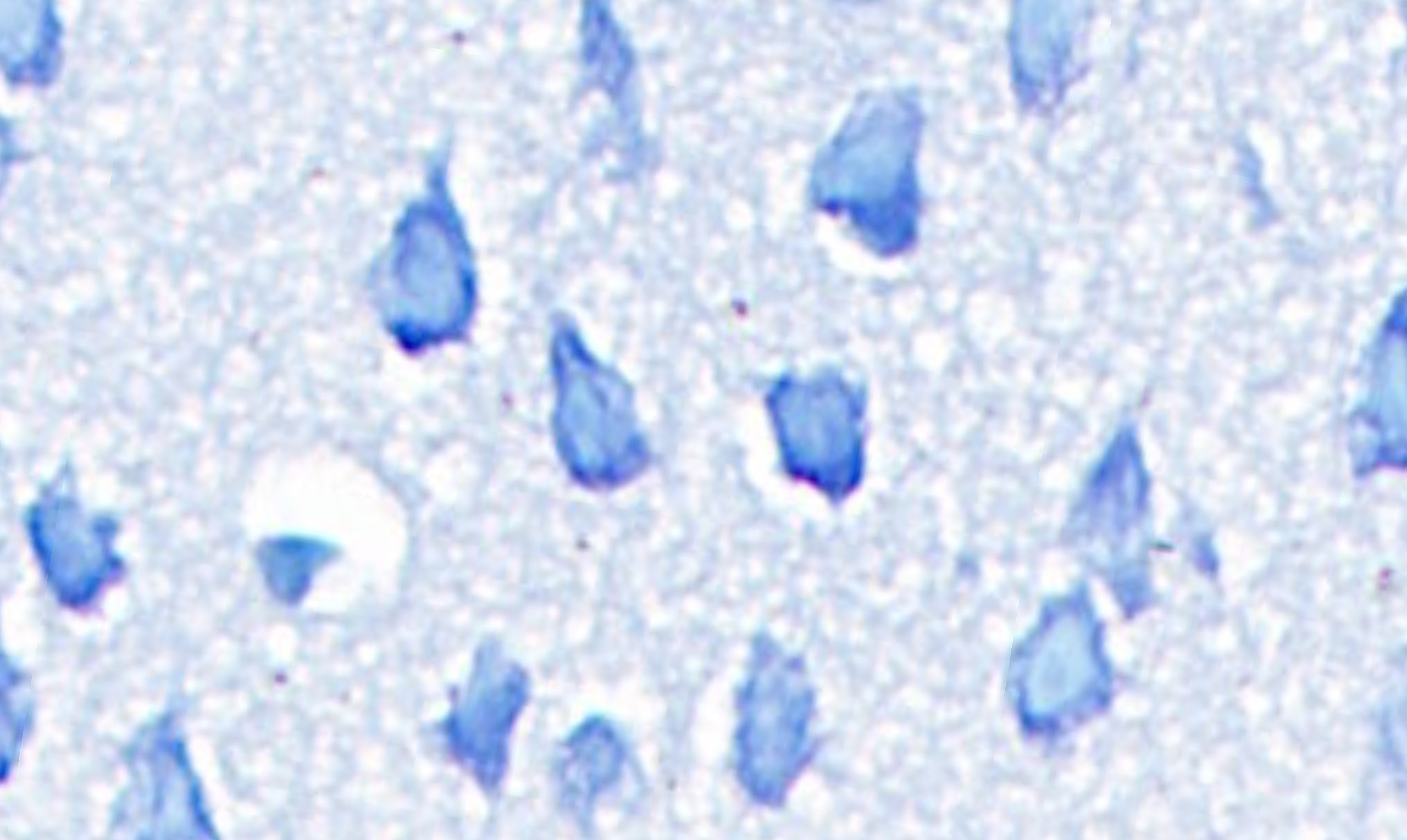

Supplement: Supplementary file 4 [file Presentation_4.ZIP › Nissle staining/ADS3 ─╘ ╟░╢ε ─ß╩╧_250.0x.jpg]

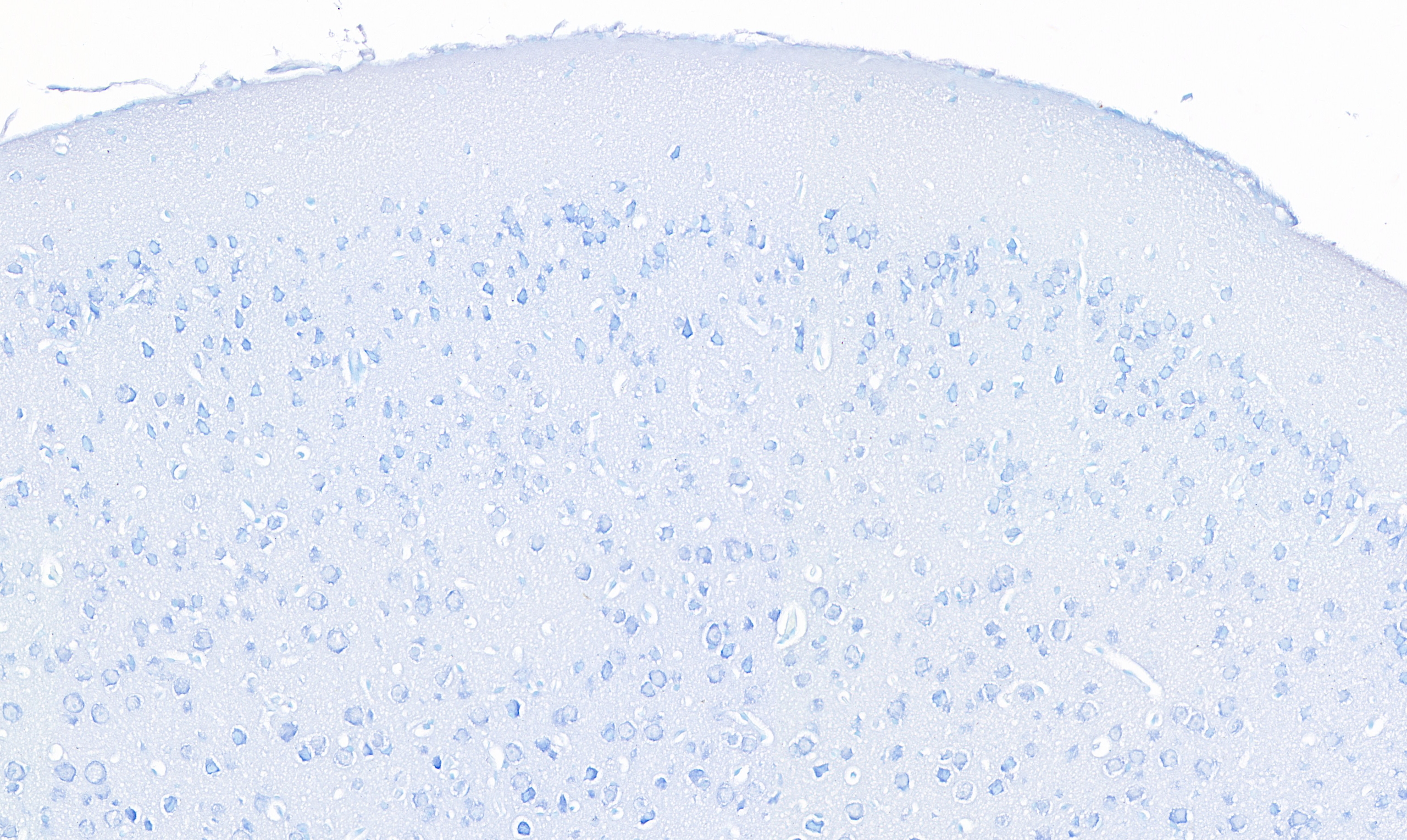

Supplement: Supplementary file 4 [file Presentation_4.ZIP › Nissle staining/WTE3 ─╘ ╟░╢ε ─ß╩╧_25.0x.jpg]

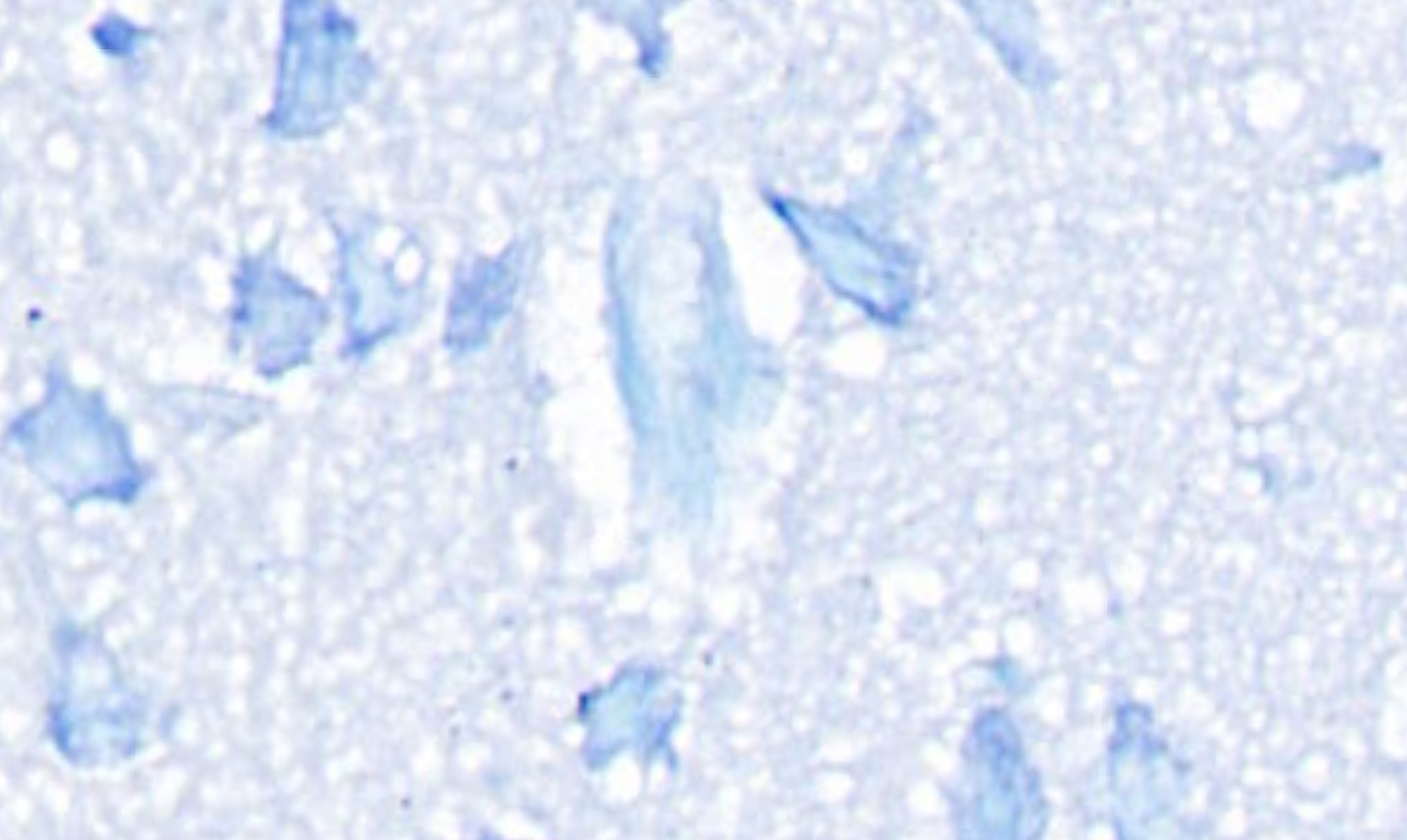

Supplement: Supplementary file 4 [file Presentation_4.ZIP › Nissle staining/WTE3 ─╘ ╟░╢ε ─ß╩╧_250.0x.jpg]

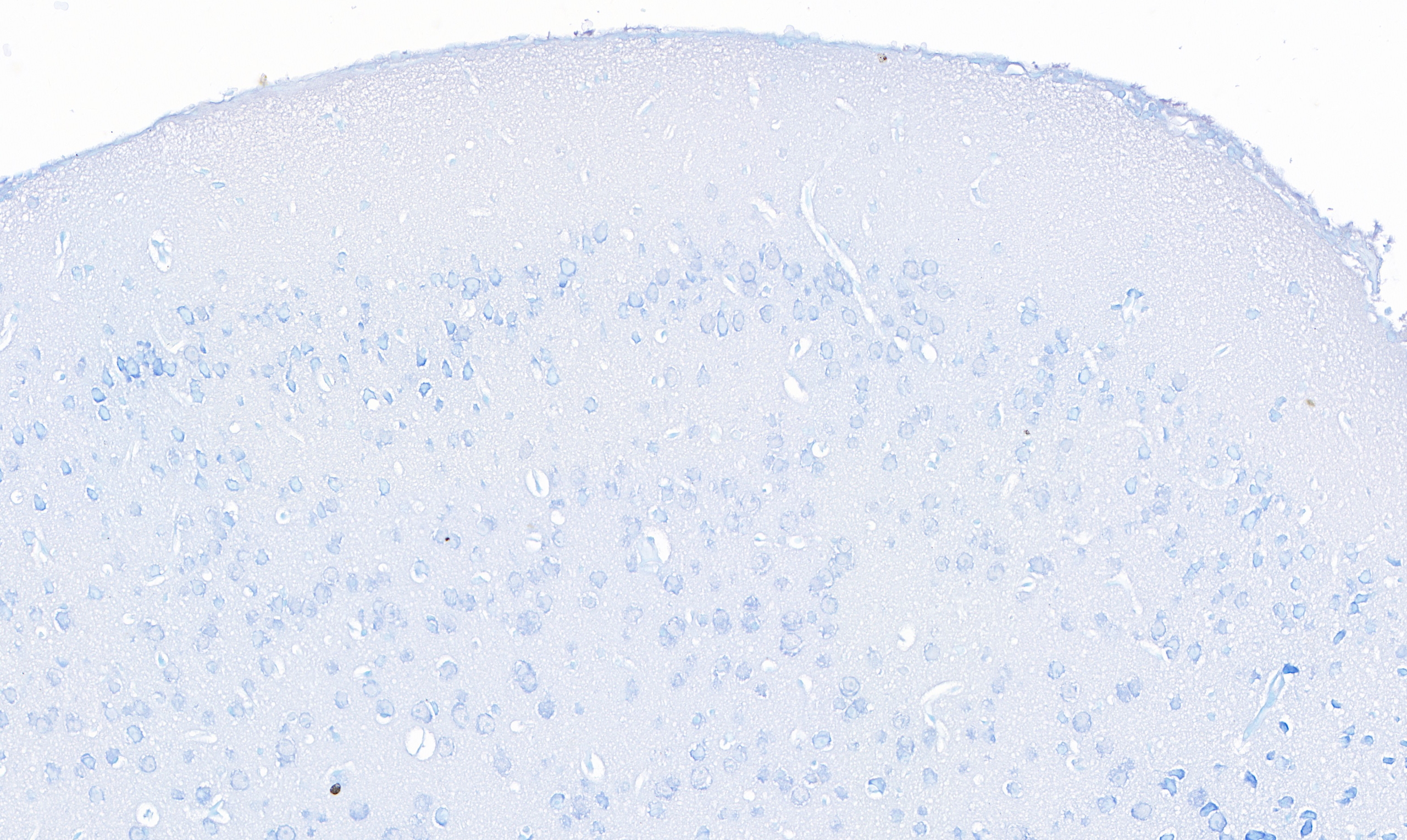

Supplement: Supplementary file 4 [file Presentation_4.ZIP › Nissle staining/WTS1 ─╘ ╟░╢ε ─ß╩╧_25.0x.jpg]

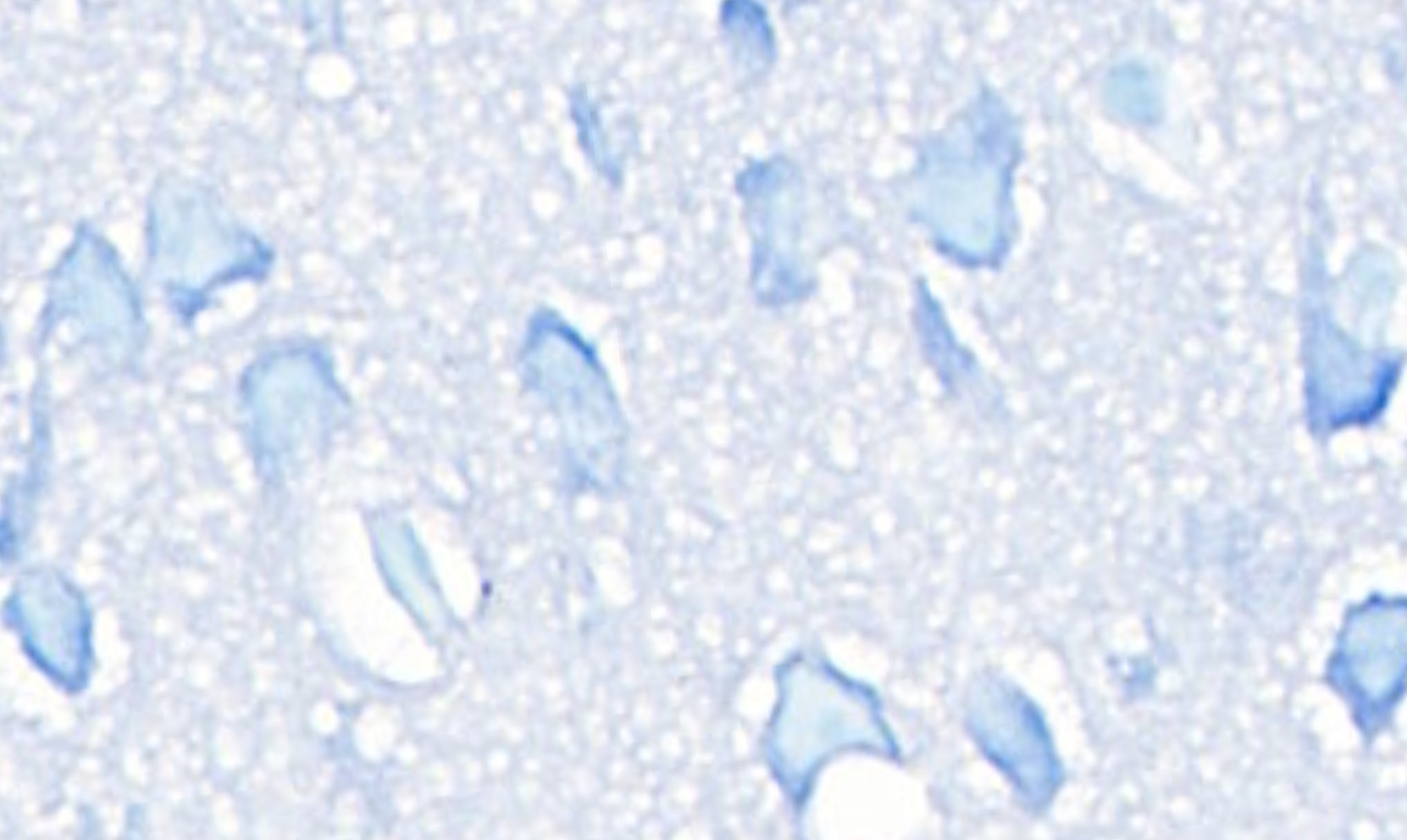

Supplement: Supplementary file 4 [file Presentation_4.ZIP › Nissle staining/WTS1 ─╘ ╟░╢ε ─ß╩╧_250.0x.jpg]

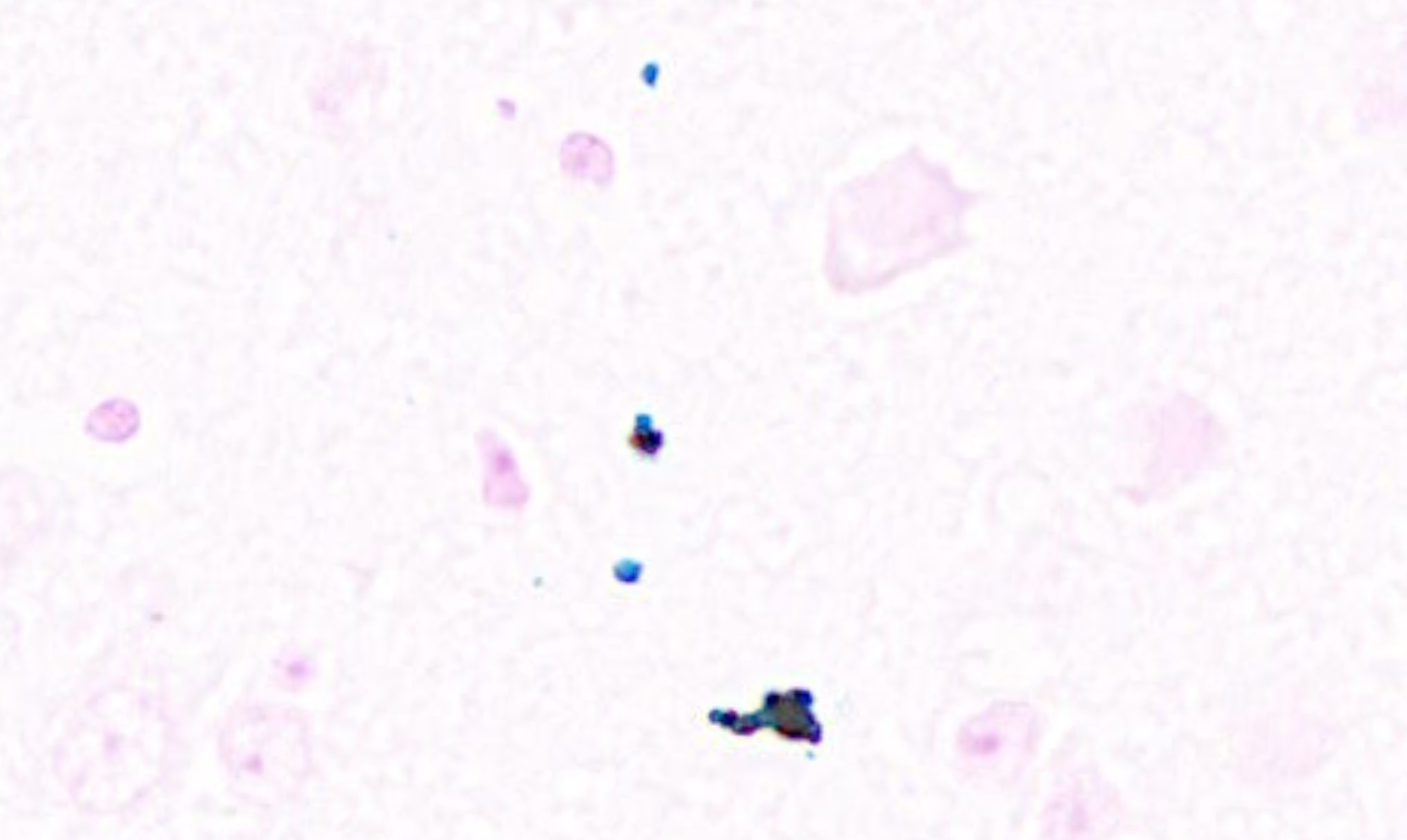

Supplement: Supplementary file 5 [file Presentation_5.ZIP › Prussian blue staining/ADE3 ─╘ ╟░╢ε ╞╒┬│╩┐└╢_200.0x.jpg]

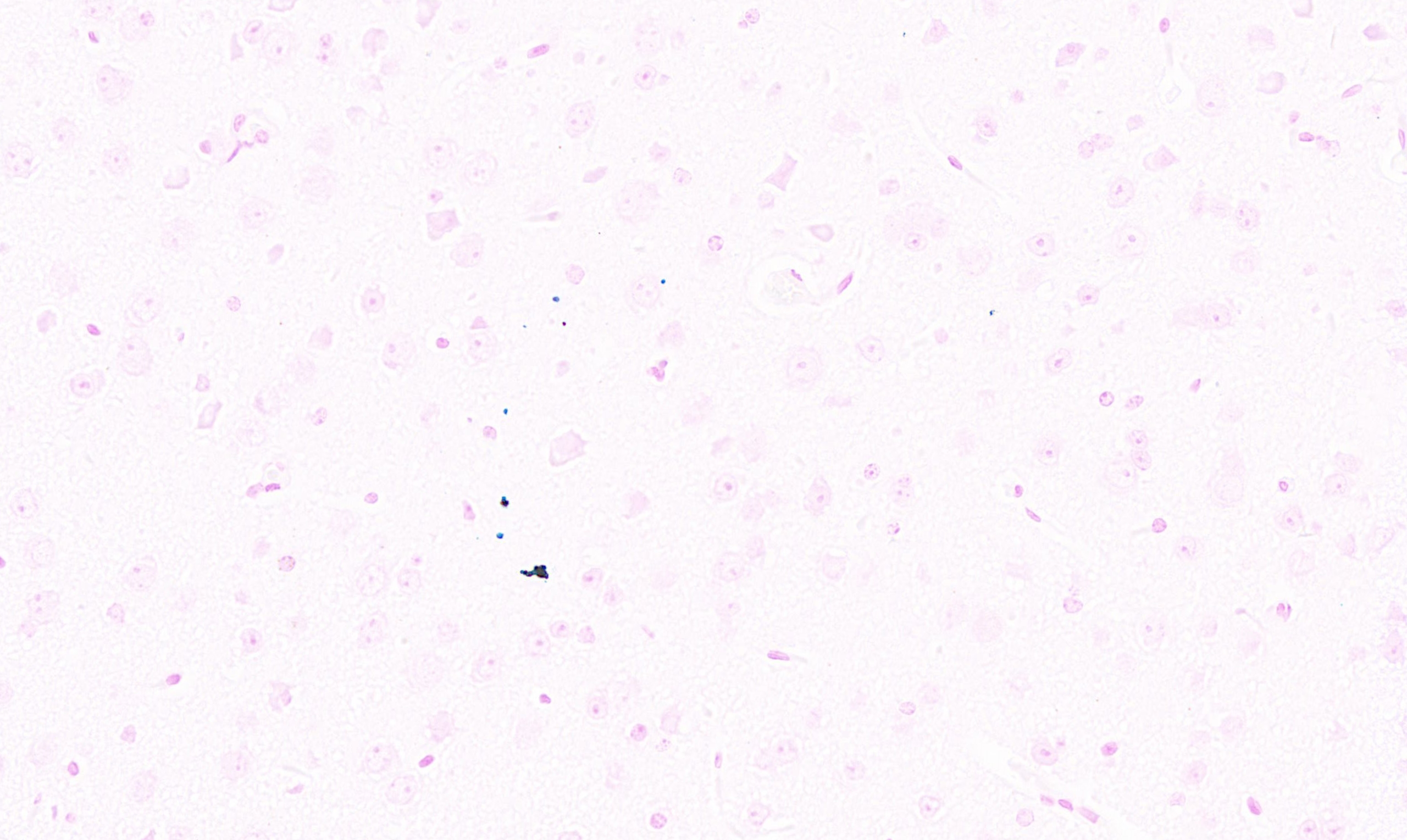

Supplement: Supplementary file 5 [file Presentation_5.ZIP › Prussian blue staining/ADE3 ─╘ ╟░╢ε ╞╒┬│╩┐└╢_50.0x.jpg]

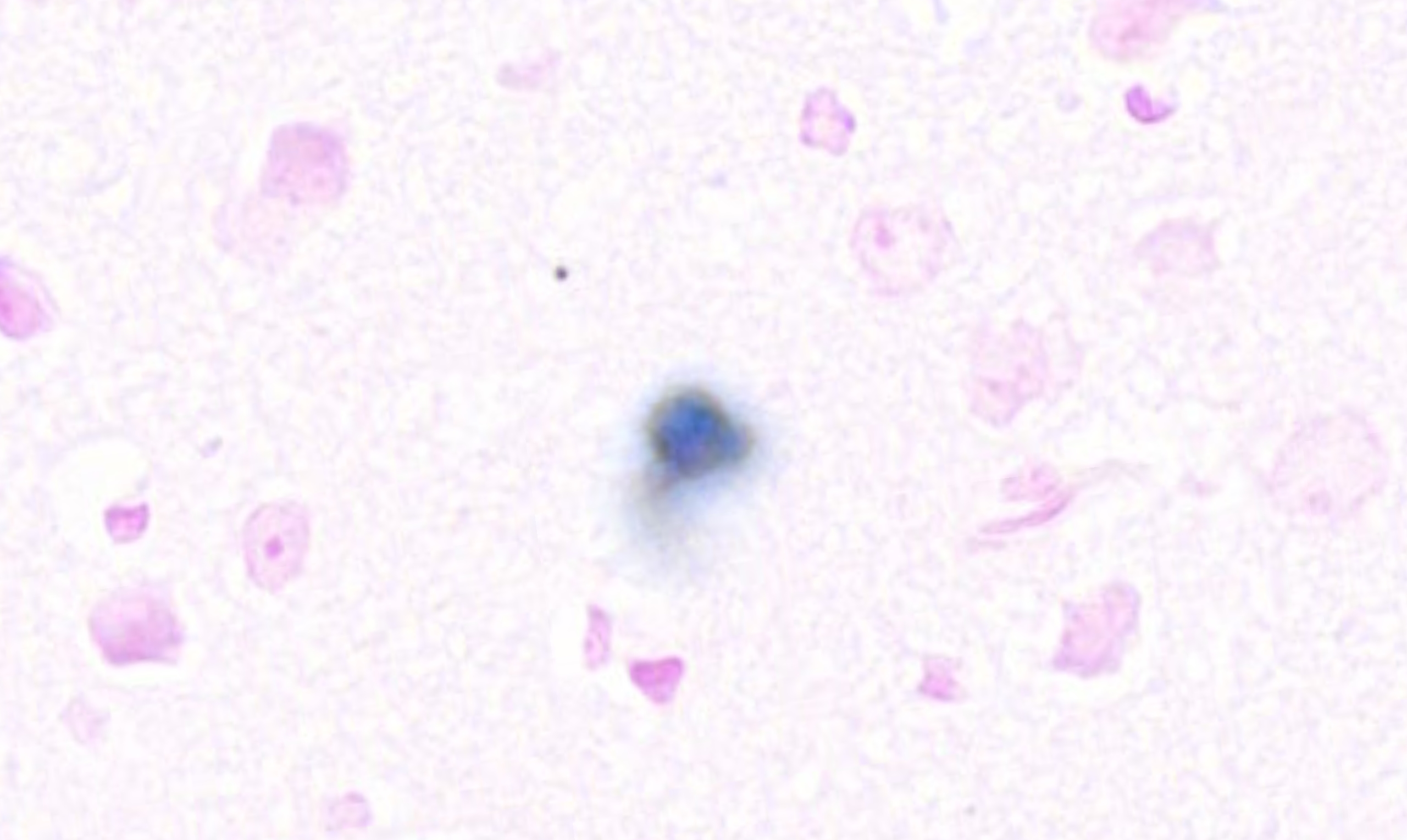

Supplement: Supplementary file 5 [file Presentation_5.ZIP › Prussian blue staining/ADS1 ─╘ ╟░╢ε ╞╒┬│╩┐└╢_200.0x.jpg]

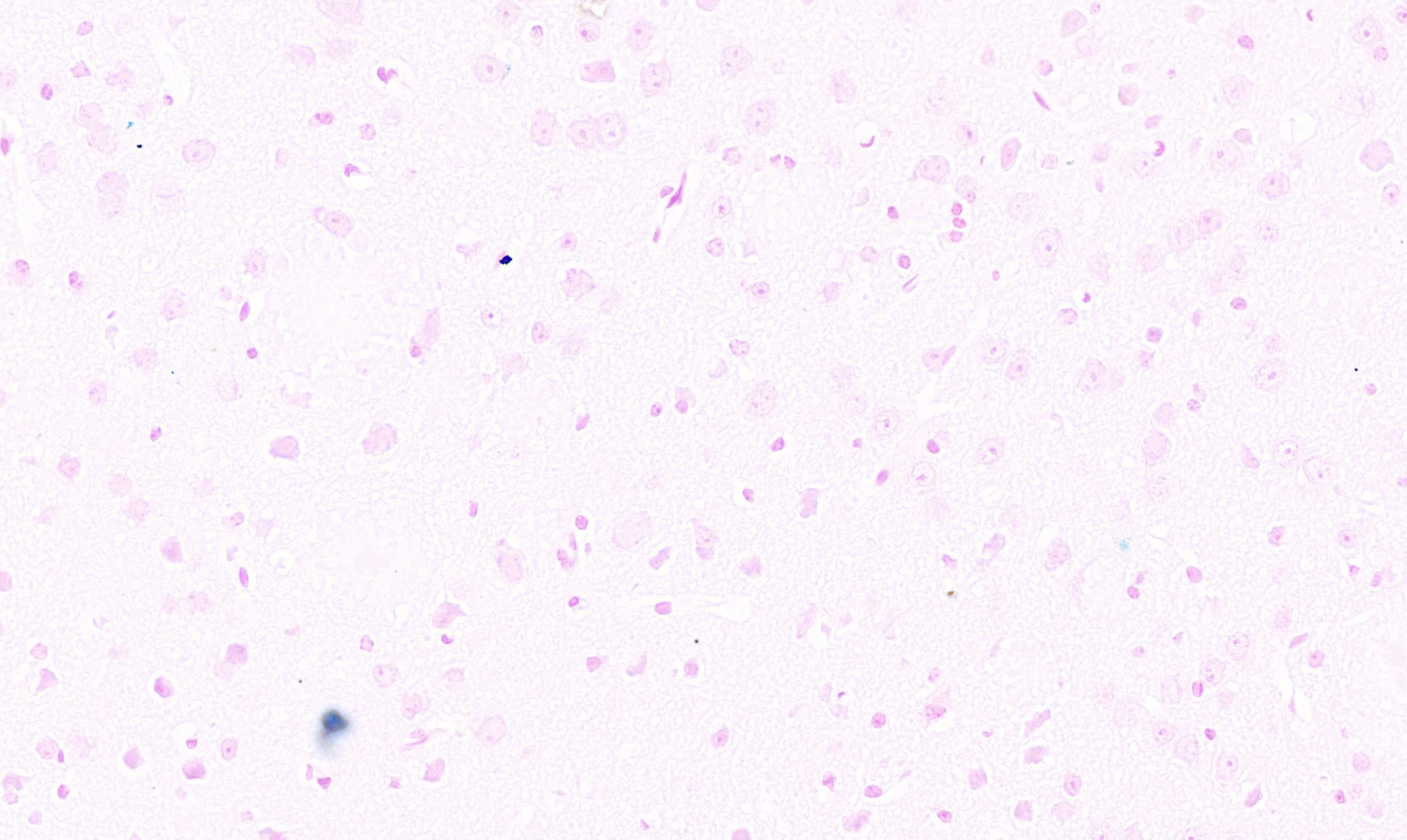

Supplement: Supplementary file 5 [file Presentation_5.ZIP › Prussian blue staining/ADS1 ─╘ ╟░╢ε ╞╒┬│╩┐└╢_50.0x.jpg]

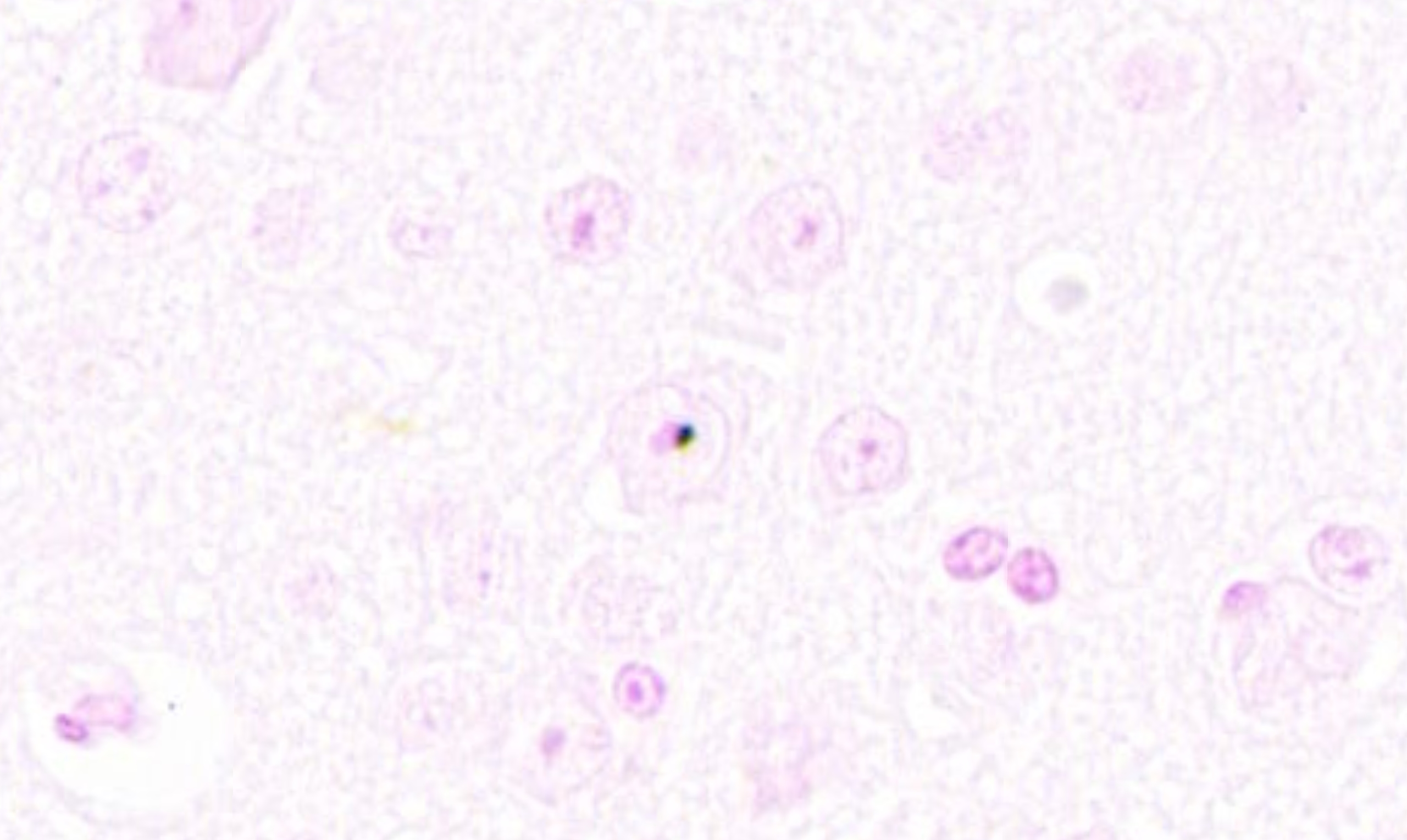

Supplement: Supplementary file 5 [file Presentation_5.ZIP › Prussian blue staining/WTE3 ─╘ ╟░╢ε ╞╒┬│╩┐└╢_200.0x.jpg]

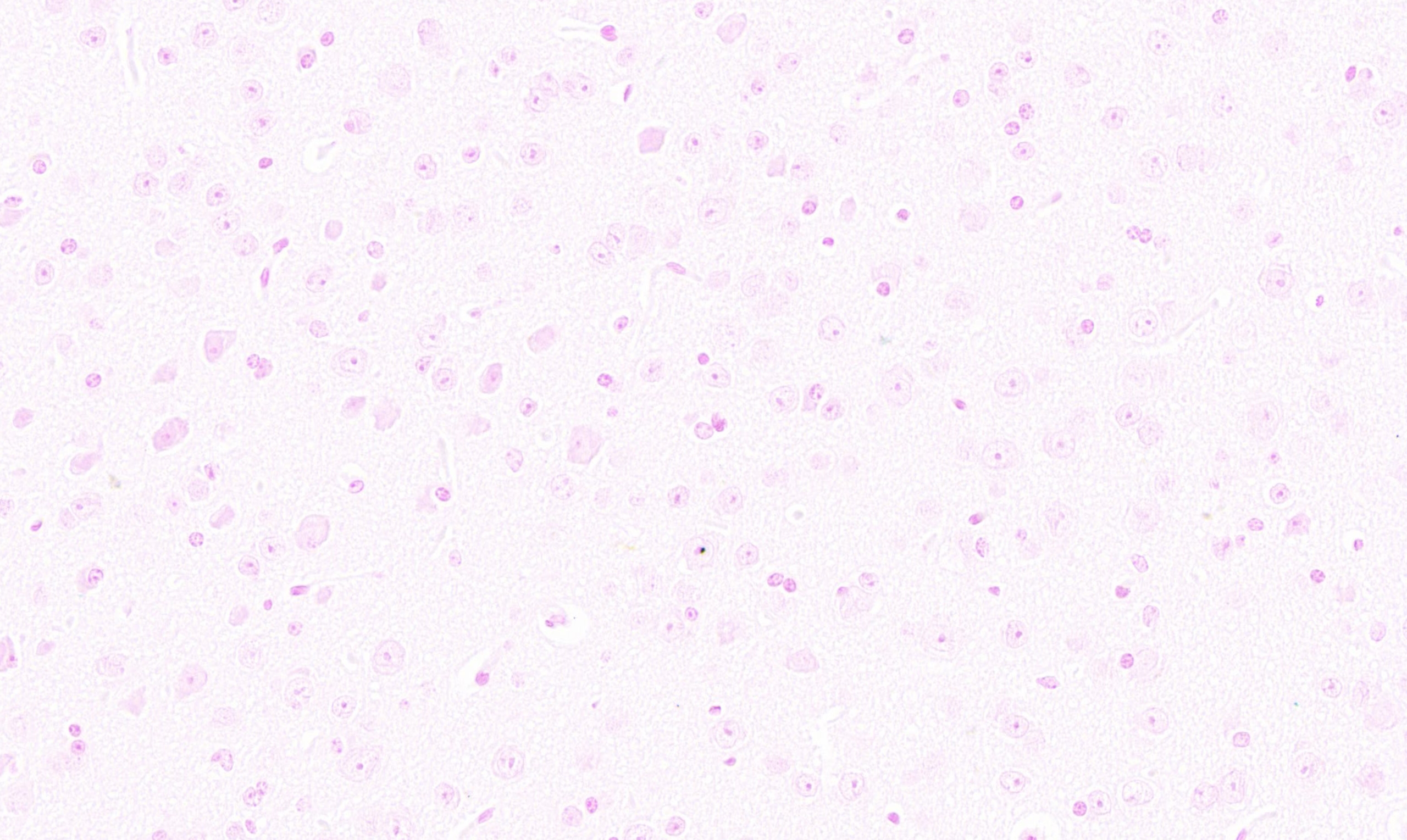

Supplement: Supplementary file 5 [file Presentation_5.ZIP › Prussian blue staining/WTE3 ─╘ ╟░╢ε ╞╒┬│╩┐└╢_50.0x.jpg]

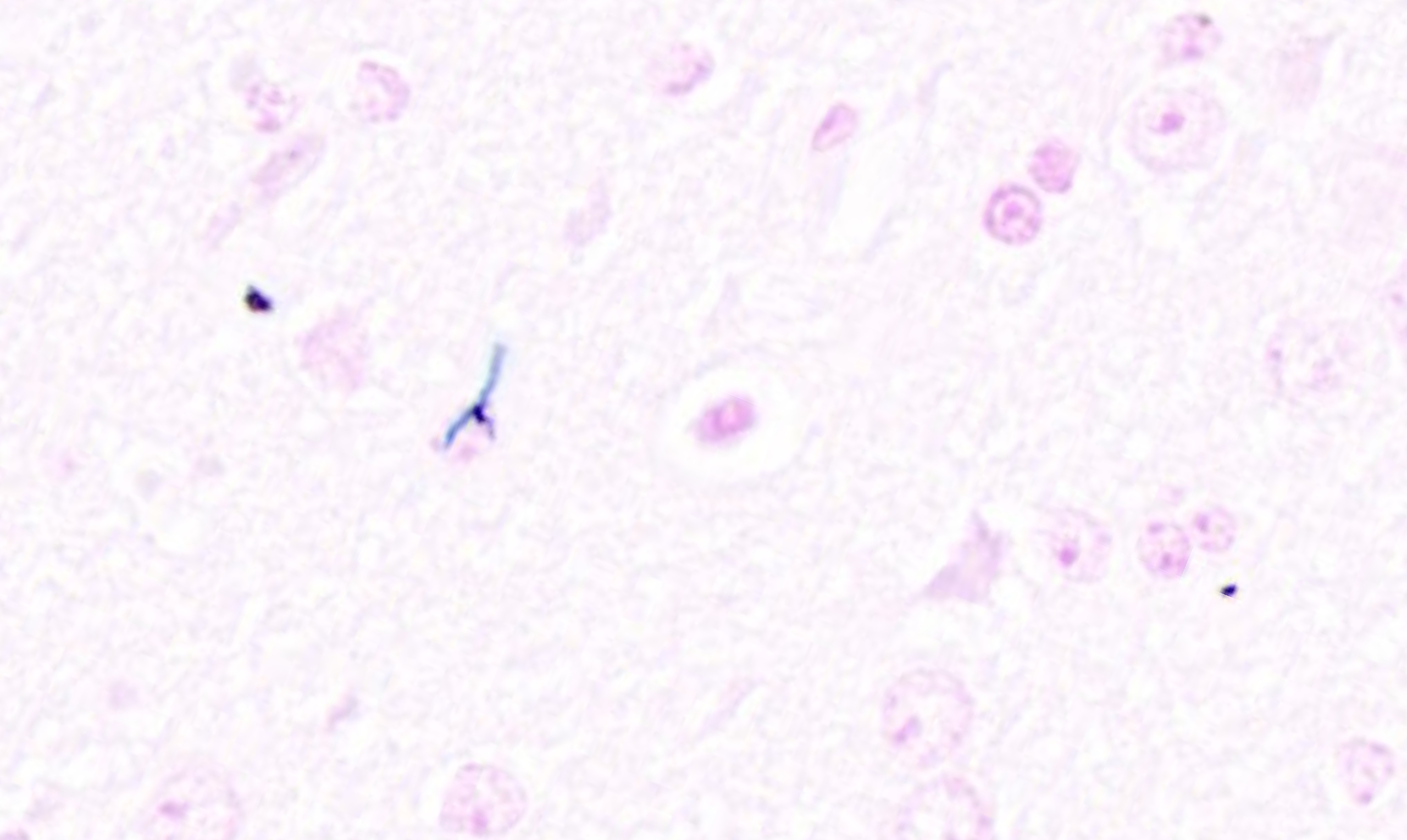

Supplement: Supplementary file 5 [file Presentation_5.ZIP › Prussian blue staining/WTS3 ─╘ ╟░╢ε ╞╒┬│╩┐└╢_200.0x.jpg]

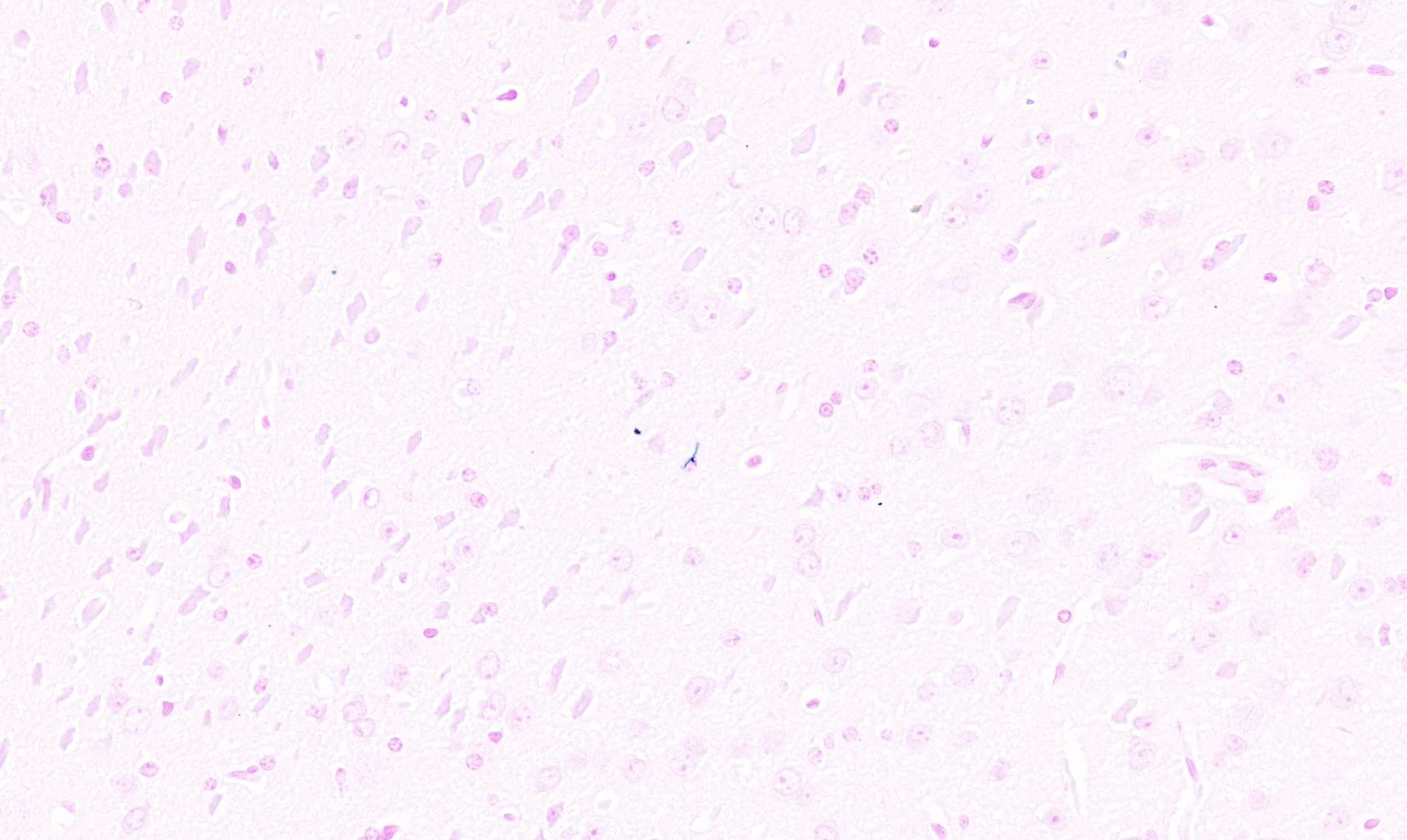

Supplement: Supplementary file 5 [file Presentation_5.ZIP › Prussian blue staining/WTS3 ─╘ ╟░╢ε ╞╒┬│╩┐└╢_50.0x.jpg]
